# A Cyaphide Transfer Reagent

**DOI:** 10.1021/jacs.1c04417

**Published:** 2021-06-30

**Authors:** Daniel W. N. Wilson, Stephanie J. Urwin, Eric S. Yang, Jose M. Goicoechea

**Affiliations:** Department of Chemistry, Chemistry Research Laboratory, University of Oxford, 12 Mansfield Road, Oxford OX1 3TA, U.K.

## Abstract

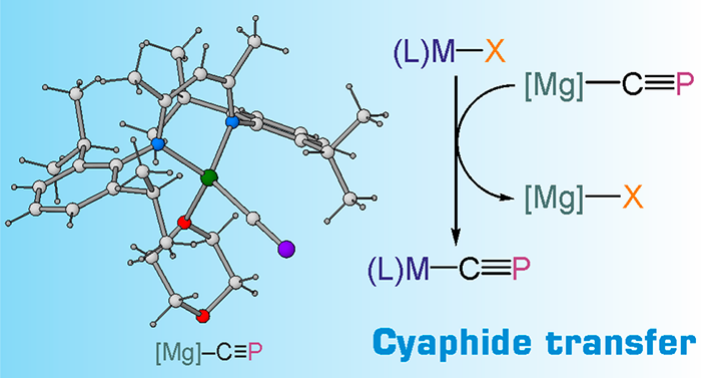

The cyanide ion plays a key role in a number of industrially relevant chemical processes, such as the extraction of gold and silver from low grade ores. Metal cyanide compounds were arguably some of the earliest coordination complexes studied and can be traced back to the serendipitous discovery of Prussian blue by Diesbach in 1706. By contrast, heavier cyanide analogues, such as the cyaphide ion, C≡P^–^, are virtually unexplored despite the enormous potential of such ions as ligands in coordination compounds and extended solids. This is ultimately due to the lack of a suitable synthesis of cyaphide salts. Herein we report the synthesis and isolation of several magnesium–cyaphido complexes by reduction of ^*i*^Pr_3_SiOCP with a magnesium(I) reagent. By analogy with Grignard reagents, these compounds can be used for the incorporation of the cyaphide ion into the coordination sphere of metals using a simple salt-metathesis protocol.

## Introduction

Along with the halide ions, cyanide (C≡N^–^) is one of the most ubiquitous anions in chemistry. Its salts are routinely used in industrial applications including bulk chemical synthesis, electroplating, metallurgy, tanning, manufacturing of paper and plastics, photography, and as fumigants and insecticides.^1^ In organic chemistry, it is an important functional group in nitriles (R–C≡N) and isonitriles (R–N≡C), many of which are produced on an industrial scale (e.g., adiponitrile, NC(CH_2_)_4_CN, which is used to produce nylon).^2,3^ By contrast, and despite the valence isoelectronic relationship between nitrogen and phosphorus, stable phosphorus-containing analogues of cyanides are much rarer; nitrile analogues, so-called phosphaalkynes (R–C≡P), have been known for almost 40 years and are highly reactive compounds due to the weak nature of C–P π bonds.^4,5^ Isocyanide analogues (R–P≡C) remain unknown.^6^ Unlike cyanide, which forms a multitude of stable salts, the cyaphide ion, C≡P^–^, cannot be obtained as a simple A(CP) or Ae(CP)_2_ salt (where A = alkali and Ae = alkaline-earth metal). To date, the C≡P^–^ ion has only ever been isolated in the coordination sphere of three metals (platinum, ruthenium, and uranium; e.g., *trans*-[Ru(dppe)_2_(H)(CP)] where dppe = bis(1,2-diphenylphosphinoethane)^7−12^ and an electrophilic borane.^13^ While these studies demonstrate that the ion is accessible, the resulting compounds are of limited synthetic utility due to their inertness. Alkali/alkaline-earth metal salts of the cyaphide ion are a more attractive target insomuch as they should allow for the incorporation of C≡P^–^ into novel molecules and solids, making use of salt metathesis protocols, a procedure that is well established for cyanides.^14^ Herein we show that well-defined alkaline-earth complexes of the cyaphide ion are readily accessible and can be used as anion transfer reagents for the synthesis of novel cyaphido complexes.

## Results and Discussion

**Figure 1:**
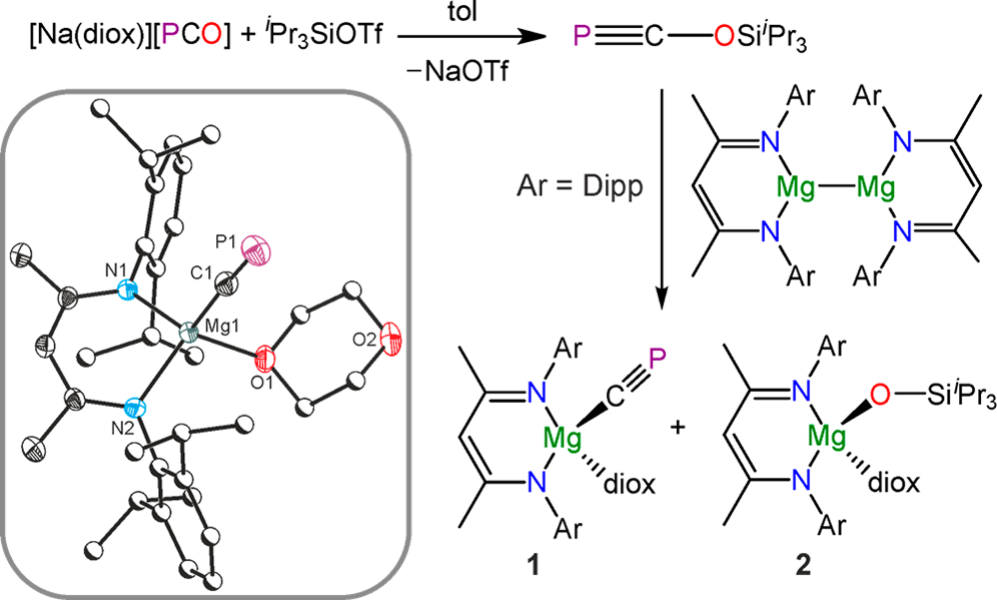
Synthesis of **1** and **2** from reduction of ^*i*^Pr_3_SiOCP. Inset: single crystal X-ray structure of **1** (anisotropic displacement ellipsoids set at 50% probability; hydrogen atoms omitted for clarity; carbon atoms of Dipp and dioxane are pictured as spheres of arbitrary radius).

The two-electron chemical reduction of the 2-phosphaethynolate ion, PCO^–^,^15^ to afford a uranium cyaphide complex was recently demonstrated by Meyer.^11^ We reasoned that functionalization of PCO^–^ to afford a phosphaethynolato compound (R–O–C≡P) would facilitate this reduction step, allowing for the straightforward generation of the cyaphide ion. A major limitation is that oxygen-functionalized phosphaethynolato compounds are rare and largely ionic in character.^11,16−21^ To date, only one species with significant covalent character has been structurally authenticated.^22^*In situ* silylation of the [Na(dioxane)_*x*_]PCO with tris(isopropyl)silyl trifluoromethanesulfonate in nonpolar aromatic solvents (benzene or toluene) favors silylation at the oxygen atom to afford the kinetic product ^*i*^Pr_3_SiOCP (Figure 1), which ultimately rearranges to give the κ-P isomer.^23^ Reduction of the former species using Jones’ magnesium(I) reagent [Mg(^Dipp^NacNac)]_2_^24,25^ cleanly affords an equimolar mixture of [Mg(^Dipp^NacNac)(CP)(dioxane)] (**1**) and [Mg(^Dipp^NacNac)(OSi^*i*^Pr_3_)(dioxane)] (**2**) where ^Dipp^NacNac = CH{C(CH_3_)N(Dipp)}_2_ and Dipp = 2, 6-di(isopropyl)phenyl (Figure 1). Density functional theory (DFT) calculations predicted this reaction to be exergonic (at 298.15 K) by 52.2 kcal mol^–1^ with an overall energy barrier of 12.5 kcal mol^–1^. The reaction proceeds via an unobserved dimagnesiated intermediate (a metalla-phosphaalkene) which rearranges by siloxyl group transfer (energy barrier of 4.4 kcal mol^–1^) to afford **1** and **2** (see the Supporting Information for the full computational analysis). Cleavage of the C–O bond in the phosphaethynolate ion necessitates a highly oxophilic two-electron reductant and significant steric protection (for example, when the less sterically encumbered magnesium(I) dimer [Mg(^Mes^NacNac)]_2_ (Mes = mesityl) was employed, the analogous reaction gave rise to a mixture of products including cyaphide oligomers).

The magnesium–cyaphido complex (**1**) exhibits a resonance in its ^31^P{^1^H} NMR spectrum at 177.2 ppm and a diagnostic singlet resonance in the ^1^H NMR spectrum corresponding to the ^Dipp^NacNac γ-proton at 4.78 ppm. A doublet resonance corresponding to the cyaphide ligand was observed in the ^13^C{^1^H} NMR spectrum at 270.97 ppm (^1^*J*_C–P_ = 34.0 Hz). Fractional crystallization of the reaction mixture allowed for the isolation and structural determination of compounds **1** and **2**.

The crystal structure of **1** (Figure 1, inset) revealed a linear arrangement of the Mg–C≡P moiety (177.37(15)°), with an Mg–C bond length of 2.118(2) Å, which is similar to that observed for other related compounds, such as [Mg(^Dipp^NacNac)(^*n*^Bu)(THF)] (2.127(2) Å).^26^ The C–P bond length in **1** is 1.553(2) Å, in line with the predicted value for a carbon–phosphorus triple bond (1.54 Å),^27^ and similar to reported values for other metal–cyaphide complexes (cf. 1.573(2) Å in *trans*-[Ru(dppe)_2_(H)(CP)]).^9^ The crystal structure of **2** can be found in the Supporting Information.

*In situ* generated mixtures of **1** and **2** can be used to transfer the cyaphide ion to metal complexes (*vide infra*) via a salt metathesis protocol, in a manner reminiscent of Grignard reagents.^28^ However, because of the similar solubility of **1** and **2** in common laboratory solvents, the isolation of compositionally pure samples of **1** is only possible in low yields (∼20%).

**Scheme 1:**
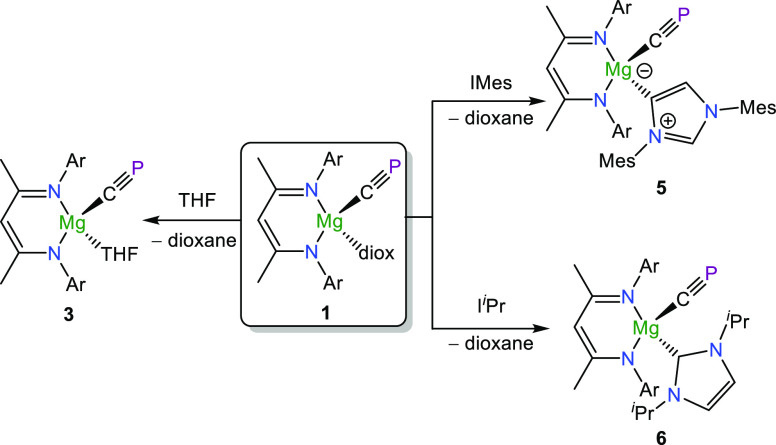
Reactivity of **1** toward Lewis Bases (Ar = Dipp)

Thus, we sought strategies to modify the solubility of **1**. Quantitative dioxane displacement was achieved by using THF-*d*_8_ to form [Mg(^Dipp^NacNac)(CP)(THF-*d*_8_)] (**3**; see Scheme 1); however, this adduct is equally difficult to separate from the siloxymagnesium side-product. In addition, it was observed for both solvent adducts **1** and **3** that exposure to vacuum initiated decomposition of the target compounds, evidenced by broadening of NMR spectra (Figure S11). We hypothesize that initial cleavage of the Mg–solvent interaction forms the base-free analogue [Mg(^Dipp^NacNac)(CP)]_*x*_ (**4**), which then rapidly decomposes. Employing dioxane-free Na(OCP) during the generation of ^*i*^Pr_3_SiOCP subsequently led to the specific formation of solvent-free analogue **4**, evidenced by a ^31^P{^1^H} NMR singlet resonance at 246.7 ppm. The solubility of this desolvated analogue is sufficiently lower than **2** to facilitate efficient separation by precipitation; however, in the solid state **4** is unstable, decomposing rapidly once isolated (see the Supporting Information for further details). The structure of **4** is currently unknown, but the downfield shifted ^31^P NMR resonance suggests it is oligomeric; the related solvent-free cyanido complex, [Mg(^Dipp^NacNac)(CN)]_3_, is a cyclic trimer.^29^ Addition of dioxane or THF-*d*_8_ to solutions of **4** resulted in the formation of the corresponding solvated adducts **1** or **3**, respectively.

To circumvent problematic cleavage of the coordinated base under vacuum, our attention turned to nonvolatile Lewis donors. Addition of excess pyridine or 4-(dimethylamino)pyridine (DMAP) to a C_6_D_6_ solution of **1** did not result in displacement of the dioxane molecule. Addition of *N*-heterocyclic carbenes (NHCs) IMes and I^*i*^Pr (IMes =1,3-dimesitylimidazol-2-ylidene; I^*i*^Pr = 1,3-diisopropylimidazol-2-ylidene) successfully afforded carbene adducts [Mg(^Dipp^NacNac)(CP)(IMes)] (**5**) and [Mg(^Dipp^NacNac)(CP)(I^*i*^Pr)] (**6**), which unlike solvent adducts **1** and **3** can both be isolated as compositionally pure solids in moderate yields (**5**: 54%; **6**: 48%) and can be bottled and stored for several weeks under a nitrogen atmosphere without degradation (Scheme 1). No reaction was observed between the NHCs and the siloxy byproduct **2**.

**Figure 2:**
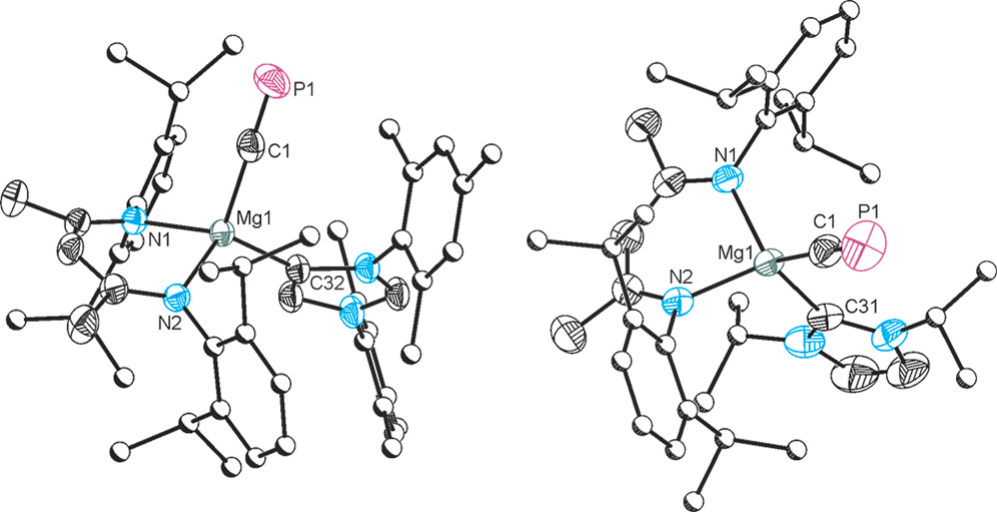
Single-crystal X-ray structures of **5** (left) and **6** (right). Anisotropic displacement ellipsoids set at 50% probability. Hydrogen atoms are omitted for clarity. Carbon atoms of Dipp and ^*i*^Pr groups are pictured as spheres of arbitrary radius.

Spectroscopically **5** and **6** do not differ greatly from compound **1** and exhibit comparable NMR shifts [e.g., ^31^P{^1^H} NMR: 162.9 (**4**); 174.9 ppm (**5**)]. Both NHC adducts were structurally authenticated by single-crystal X-ray diffraction (Figure 2) and confirm the expected association of the NHC with the magnesium metal center. The Mg–CP (**5**: 2.166(2); **6**: 2.144(3) Å) and C–P bonds (**5**: 1.550(2); **6**: 1.531(3) Å) for both compounds are in line with those observed for **1**. Interestingly, it was found that while the IMes carbene associated with the magnesium metal center in an “abnormal” fashion (i.e., through the alkenic backbone), the I^*i*^Pr carbene adduct coordinates as expected in the solid state, an observation we put down to the increased steric bulk of IMes.^30^ However, in solution the I^*i*^Pr moiety of **6** fluctuates between normal and abnormal coordination. The ^31^P{^1^H} NMR spectrum of **6** at room temperature features a particularly broad singlet signal (υ_1/2_ ≈ 224 Hz) which when cooled below −20 °C gives rise to two sharper singlet signals at 173.3 ppm (major) and 167.7 ppm (minor) (Figure S19). These correspond to the normal and abnormal coordination mode of the I^*i*^Pr moiety, respectively, in good agreement with the DFT calculated chemical shifts (175.4 and 172.3 ppm; Table S6). Calculations further indicate that the difference in energy between these two isomers is negligible. **1** does not react with IMes^Me^ and I^*i*^Pr^Me^, analogous NHCs featuring methylated backbones where abnormal coordination is blocked.

Compound **5** cocrystallizes with one stoichiometric equivalent of IMes as a cocrystal, which is also seen in the ^1^H NMR spectrum of the bulk product. Probing further, we calculated no energy payoff for the displacement of the dioxane by IMes (1–3 kcal mol^–1^, within error), with neither compound being thermodynamically favored between −100 and 100 °C. The calculated thermodynamic data are indicative of an equilibrium. However, even when just one equivalent of IMes is added to a solution of **1**, **5**·IMes can be isolated from the reaction mixture by crystallization (albeit in lower yields). This indicates that the additional molecule of IMes present in the lattice is critical to isolate crystalline samples of **5**.

Given the ionic nature of the Mg–CP bond in **1**, we reasoned that salt metathesis reactions between this species and main-group or metal halides would allow for cyaphide group transfer. This hypothesis was probed by addition of chlorotrimethylsilane to a C_6_D_6_ solution of **1** formed *in situ*. The ^31^P{^1^H} NMR spectrum showed a single resonance at 97.9 ppm, corresponding to the known phosphaalkyne Me_3_SiCP.^31^ This clean, quantitative transfer of the CP^–^ ion is, to our knowledge, the first instance of such reactivity. Encouraged by this finding, we targeted novel cyaphide–metal complexes. It is worth noting at this stage that comparable cyaphide transfer reactions are also possible with compounds **5** and **6**.

**Scheme 2:**
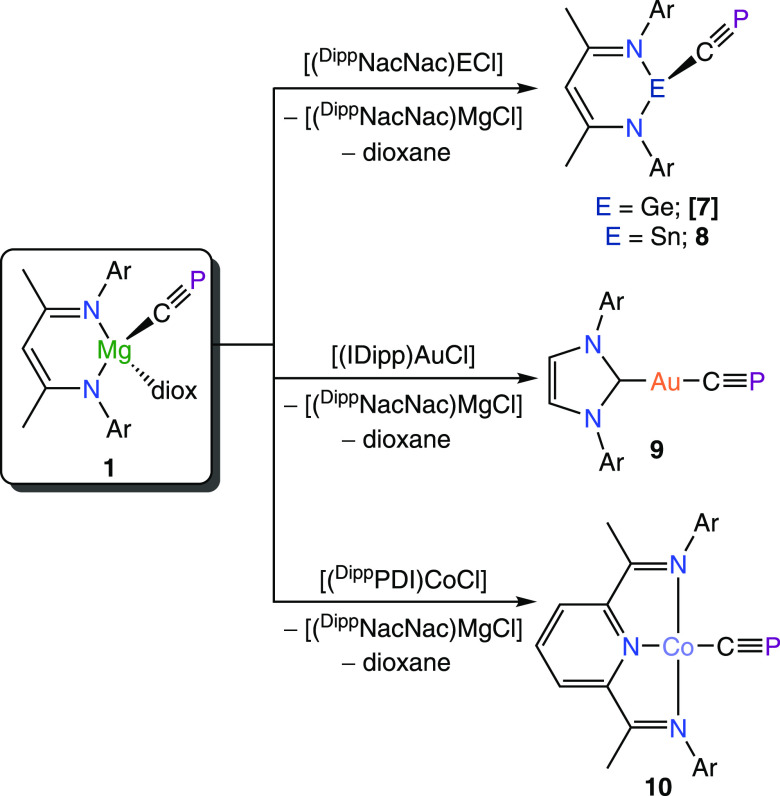
Reactivity of **1** toward Metal Halides (Ar = Dipp)

Moving to heavier group 14 elements, addition of [Ge(^Dipp^NacNac)Cl] to an *in situ* generated mixture of **1** and **2** leads to rapid consumption of **1** and a new ^31^P{^1^H} NMR signal at 106.4 ppm (Scheme 2). A new singlet signal in the corresponding ^1^H NMR spectrum at 5.08 ppm, within the characteristic region for γ-H protons, indicates a new ^Dipp^NacNac environment. Also evident was that siloxy byproduct **2** remained unreacted (Figure S20). Over the course of a few hours, this deep-red solution, presumably containing [Ge(^Dipp^NacNac)(CP)] (**7**), changed to a dark-green color, and NMR spectroscopy showed decomposition of the metal–cyaphide complex into multiple phosphorus-containing compounds, a process which was accelerated by any physical manipulation.

**Figure 3:**
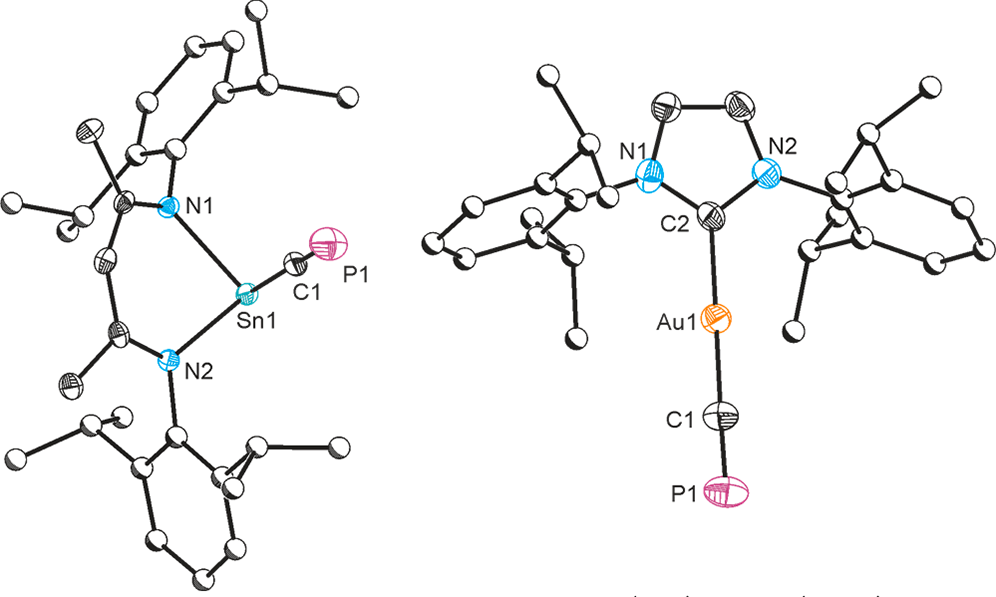
Single-crystal X-ray structures of **8** (left) and **9** (right). Anisotropic displacement ellipsoids set at 50% probability. Hydrogen atoms are omitted for clarity. Carbon atoms of Dipp groups are pictured as spheres of arbitrary radius.

Reaction of **1** with [Sn(^Dipp^NacNac)Cl] led to the formation of [Sn(^Dipp^NacNac)(CP)] (**8**), which can be isolated by fractional crystallization. Cyaphide transfer was first indicated by ^31^P{^1^H} NMR spectroscopy which revealed a new resonance with P–Sn coupling satellites at 122.4 ppm (^2^*J*_P–Sn_ = 69.8 Hz) and confirmed in the solid-state structure (Figure 3). The C≡P bond is intact and comparable (1.542(4) Å) to those of **1**, **5**, and **6**. At 2.216(4) Å, the Sn–C bond is relatively long, and the Sn–C≡P unit is practically linear (179.16°). No resonance could be found in the ^13^C NMR spectrum of **8** corresponding to the cyaphide group which we attribute to broadening due to coupling to two adjacent NMR active nuclei. A weak band was observed in the IR spectrum of **8** at 1321 cm^–1^, which is consistent with the predicted value (1327 cm^–1^), but partially masked by a band arising from a Dipp C=C stretching mode.

Having demonstrated intermetallic cyaphide transfer through halide metathesis, our next target was to prepare transition metal–cyaphide complexes. Reaction of an *in situ* generated mixture of **1** and **2** with [Au(IDipp)Cl] resulted in the formation of the gold–cyaphido complex [Au(IDipp)(CP)] (**9**) which exhibits a singlet resonance in its ^31^P{^1^H} NMR spectrum at 84.1 ppm (Scheme 2). This is at a significantly lower frequency than observed for compound **1** (177.2 ppm) and slightly lower than **7** and **8** (106.4 and 122.4 ppm), consistent with an increase in the covalent character of the Au–CP bond.^32−34^ The ^13^C{^1^H} resonance observed for the cyaphide group in **8**, 247.70 ppm (^1^*J*_C–P_ = 6.1 Hz), is also at a lower frequency compared to **1** (270.97 ppm). The single-crystal X-ray structure of **9** (Figure 3) reveals a linear two-coordinate gold center (C–Au–C = 178.2(2)°; Au–C–P = 178.0(5)°) with Au–C_carbene_ and Au–CP distances of 2.034(6) and 1.972(6) Å, respectively. The former of these is slightly elongated compared to that observed in the [(IDipp)AuCl] starting material (1.942(3) Å) due to the stronger σ-donor ability of the cyaphide ion.^35^ This distance is more comparable with NHC gold acetylene or cyanido complexes such as [Au(IDipp)(C≡CPh)] (2.018(7) Å) or [Au(IDipp)(CN)] (1.985(15) Å).^36^ The C–P bond distance in **9** is 1.552(6) Å, which is comparable to the other cyaphide complexes discussed thus far (cf. 1.553(2) Å for **1**). The IR spectrum of compound **9** reveals a band at 1342 cm^–1^, which is higher than the value reported for *trans*-[Ru(dppe)_2_(H)(CP)] (1229 cm^–1^) and thus indicative of little π-back-bonding; however, it is worth noting that this vibrational mode is heavily coupled with the Au–C_carbene_ stretch on account of the linear coordination geometry of **9**.

**Figure 4:**
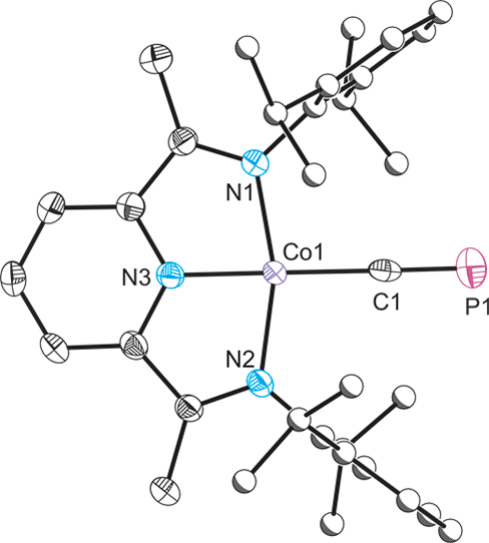
Single-crystal X-ray structure of **10**. Anisotropic displacement ellipsoids set at 50% probability. Hydrogen atoms are omitted for clarity. Carbon atoms of Dipp groups are pictured as spheres of arbitrary radius.

Finally, in an effort to illustrate the broad synthetic utility of the cyaphide transfer reagent **1**, we sought to synthesize the first example of a 3d metal–cyaphide complex. For this purpose we reacted a mixture of **1** and **2** with [(^Dipp^PDI)CoCl] (^Dipp^PDI = 2,6-{2,6-^*i*^Pr_2_C_6_H_3_NCMe}_2_C_5_H_3_N). The reaction results in the clean quantitative formation of a new product, [(^Dipp^PDI)Co(CP)] (**10**), which exhibits a single resonance in its ^31^P{^1^H} NMR spectrum at 345.4 ppm. This is notably downfield from all of the other known cyaphide complexes, presumably due to a greater paramagnetic contribution to the NMR shielding constant (σ). Our calculations support this, predicting a δ value of 341 ppm for the ^31^P NMR chemical shift. The ^1^H and ^13^C{^1^H} NMR spectra for **10** are in line with the presence of a single ^Dipp^PDI ligand. Notably, we were unable to observe the NMR resonance for the cyaphide ligand in the ^13^C{^1^H} NMR spectrum due to coupling with the ^31^P and quadrupolar ^59^Co nuclei. [(PDI)Co(R)] complexes have previously been attributed biradical character which explains anomalous ^1^H NMR shifts, which we also see in [Co(^Dipp^PDI)(CP)] (e.g., the imine NCC*H*_3_ protons are upfield shifted to −0.23 ppm).^37^ The IR spectrum of compound **10** reveals a band at 1306 cm^–1^, in between the values observed for **9** (1342 cm^–1^) and *trans*-[Ru(dppe)_2_(H)(CP)] (1229 cm^–1^), suggesting a moderate degree of π-backbonding (as expected for a first row transition metal). Extremely air- and moisture-sensitive blue crystals of **10** were obtained from a concentrated toluene solution at −35 °C. The structure of **10** was unequivocally confirmed by single-crystal X-ray diffraction (Figure 4) which reveals a square-planar cobalt(I) compound bonded to a cyaphide ligand.

## Conclusion

The reductive deoxygenation of ^*i*^Pr_3_SiOCP offers an efficient route to cyaphide generation at a magnesium center. This reactive salt is the first example of a cyaphide transfer reagent, allowing for the synthesis of novel metal–cyaphido complexes using Grignard-like reactivity (including the first examples of square-planar, trigonal pyramidal and linear complexes of the cyaphide ligand). This new reagent will undoubtedly open up new avenues in the coordination chemistry of metal complexes and may ultimately be used for the synthesis of novel extended solids featuring the cyaphide ion such as, for example, Prussian Blue analogues.

## Experimental Section

### General Procedures

All reactions and product manipulations were performed under an inert atmosphere of argon or dinitrogen by using standard Schlenk-line or glovebox techniques (MBraun UNIlab glovebox maintained at <0.1 ppm of H_2_O and <0.1 ppm of O_2_). Generation of ^*i*^Pr_3_SiOCP was adapted from the previously reported synthesis.^23^ Na[PCO(dioxane)_5.6_],^38^ [Mg(^Dipp^NacNac)]_2_,^39^ IMes,^40^ I^*i*^Pr,^41^ [Ge(^Dipp^NacNac)Cl],^42^ [Sn(^Dipp^NacNac)Cl],^42^ [(IDipp)AuCl],^35^ and [Co(^Dipp^PDI)Cl]^43^ were synthesized according to previously reported synthetic procedures. Triisopropylsilyl trifluoromethanesulfonate (Sigma-Aldrich) and chlorotrimethylsilane (Sigma-Aldrich) were used as received. Hexane (hex; Sigma-Aldrich, HPLC grade) and toluene (Sigma-Aldrich, HPLC grade) were purified by using an MBraun SPS-800 solvent system. C_6_D_6_ (Aldrich, 99.5%) was dried over CaH_2_ and degassed prior to use. THF (Sigma-Aldrich, HPLC grade) and THF-*d*_8_ (Sigma-Aldrich, 99.5%) were distilled over sodium/benzophenone. All dry solvents were stored under argon in gastight ampules. Additionally, solvents were stored over activated 3 Å molecular sieves.

### Characterization Techniques

NMR spectra were acquired on Bruker AVIII 500 MHz (^1^H 500 MHz, ^13^C 126 MHz) and Bruker AVIII 400 MHz NMR spectrometers (^31^P 162 MHz) at 295 K unless otherwise stated. ^1^H and ^13^C NMR spectra were referenced to residual protic solvent resonance (^1^H NMR C_6_D_6_: δ = 7.16 ppm; ^13^C NMR C_6_D_6_: δ = 188.06 ppm). ^31^P and ^119^Sn were externally referenced to an 85% solution of H_3_PO_4_ in H_2_O and SnMe_4_, respectively. Elemental analyses were performed by Elemental Microanalyses Ltd. (Devon, UK). Samples (∼5 mg) were submitted in sealed glass vials.

### Synthesis of [Mg(^Dipp^NacNac)(CP)(dioxane)] (**1**) and [Mg(^Dipp^NacNac)(OSi^i^Pr_3_)(dioxane)] (**2**)

Inside a glovebox, ^*i*^Pr_3_SiOTf (90 mg, 0.29 mmol) was dissolved in toluene (ca. 0.5 mL) and added to a vial containing [Na(dioxane)_5.6_]PCO (167 mg, 0.29 mmol). The resulting suspension was stirred for 4 h to generate ^*i*^Pr_3_SiOCP, with occasional washing of the walls of the vial to ensure complete consumption of the starting materials. The resulting mixture was filtered through a glass paper filter, and the solids were washed with a small amount of toluene. [Mg(^Dipp^NacNac)]_2_ (195 mg, 0.22 mmol) was added as a solid to the resulting yellow solution, causing it to darken to orange. Reaction completion was confirmed by ^31^P{^1^H} NMR spectroscopy. The solution can be used as an *in situ* supply of **1** with an equimolar amount of **2** also present; a representative ^1^H NMR of such a solution can be found in Figure S1. Concentration of the solution (taking care to avoid evaporation to dryness) afforded a red oil. The residue was extracted into hexane (1 mL) and filtered. Cooling the orange solution to −35 °C overnight yielded **1** as yellow crystals suitable for X-ray diffraction (29 mg, 22% yield). Further concentrating the solution, or cooling for longer periods, resulted in mixtures of **1** and **2**.

### [Mg(^Dipp^NacNac)(CP)(dioxane)] (**1**)

Anal. Calcd (%) for C_34_H_49_MgN_2_O_2_P: C, 71.26; H, 8.62; N, 4.89. Found: 71.35; H, 9.15; N, 4.82. ^1^H NMR (500 MHz, C_6_D_6_): δ (ppm) = 7.11–7.09 (br m, 6H; Dipp ArH), 4.78 (s, 1H; NacNac γ-H), 3.34–3.16 (br m, 12H; Dipp C*H*(CH_3_)_2_ and dioxane C*H*_2_), 1.63 (s, 6H; NacNac NCC*H*_3_), 1.38 (d, ^3^*J*_H–H_ = 6.9 Hz, 6H; Dipp CH(C*H*_3_)_2_), 1.24–1.17 (m, 14H*; Dipp CH(C*H*_3_)_2_), 0.88 (d, ^3^*J*_H–H_ = 7.9 Hz, 6H; Dipp CH(C*H*_3_)_2_ [*should integrate as 12H; however, the fluxionality of coordinated dioxane prevents an accurate integration of this region.] ^13^C NMR (126 MHz, C_6_D_6_): δ (ppm) = 270.97 (d, ^1^*J*_C–P_ = 34.0 Hz; CP), 169.19 (NacNac N*C*CH_3_), 145.21 (Dipp ArC), 142.81 (Dipp ArC), 142.11 (Dipp ArC), 136.43 (Dipp ArC), 125.63 (Dipp ArC), 124.17 (Dipp ArC), 94.85 (NacNac γ-C), 67.26 (Dipp *C*H(CH_3_)_2_), 27.98 (NacNac NC*C*H_3_), 25.98 (dioxane), 24.67 (Dipp CH(*C*H_3_)_2_), 24.13 (Dipp CH(*C*H_3_)_2_), 18.98 (unknown impurity). ^31^P NMR (162 MHz, C_6_D_6_): δ (ppm) = 246.7*, 177.2. [*solvent-free [Mg]CP ∼ 10%.] IR: The C≡P bond stretch was calculated to be 1327 cm^–1^. It was not possible to resolve the peak in the IR spectrum as it is masked by the aromatic C–C bond stretches which also appear this region.

### [Mg(^Dipp^NacNac)(OSi^i^Pr_3_)(dioxane)] (**2**)

Anal. Calcd (%) for C_42_H_70_MgN_2_O_3_Si: C, 71.72; H, 10.03; N, 3.98. Found: 71.99; H, 10.50; N, 4.01. ^1^H NMR (500 MHz, C_6_D_6_): δ (ppm) = 7.15 (m, overlap with C_6_D_6_ prevents integration; Dipp ArH), 4.73 (s, 1H; NacNac γ-H), 3.83 (s, 6H; dioxane), 3.21 (br sept, ^3^*J*_H–H_ = 6.9 Hz, 4H; Dipp C*H*(CH_3_)_2_), 1.59 (s, 6H; NacNac NCC*H*_3_), 1.38 (d, ^3^*J*_H–H_ = 6.8 Hz, 12H; Dipp CH(C*H*_3_)_2_), 1.21 (d, ^3^*J*_H–H_ = 6.9 Hz, 12H; Dipp CH(C*H*_3_)_2_), 0.90 (two overlapping s, 18H; Si(C(H)C*H*_3_)_3_) 0.80 (m, 3H; Si(C(*H*)CH_3_)_3_). ^13^C NMR (126 MHz, C_6_D_6_): δ (ppm) = 170.08 (NacNac N*C*CH_3_), 145.28 (Dipp ArC), 142.04 (Dipp ArC), 125.72 (Dipp ArC), 124.19 (Dipp ArC), 94.91 (NacNac γ-C), 67.95 (dioxane), 31.97 (Dipp CH(*C*H_3_)_2_), 28.35 (Dipp CH(*C*H_3_)_2_), 25.31 (NacNac NC*C*H_3_), 24.43 (NacNac NC*C*H_3_), 18.96 (Si(CH(*C*H_3_)_2_)_3_), 14.65 (Si(*C*H(CH_3_)_2_)_3_).

### Synthesis of [Mg(^Dipp^NacNac)(CP)(THF-*d*_8_)] (**3**)

Generated *in situ* by addition of THF-*d*_8_ to a solution of **1** in C_6_D_6_. ^1^H NMR (500 MHz, C_6_D_6_) δ (ppm) = 7.16–7.12 (m, 6H; Dipp ArH), 4.79 (s, 1H; NacNac γ-H), 3.53 (s; THF), 3.46 (sept, ^3^*J*_H–H_ = 6.8 Hz, 4H; Dipp C*H*(CH_3_)_2_), 3.36 (s, 8H, free dioxane), 1.64 (s, 6H; NacNac NCC*H*_3_), 1.37 (d, ^3^*J*_H–H_ = 6.8 Hz, 12H; Dipp CH(C*H*_3_)_2_), 1.21 (d, ^3^*J*_H–H_ = 6.8 Hz, 12H; Dipp CH(C*H*_3_)_2_), 0.94–0.84 (m, 3H; unknown impurity). ^13^C NMR (126 MHz, C_6_D_6_) δ (ppm) = 271.14 (d, ^1^*J*_C–P_ = 33.5 Hz; CP), 168.87 (NacNac N*C*CH_3_), 145.34 (Dipp ArC), 142.95 (Dipp ArC), 125.49 (Dipp ArC), 124.04 (Dipp ArC), 94.75 (NacNac γ-C), 67.18 (free dioxane), 67.02 (THF), 28.05 (NacNac NC*C*H_3_), 25.77 (Dipp *C*H(CH_3_)_2_), 24.76 (Dipp CH(*C*H_3_)_2_), 24.61 (THF), 24.09 (Dipp CH(*C*H_3_)_2_). ^31^P NMR (162 MHz, C_6_D_6_): δ = 175.0.

### Synthesis of [Mg(^Dipp^NacNac)(CP)(IMes)] (**5**)

**1** (∼129.5 mg, 0.22 mmol) was generated *in situ*, as outlined above. To this, IMes (143.9 mg, 0.47 mmol, 2.1 equiv) was added as a solution in toluene (0.5 mL), and the resulting mixture was stirred overnight. The volatiles were removed, and the residue was extracted with pentane (5 mL). After filtration, the solution was concentrated to 1 mL and recrystallized in a single crop over several days at −35 °C. The product was isolated as a pale orange solid (134.4 mg, 0.12 mmol, 54% yield). Anal. Calcd (%) for C_72_H_89_MgN_6_P: C, 79.06; H, 8.20; N, 7.68. Found: C, 78.10; H, 8.18; N, 7.28. ^1^H NMR (400 MHz, C_6_D_6_): δ (ppm) = 7.22 (dd, ^3^*J*_H–H_ = 7.7, 1.6 Hz, 2H; Dipp ArH), 7.08 (t, ^3^*J*_H–H_ = 7.6 Hz, 2H; Dipp ArH), 7.05–6.95 (m, 2H; Dipp ArH), 6.79 (s, 4H; free IMes ArH), 6.73 (s, 2H; coord IMes ArH), 6.52 (s, 2H; coord IMes ArH), 6.44 (s, 2H; free IMes {NC*H*}_2_), 5.86 (d, ^3^*J*_H–H_ = 1.4 Hz, 1H, coord IMes C*H*), 5.61 (d, ^3^*J*_H–H_ = 1.5 Hz, 1H; coord IMes C*H*), 4.99 (s, 2H; NacNac γ-H), 4.35 (sept, ^3^*J*_H–H_ = 6.8 Hz, 2H; Dipp C*H*(CH_3_)_2_), 3.52 (sept, ^3^*J*_H–H_ = 6.9 Hz, 2H; Dipp C*H*(CH_3_)_2_), 2.20 (s, 3H; free IMes *para*-CH_3_) 2.15 (s, 6H; NacNac NCC*H*_3_), 2.10 (s, 12H; free IMes *ortho*-CH_3_), 1.94 (s, 3H; coord IMes *para*-CH_3_), 1.78 (s, 6H; coord IMes *ortho*-CH_3_), 1.67 (s, 3H; coord IMes *para*-CH_3_), 1.57 (s, 6H; IMes *ortho*-CH_3_), 1.43 (d, ^3^*J*_H–H_ = 6.8 Hz, 6H; Dipp CH(C*H*_3_)_2_), 1.22 (dd, ^3^*J*_H–H_ = 6.8, 2.0 Hz, 12H; Dipp CH(C*H*_3_)_2_), 1.15 (d, ^3^*J*_H–H_ = 6.9 Hz, 6H; Dipp CH(C*H*_3_)_2_). ^13^C NMR (126 MHz, C_6_D_6_): δ (ppm) = 218.62 (free IMes carbene *C*), 167.02 (NacNac *C*CH_3_), 163.31 (coord IMes N*C*=C(H)N), 147.54 (Dipp *ipso*-ArC), 144.57 (coord IMes *ipso*-ArC), 141.59 (Dipp *ipso*-ArC), 139.29 (coord IMes *para*-ArC), 138.84 (free IMes *ortho*-ArC), 138.73 (Dipp *ortho*-ArC), 136.91 (coord IMes *ortho*-ArC), 135.42, 135.06 (coord IMes *C*H), 130.97 (coord IMes *C*H), 128.88 (coord IMes *meta*-ArC), 128.80 (coord IMes *meta*-ArC), 128.75 (free IMes *meta*-ArC), 124.00 (Dipp ArC), 123.76 (Dipp ArC), 122.67 (Dipp ArC), 120.14 (free IMes {N*C*H}_2_), 94.70 (NacNac γ-C), 27.80 (Dipp *C*H(CH_3_)_2_), 27.35 (Dipp CH(*C*H_3_)_2_), 27.19 (Dipp *C*H(CH_3_)_2_), 24.89 (Dipp CH(*C*H_3_)_2_), 24.43 (Dipp CH(*C*H_3_)_2_), 24.37 (Dipp CH(*C*H_3_)_2_), 24.31 (coord IMes *ortho*-CH_3_), 24.14 (coord IMes *ortho*-CH_3_), 20.73 (coord IMes *para*-CH_3_), 20.67 (free IMes *para*-CH_3_), 20.52 (NacNac C*C*H_3_), 17.64 (coord IMes *para*-CH_3_), 17.45 (free IMes *ortho-*CH_3_), 16.47 (coord IMes *para*-CH_3_) [no cyaphide carbon observed]. ^31^P{^1^H} NMR (162 MHz, C_6_D_6_): δ (ppm) = 162.9. IR ν_(CP)_ = 1316 cm^–1^ (calcd 1311 cm^–1^).

### Synthesis of [Mg(^Dipp^NacNac)(CP)(I^i^Pr)] (**6**)

**1** (∼80.2 mg, 0.14 mmol) was generated *in situ*, as described above. To this, I^*i*^Pr (21.6 mg, 0.14 mmol, 1.0 equiv) was added as a solution in toluene (0.5 mL), and the resulting solution was stirred for 2 h. The volatiles were removed, and the residue was washed with pentane (5 mL) and dried. The product was isolated as a beige solid (41.5 mg, 0.065 mmol, 48% yield). Anal. Calcd (%) for C_39_H_57_MgN_4_P: C, 73.51; H, 9.02; N, 8.79. Found: C, 72.16; H, 9.12; N, 7.59. ^1^H NMR (400 MHz, C_6_D_6_): δ (ppm) = 7.30 (d, ^3^*J*_H–H_ = 6.0 Hz, 2H; Dipp ArH), 7.19–7.13 (m, overlap with C_6_D_6_ prevents integration), 7.09 (d, ^3^*J*_H–H_ = 6.0 Hz, 2H; Dipp CH), 6.23 (s, 2H; I^*i*^Pr {NC*H*}_2_), 4.84 (s, 1H; NacNac γ-H), 4.08–3.75 (br m, 2H; Dipp C*H*(CH_3_)_2_), 3.25–3.05 (m, 1H, overlap with adjacent signal prevents total integration; I^*i*^Pr C*H*(CH_3_)_2_), 3.08–2.90 (br m, 2H; Dipp C*H*(CH_3_)_3_), 1.77 (d, ^3^*J*_H–H_ = 6.5 Hz, 2H; I^*i*^Pr CH(C*H*_3_)_2_), 1.68 (s, 6H; NacNac NCC*H*_3_), 1.34 (d, ^3^*J*_H–H_ = 6.8 Hz, 6H; Dipp CH(C*H*_3_)_2_), 1.14 (d, ^3^*J*_H–H_ = 6.8 Hz, 6H; Dipp CH(C*H*_3_)_2_), 0.73 (d, ^3^*J*_H–H_ = 6.5 Hz, 6H; I^*i*^Pr CH(C*H*_3_)_2_). ^13^C NMR (126 MHz, C_6_D_6_): δ (ppm) = 181.54 (I^i^Pr carbene *C*), 167.71 (NacNac N*C*CH_3_), 1.45.58 (Dipp *ipso*-ArC) 143.2 (Dipp *ortho*-ArC), 141.7 (Dipp *ortho*-ArC), 124.72 (Dipp *para*-ArC, overlaps with solvent), 124.14 (Dipp *meta*-ArC), 122.91 (Dipp *meta*-ArC), 116.26 (broad, I^i^Pr {N*C*H}_2_), 93.49 (NacNac γ-C) 28.42 (NacNac *C*H(CH_3_)_2_) 28.18 (NacNac NC*C*H_3_) 27.12 (broad, I^i^Pr CH(*C*H_3_)_2_), 24.94 (I^i^Pr *C*H(CH_3_)_2_) 24.80 (I^i^Pr *C*H(CH_3_)_2_), 24.55 (Dipp CH(*C*H_3_)_2_) 23.91 (NacNac NC*C*H_3_) 23.87 (Dipp CH(*C*H_3_)_2_) 23.51 (Dipp *C*H(CH_3_)_2_), 19.34 (broad, I^i^Pr CH(*C*H_3_)_2_). ^31^P{^1^H} NMR (162 MHz, C_6_D_6_): δ (ppm) = 174.9 (br s). ^31^P{^1^H} NMR (162 MHz, C_6_D_6_, 233 K): δ (ppm) = 173.3 (major) and 167.7 (minor). IR ν_(CP)_ = 1325 cm^–1^ (calcd 1312 cm^–1^).

### *In Situ* Generation of [Ge(^Dipp^NacNac)(CP)] (**7**)

**1** (∼70 mg, 0.15 mmol) was generated *in situ*, as outlined above. To this toluene solution, [Ge(^Dipp^NacNac)Cl] (39.5 mg, 0.075 mmol) was added as a solid with stirring. NMR spectroscopy showed immediate formation of a new ^31^P-containing compound. Over a few hours, the solution changed color from red to dark green, indicating decomposition. Despite efforts, no pure sample of **7** could be isolated. ^1^H NMR (400 MHz, C_6_D_6_): δ (ppm) = 5.08 (s, 1H; NacNac γ-H), 4.15 (sept, ^3^*J*_H–H_ = 6.7 Hz, 2H; Dipp {C*H*(CH_3_)_2_}), 3.42 (sept; Dipp {C*H*(CH_3_)_2_} overlapping with **2**), 1.56 (s; CC*H*_3_ overlapping with **2**), 1.44 (d, ^3^*J*_H–H_ = 6.7 Hz, 12H), 1.28 (d, ^3^*J*_H–H_ = 6.8 Hz, 12H), 1.23 (d, ^3^*J*_H–H_ = 6.9 Hz, 10H, overlap with **2** prevents precise integration), 1.07 (d, ^3^*J*_H–H_ = 6.8 Hz, 12H). Specific aromatic proton signals could not be identified due to overlap with resonance arising from **2**. ^31^P{^1^H} NMR (162 MHz, C_6_D_6_): δ (ppm) = 106.4.

### Synthesis of [Sn(^Dipp^NacNac)(CP)] (**8**)

**1** (∼91.7 mg, 0.16 mmol) was generated *in situ*, as outlined above. To this toluene solution, [Sn(^Dipp^NacNac)Cl] (82.3 mg, 0.14 mmol) was added as a solid with stirring. Over ∼2 h, the solution turned burgundy. The solvent was removed, and the residue was extracted with hexane (5 mL) and filtered. The solution was concentrated to ∼1 mL and cooled to −35 °C for 18 h to form yellow crystals of **8** (28.0 mg, 0.048 mmol, 34% yield). Anal. Calcd (%) for C_30_H_41_N_2_SnP: C, 62.19; H, 7.13; N, 4.84. Found: C, 60.87; H, 7.35; N, 4.70. ^1^H NMR (400 MHz, C_6_D_6_): δ (ppm) = 7.18–7.09 (m, overlap with residual solvent signal prevents integration), 7.07 (d, ^3^*J*_H–H_ = 2.1 Hz, 1H; Dipp CH), 7.06 (d, ^3^*J*_H–H_ = 2.1 Hz, 1H; Dipp CH), 5.04 (s, 1H; NacNac γ-*H*), 4.12 (sept, ^3^*J*_H–H_ = 6.7 Hz, 2H; Dipp C*H*(CH_3_)_2_), 3.28 (sept, ^3^*J*_H–H_ = 6.9 Hz, 2H; Dipp C*H*(CH_3_)_2_), 1.60 (s, 6H, NacNac NCC*H*_3_), 1.44 (d, ^3^*J*_H–H_ = 6.7 Hz, 6H; Dipp CH(C*H*_3_)_2_), 1.31 (d, ^3^*J*_H–H_ = 6.8, 6H; Dipp CH(C*H*_3_)_2_), 1.22 (d, ^3^*J*_H–H_ = 6.9, 6H; Dipp CH(C*H*_3_)_2_), 1.11 (d, ^3^*J*_H–H_ = 6.8 Hz, 6H; Dipp CH(C*H*_3_)_2_). ^13^C NMR (126 MHz, C_6_D_6_): δ (ppm) = 166.34 (NacNac N*C*CH_3_), 145.49 (Dipp *ortho*-*C*H), 142.65 (Dipp *ortho*-*C*H), 142.31 (Dipp *ipso*-*C*H), 136.07 (Dipp *ipso*-*C*H), 126.80 (Dipp *para*-*C*H), 124.67 (Dipp *meta*-*C*H), 123.78 (Dipp *meta*-*C*H), 100.59 (NacNac γ-*C*H), 28.89 (Dipp C(*C*H_3_)_2_), 28.74 (Dipp *C*(CH_3_)_2_) 27.76 (Dipp *C*(CH_3_)_2_) 24.51 (Dipp C(*C*H_3_)_2_) 24.15 (Dipp C(*C*H_3_)_2_) 23.64 (NacNac NC*C*H_3_) 23.18 (Dipp C(*C*H_3_)_2_). Cyaphide carbon not observed up to 350 ppm, likely due to broadening by the two adjacent nuclei. ^31^P{^1^H} NMR (162 MHz, C_6_D_6_): δ (ppm) = 122.4 (^2^*J*_P–Sn_ = 69.8 Hz, CP). ^119^Sn NMR (186 MHz, C_6_D_6_): δ = −245.6. IR ν_(CP)_ = 1321 cm^–1^ (calcd 1327 cm^–1^).

### Synthesis of [Au(IDipp)(CP)] (**9**)

**1** (∼106 mg, 0.22 mmol) was generated *in situ* as described above. This toluene solution was added dropwise to a stirred solution of [Au(IDipp)Cl] (75.0 mg, 0.12 mmol) in toluene (1 mL). The reaction was stirred for 2 h and filtered through a glass paper filter. The solvent was removed to dryness, and the yellow solids were washed with hexane (3 × 2 mL) [NB: the hexane fraction can be collected and concentrated to afford **2**]. The resulting white solid was taken into toluene (2 mL) and filtered to remove the remaining [Mg(^Dipp^NacNac)Cl(dioxane)]. Crystals were obtained by slow diffusion of hexane into a concentrated toluene solution at −35 °C (41 mg, 54% yield). Anal. Calcd (%) for C_28_H_36_AuN_2_P: C, 53.51; H, 5.77; N, 4.46. Found: 54.14; H, 5.87; N, 4.32. ^1^H NMR (500 MHz, C_6_D_6_): δ (ppm) = 7.15 (m, 2H; Dipp *para*-C*H*), 7.01 (d, ^3^*J*_H–H_ = 7.7 Hz, 4H; Dipp *meta-*C*H*), 6.21 (s, 2H; {NC*H*}_2_), 2.49 (sept, ^3^*J*_H–H_ = 6.9 Hz, 4H; Dipp {C*H*(CH_3_)_2_}), 1.40 (d, ^3^*J*_H–H_ = 6.9 Hz, 12H; Dipp {CH(C*H*_3_)_2_}), 1.04 (d, 12H, ^3^*J*_H–H_ = 6.9 Hz; Dipp {CH(C*H*_3_)_2_}), 0.30 (s, < 1H; unknown impurity). ^13^C NMR (126 MHz, C_6_D_6_): δ (ppm) = 247.70 (d, ^1^*J*_C–P_ = 6.1 Hz; CP), 193.02 (d, ^3^*J*_C–P_ = 5.0 Hz; carbene C), 145.29 ({N*C*H}_2_), 144.72 (ArC), 134.02 (ArC), 130.49 (ArC), 123.94 (ArC), 122.57 (ArC), 28.65 (Dipp {*C*H(CH_3_)_2_}), 24.45 (Dipp {CH(*C*H_3_)_2_}), 23.53 (Dipp {CH(*C*H_3_)_2_}). ^31^P{^1^H} NMR (162 MHz, C_6_D_6_): δ (ppm) = 84.1. IR ν_(CP)_ = 1342 cm^–1^ (calcd 1332 cm^–1^).

### Synthesis of [Co(^Dipp^PDI)(CP)] (**10**)

**1** (∼65 mg, 0.11 mmol) was generated *in situ* as described above. To this toluene solution was added [Co(^Dipp^PDI)Cl] (33 mg, 0.06 mmol) as a solid with stirring. The reaction was stirred for 4 days. The solvent was removed, and the residue was washed with hexane (2 × 5 mL) and then extracted with toluene (1.5 mL). After filtration, the resulting deep blue solution was concentrated and stored at −35 °C for 1 week to form blue crystals of **10**. The supernatant solution was concentrated and stored at −35 °C to yield a second crop of crystals (17 mg, 0.03 mmol, 51% yield). ^1^H NMR (400 MHz, C_6_D_6_): δ (ppm) = 9.76 (t, ^3^*J*_H–H_ = 7.7 Hz, 1H; pyridine *para*-C*H*), 7.51 (t, ^3^*J*_H–H_ = 7.7 Hz, 2H; Dipp *para*-C*H*), 7.36 (d, ^3^*J*_H–H_ = 7.7 Hz, 4H; Dipp *meta*-C*H*), 7.03 (d, ^3^*J*_H–H_ = 7.7 Hz, 2H; pyridine *meta*-C*H*), 3.10 (sept, ^3^*J*_H–H_ = 6.8 Hz, 4H; Dipp {C*H*(CH_3_)_2_}), 1.21 (d, ^3^*J*_H–H_ = 6.8 Hz, 11H; Dipp {CH(C*H*_3_)_2_}), 1.04 (d, ^3^*J*_H–H_ = 6.8 Hz, 12H; Dipp {CH(C*H*_3_)_2_}), −0.23 (s, 6H; NCC*H*_3_). ^13^C NMR (151 MHz, C_6_D_6_) δ 169.06 (imine N*C*CH_3_), 155.03 (Dipp *ipso*-Ar*C*), 153.78 (Dipp *ortho*-Ar*C*), 140.95 (Dipp *para*-ArC), 123.75 (Dipp *meta*-Ar*C*), 123.60 (pyridine *meta*-Ar*C*), 120.07 (pyridine *para*-Ar*C*), 28.96 (Dipp {*C*H(CH_3_)_2_}), 24.11 (Dipp {CH(*C*H_3_)(CH_3_)}), 23.75 (Dipp {CH(CH_3_)(*C*H_3_)}), 22.22 (NC*C*H_3_). ^31^P{^1^H} NMR (162 MHz, C_6_D_6_): δ (ppm) = 345.4. IR ν_(CP)_ = 1306 cm^–1^ (calcd 1283 cm^–1^).

## Accession Codes

CCDC 2078056–2078062 and 2086971 contain the supplementary crystallographic data for this paper. These data can be obtained free of charge via www.ccdc.cam.ac.uk/data_request/cif, or by emailing data_request@ccdc.cam.ac.uk, or by contacting The Cambridge Crystallographic Data Centre, 12 Union Road, Cambridge CB2 1EZ, UK; fax: +44 1223 336033.
