# Supplementary material for: A Cyaphide Transfer Reagent
Source: J Am Chem Soc. 2021 Jun 30;143(27):10367–73. doi: 10.1021/jacs.1c04417 (PMC8297854; doi:10.1021/jacs.1c04417)
Supplement: Supplementary file 1 — ja1c04417_si_001.pdf [file ja1c04417_si_001.pdf]

Supplementary Information

A Cyaphide Transfer Reagent

Daniel W. N. Wilson, Stephanie J. Urwin, Eric S. Yang and Jose M. Goicoechea*

Department of Chemistry, University of Oxford, Chemistry Research Laboratory, 12 Mansfield Road, Oxford, OX1 3TA, U.K.

E-mail: jose.goicoechea@chem.ox.ac.uk

Contents

1. NMR spectra for all compounds 3

1.1. NMR spectra for an *in situ* generated 1:1 mixture of [Mg(^{Dipp}NacNac)(CP)(dioxane)] (**1**) and [Mg(^{Dipp}NacNac)(OSi^{i}Pr$_3$)(dioxane)] (**2**) 3

1.2. NMR spectra for [(^{Dipp}NacNac)Mg(CP)(dioxane)] (**1**) 5

1.3. NMR spectra for [(^{Dipp}NacNac)Mg(OSi^{i}Pr$_3$)(dioxane)] (**2**) 7

1.4. NMR spectra for [Mg(^{Dipp}NacNac)(CP)(THF-d$_8$)] (**3**) 8

1.5. Stability of [Mg(^{Dipp}NacNac)(CP)(dioxane)] (**1**) and [Mg(^{Dipp}NacNac)(CP)(THF-d$_8$)] (**3**) 10

1.6. NMR spectra for [Mg(^{Dipp}NacNac)(CP)(IMes)] (**5**) 12

1.7. NMR spectra for [Mg(^{Dipp}NacNac)(CP)(I^{i}Pr)] (**6**) 14

1.8. NMR spectra for the *in situ* formation of [Ge(^{Dipp}NacNac)(CP)] (**7**) 16

1.9. NMR spectra for [Sn(^{Dipp}NacNac)(CP)] (**8**) 17

1.10. NMR spectra for [Au(IDipp)(CP)] (**9**) 19

1.11. NMR spectra for [(^{Dipp}PDI)Co(CP)] (**10**) 20

1.12. NMR spectra for [Mg(^{Dipp}NacNac)Cl] 22

2. ATR-IR spectra of cyaphide complexes 22

3. Single crystal X-ray diffraction data 25

4. Computational details 30

3.1 General computational methods 30

3.2 Calculated energies and mechanisms 31

3.3. Calculated C≡P stretching frequencies 33

3.4. Calculated ^{31}P NMR shifts 33

3.5. XYZ coordinates 34

5. References 100

1. NMR spectra for all compounds

1.1. NMR spectra for an *in situ* generated 1:1 mixture of [Mg(^{Dipp}NacNac)(CP)(dioxane)] (**1**) and [Mg(^{Dipp}NacNac)(OSi^{i}Pr$_3$)(dioxane)] (**2**)

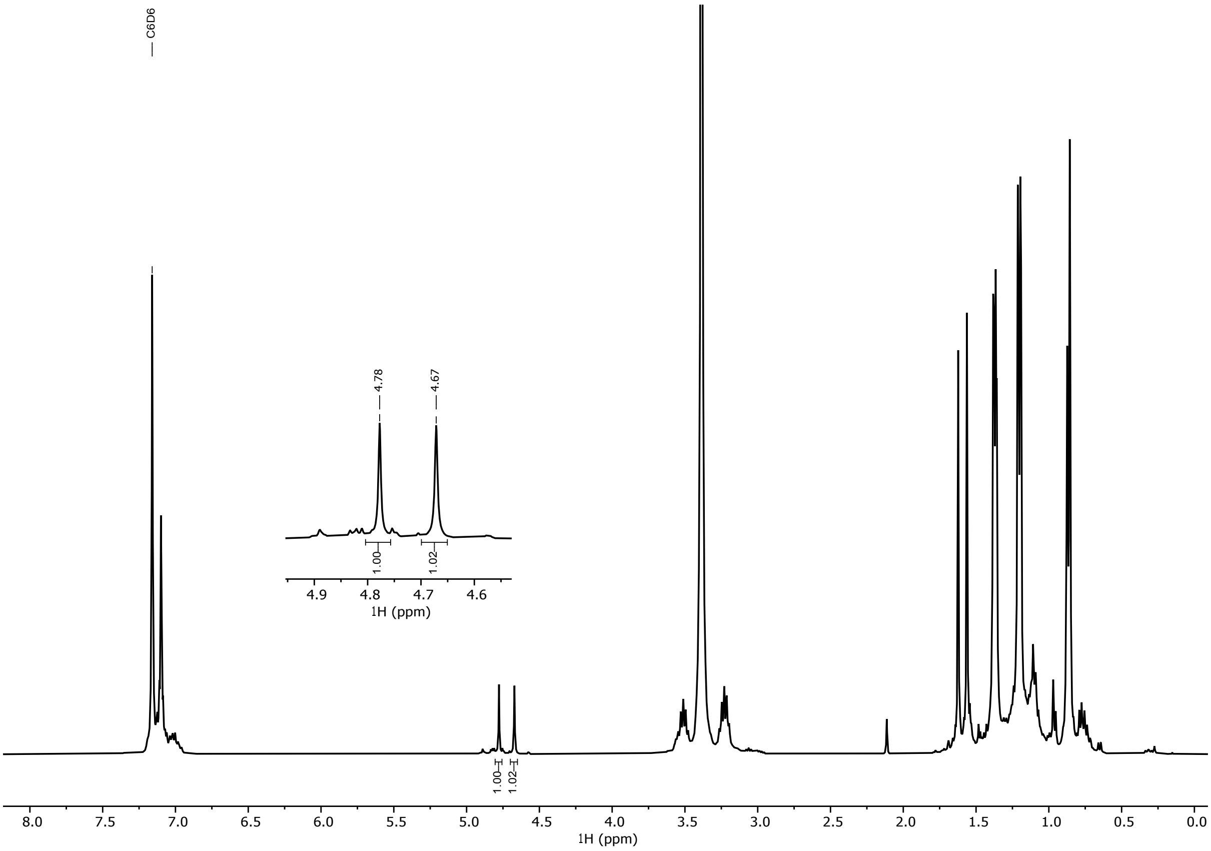

Figure S1. Representative ^{1}H NMR spectrum (400 MHz, 295 K) for the *in situ* generation of **1** and **2** in C_6D_6.

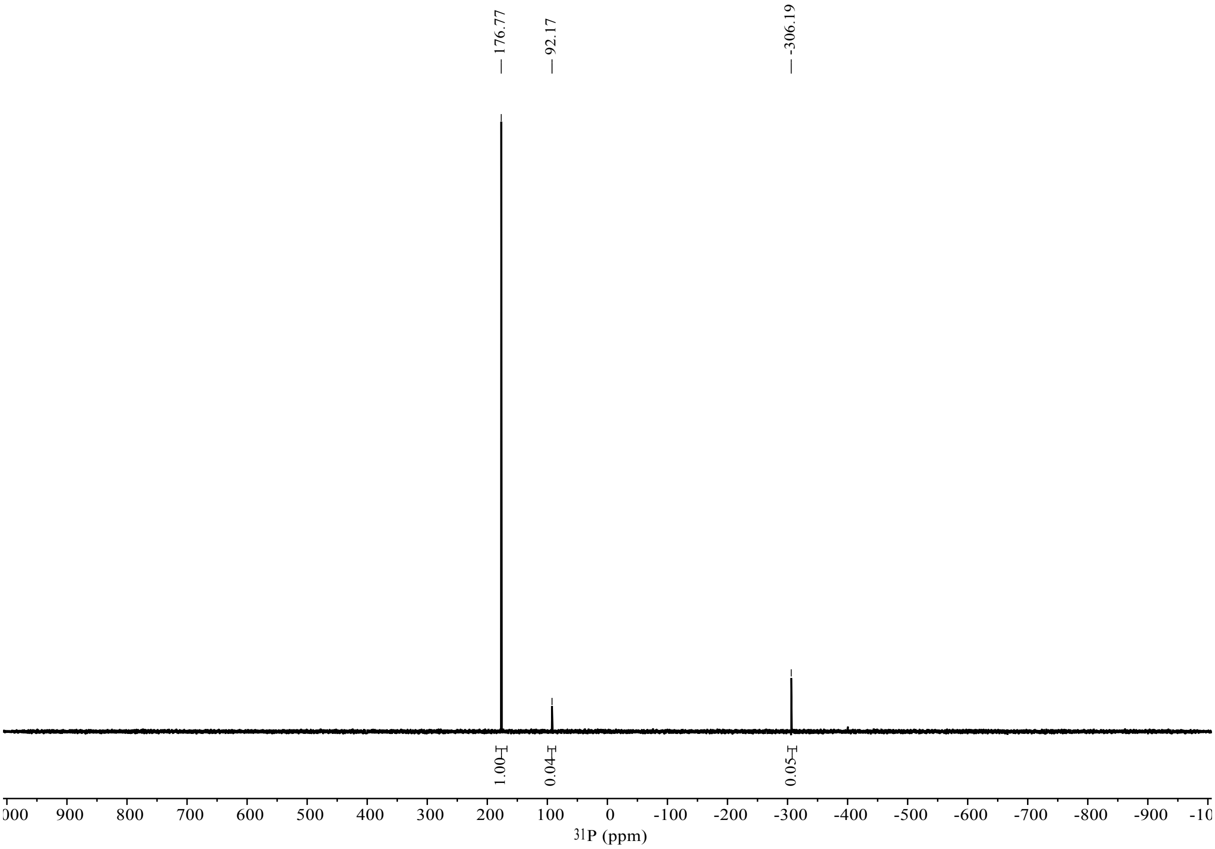

Figure S2. Representative $^{31}P\{^1H\}$ NMR spectrum (162 MHz, 295 K) for the *in situ* generation of **1** and **2** in C_6D_6. Resonance at −306.2 ppm is $^iPr_3SiOCP$ (< 5%), unidentified impurity at 92.2 ppm.

1.2. NMR spectra for [(^{Dipp}NacNac)Mg(CP)(dioxane)] (**1**)

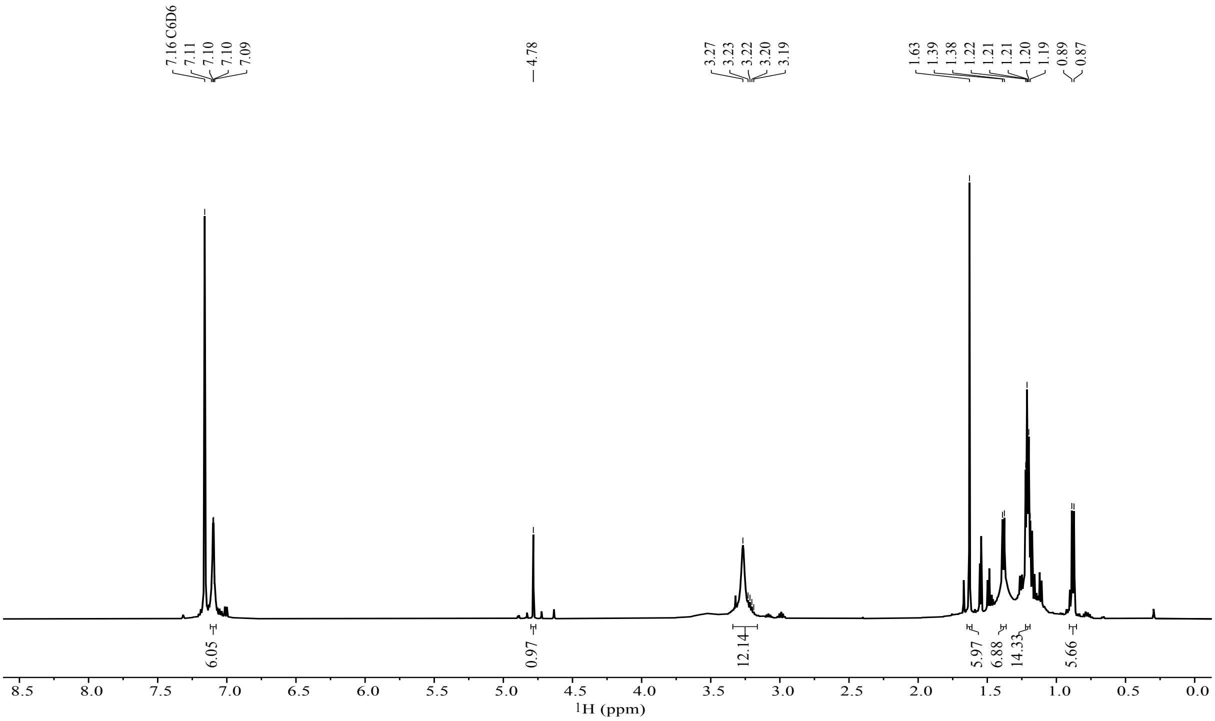

Figure S3. ^{1}H NMR spectrum (500 MHz, 295 K) of **1** in C_6D_6 from a crystalline sample.

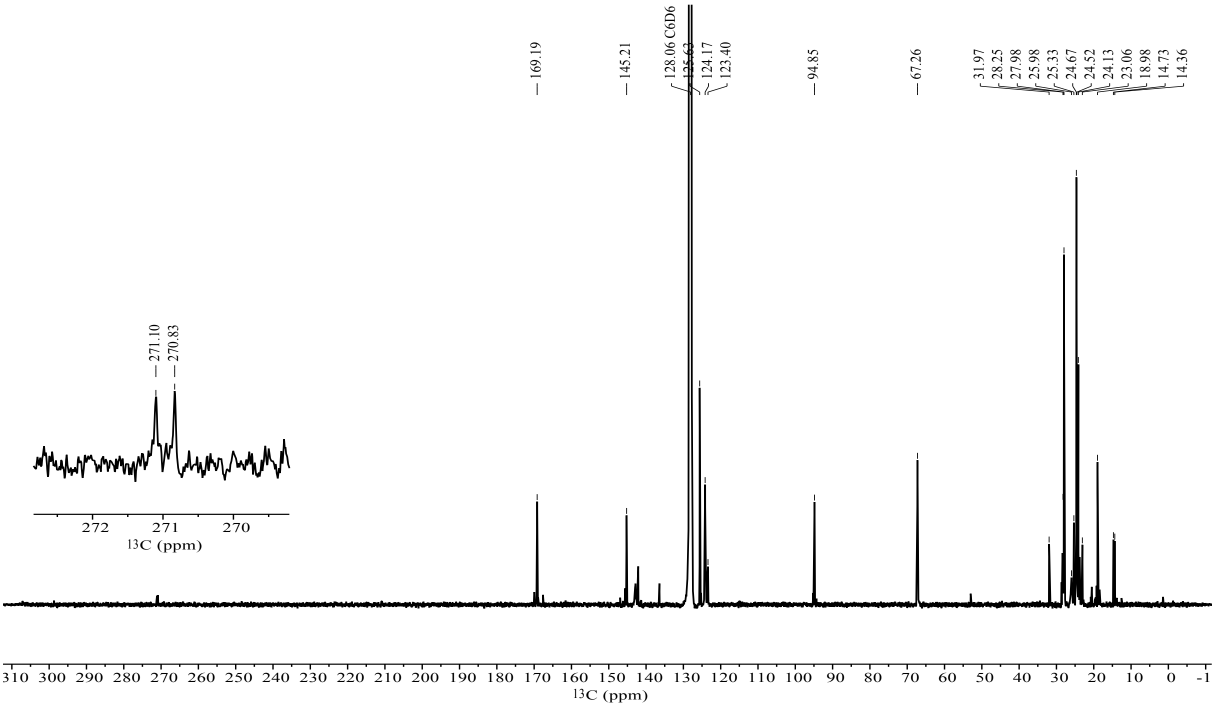

Figure S4. $^{13}C\{^{1}H\}$ NMR spectrum (126 MHz, 295 K) of **1** in C_6D_6 from a crystalline sample.

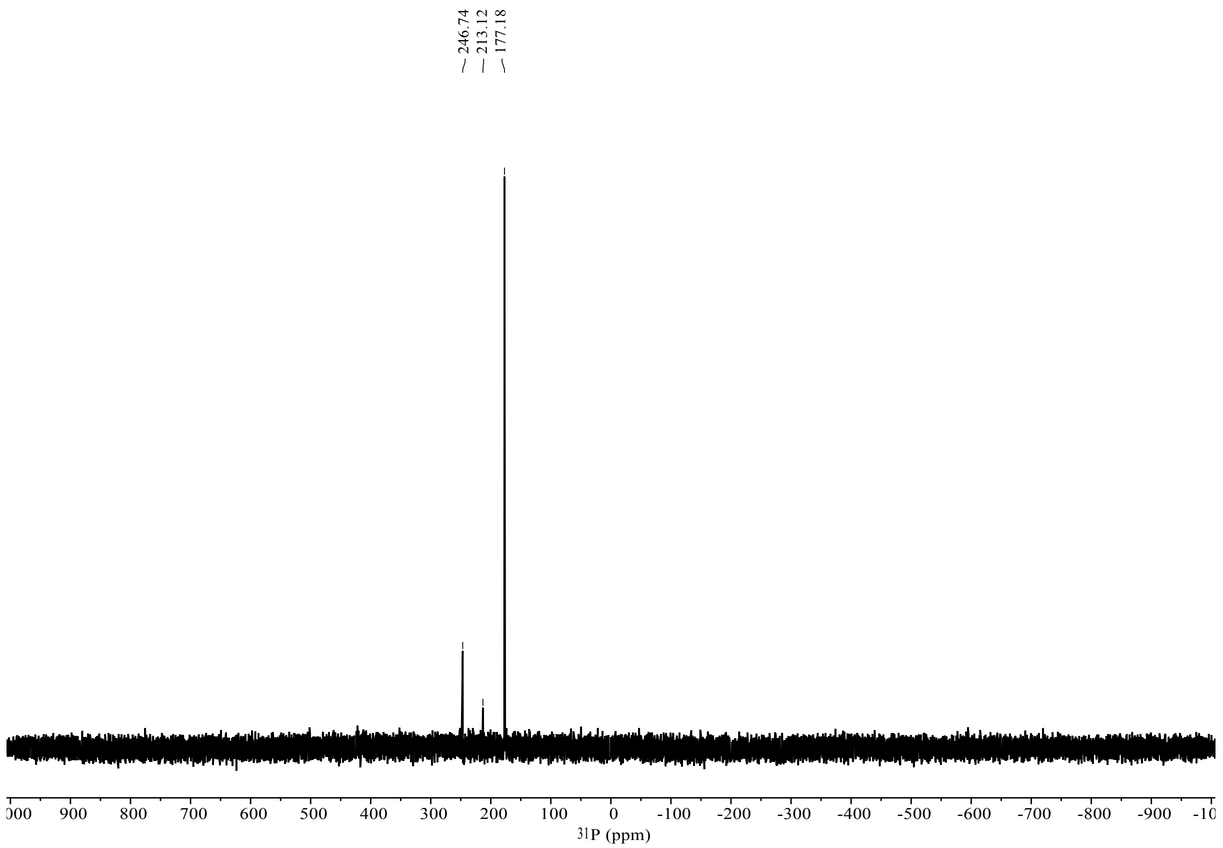

Figure S5. $^{31}P\{^{1}H\}$ NMR spectrum (162 MHz, 295 K) of **1** in C_6D_6 from crystalline sample. Minor resonances at 213.1 and 246.7 ppm correspond to impurities, the latter of which has been tentatively assigned as [Mg(^{Dipp}NacNac)(CP)]$_x$ (**4**)

1.3. NMR spectra for [(^{Dipp}NacNac)Mg(OSi^{i}Pr$_3$)(dioxane)] (**2**)

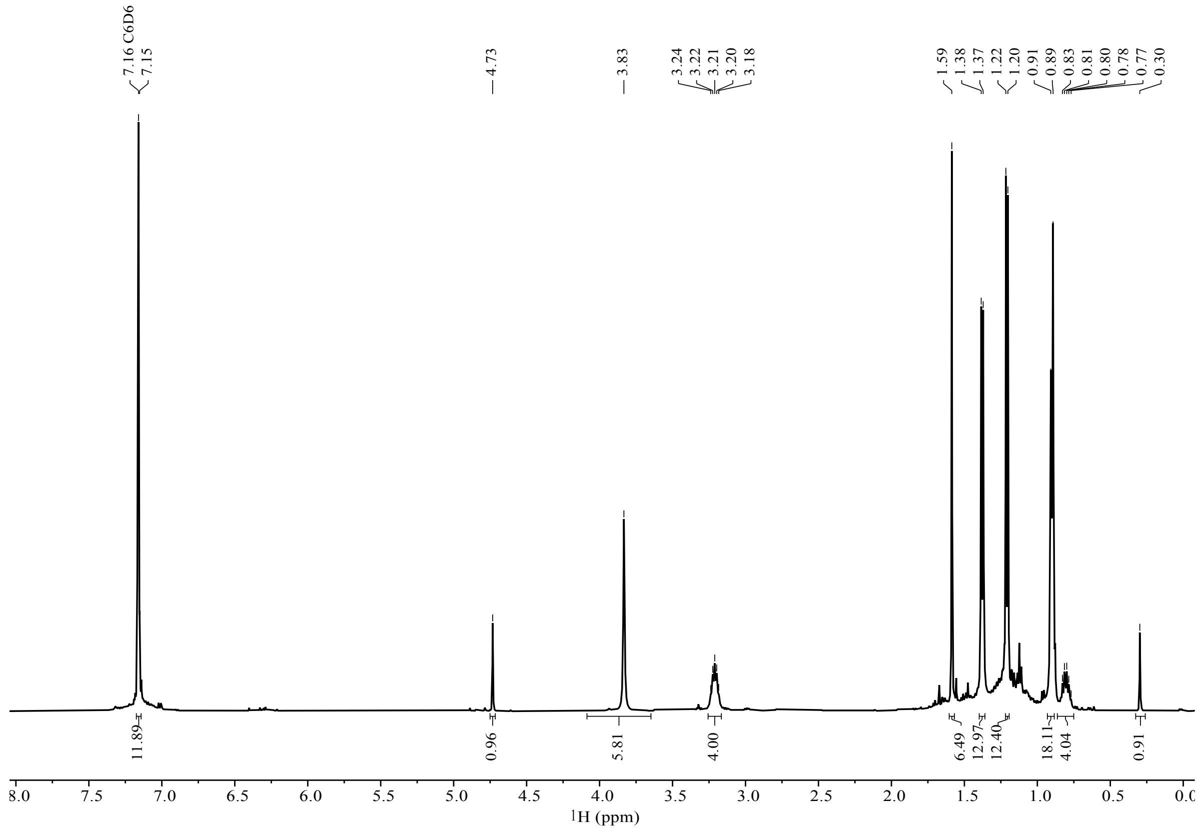

Figure S6. ^{1}H NMR spectrum (500 MHz, 295 K) of **2** in C_6D_6.

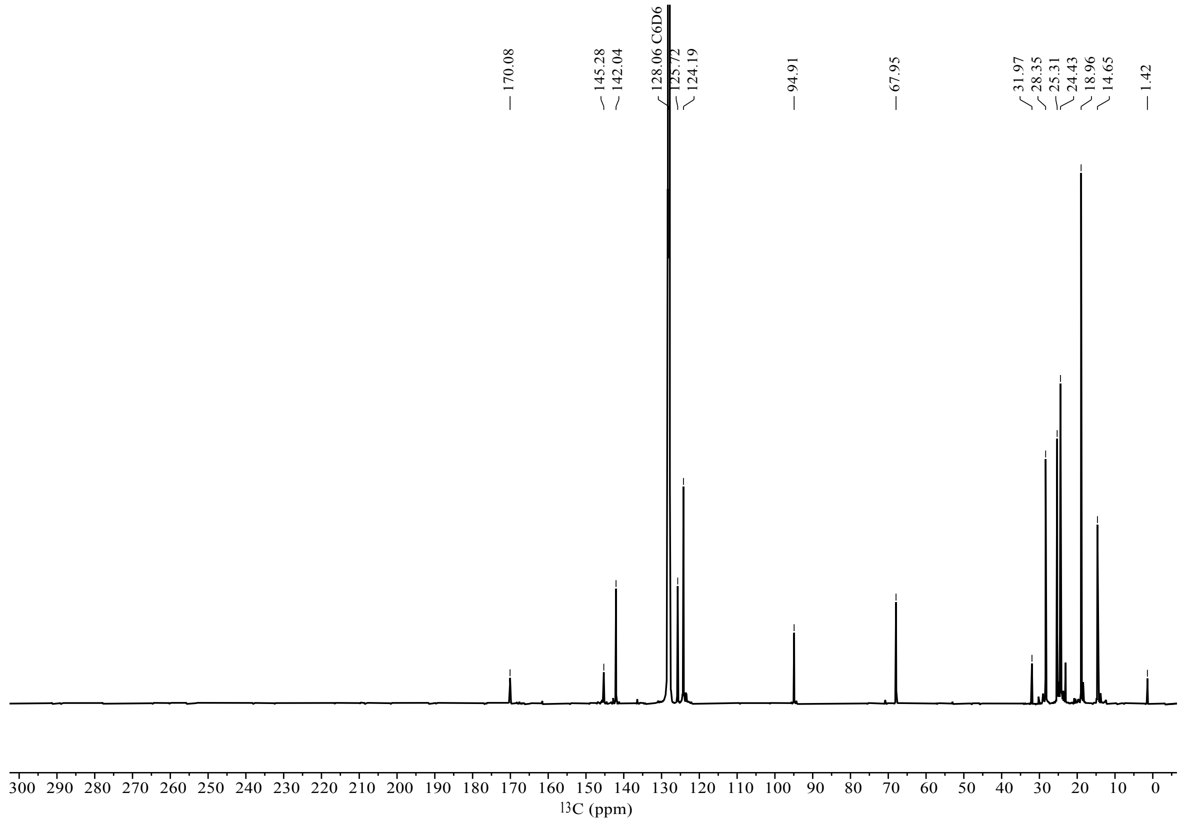

Figure S7. $^{13}C\{^{1}H\}$ NMR spectrum (126 MHz, 295 K) of **2** in C_6D_6.

1.4. NMR spectra for [Mg(^{Dipp}NacNac)(CP)(THF-d_8)] (3)

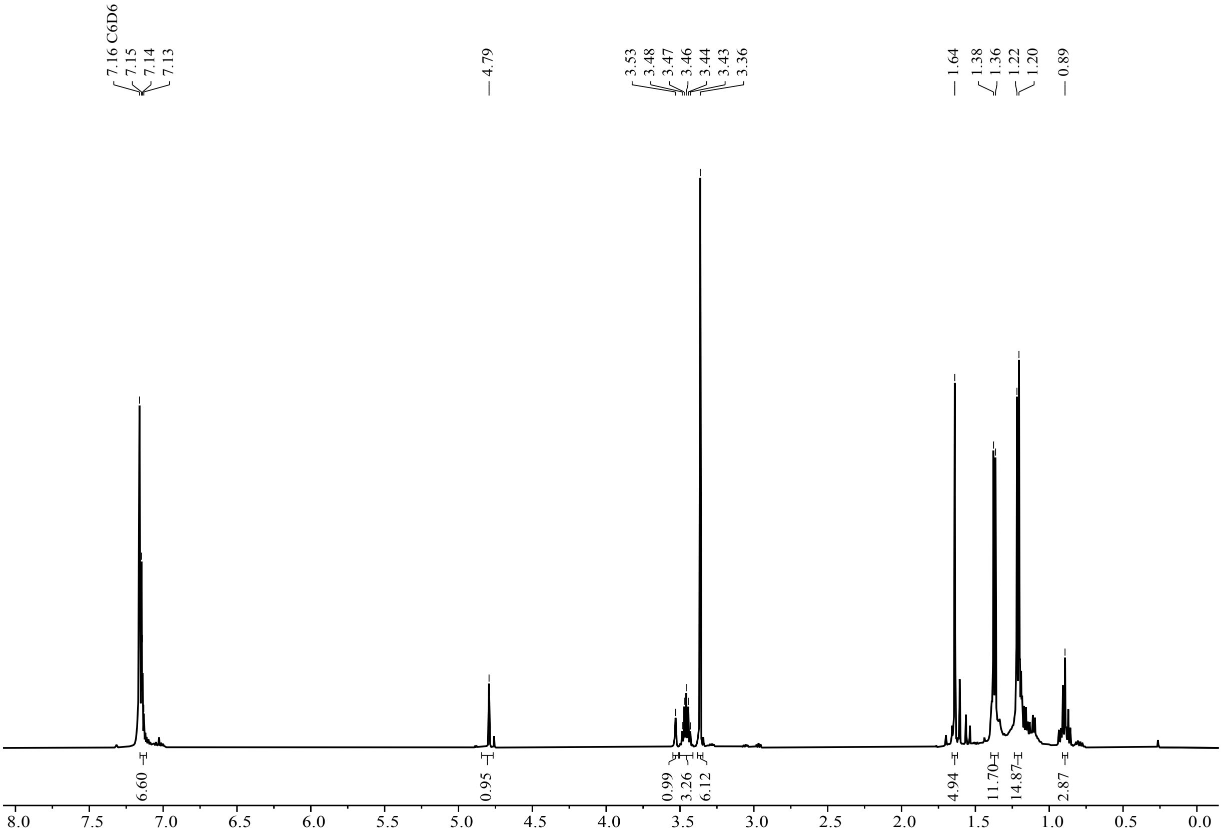

Figure S8. ^{1}H NMR spectrum (500 MHz, 295 K) of **3** in C_6D_6, obtained from the addition of THF-d_8 to a sample of **1**.

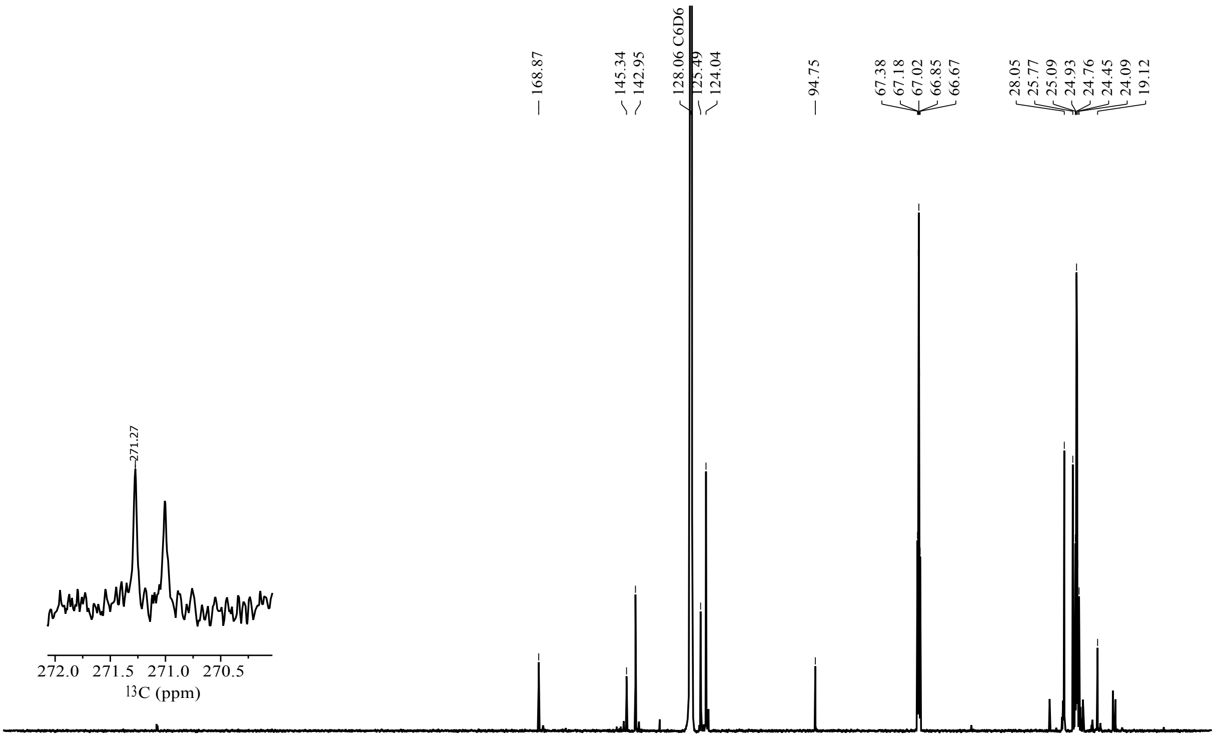

Figure S9. $^{13}C\{^{1}H\}$ NMR spectrum (126 MHz, 295 K) of **3** in C_6D_6, obtained from addition of THF-d_8 to a sample of **1**.

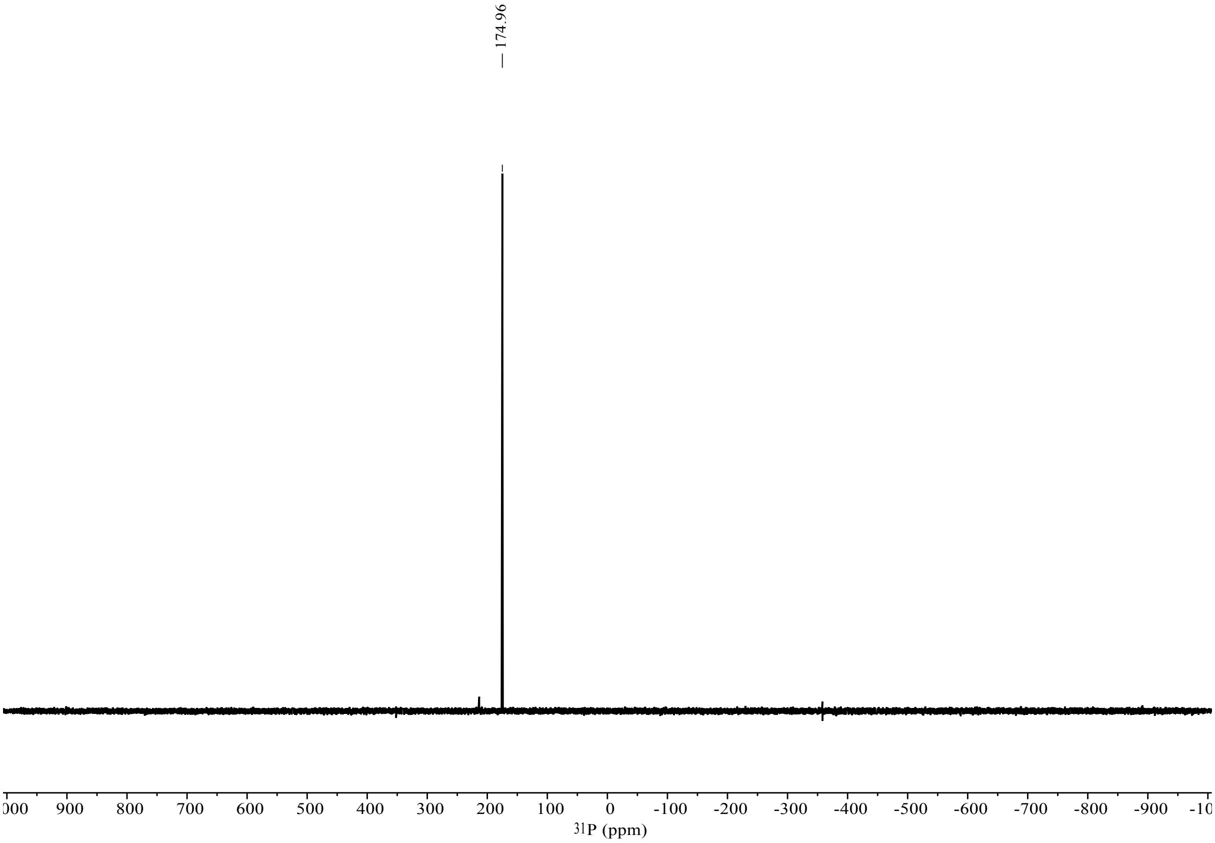

Figure S10. $^{31}P\{^{1}H\}$ NMR spectrum (162 MHz, 295 K) of **3** in C_6D_6, obtained from addition of THF-d_8 to a sample of **1**.

1.5. Stability of [Mg(^{Dipp}NacNac)(CP)(dioxane)] (**1**) and [Mg(^{Dipp}NacNac)(CP)(THF-d_8)] (**3**)

Both solvated magnesium cyaphide complexes are highly sensitive, showing significant decomposition upon standing in solution for prolonged periods, with a lifespan of days in toluene or hours in THF. Exposure of these complexes to vacuum also results in decomposition, presumably from desolvation of the magnesium centre resulting in a highly reactive species. Crystals of [Mg(^{Dipp}NacNac)(CP)(THF-d_8)] (**3**), generated from the addition of THF-d_8 to a sample of [Mg(^{Dipp}NacNac)(CP)(dioxane)] (**1**) followed by crystallisation from hexane, were dissolved in C_6D_6. The ^{1}H NMR spectrum displayed a single resonance corresponding to the β-diketiminate methine proton (Figure S11; bottom). The solvent was evaporated to dryness, and the yellow solid dissolved in C_6D_6. The new ^{1}H spectra displays multiple new resonances and a broadening of the resonance corresponding to **3** (Figure S11, middle). Repeating this process once more further broadens the spectrum (Figure S11, top). Monitoring this process by ^{31}P NMR spectroscopy shows loss of intensity after each cycle, in addition to the appearance of the resonance at 246 ppm, previously observed in crystalline samples of **1** (Figure S12). We believe this higher frequency resonance corresponds to the solvent-free species [Mg(^{Dipp}NacNac)(CP)]$_x$ (**4**). The lighter cyanide analogue, [Mg]CN, exists as a trimer in solution, [Mg(^{Dipp}NacNac)(CN)]$_3$.[1] Addition of THF to the reaction mixture results in conversion back to the single peak corresponding to **3**.

In an attempt to isolate the solvent-free species **4**, the synthesis of the complex was repeated with dioxane-free NaPCO. The ^{31}P NMR displayed a singlet resonance at 246 ppm, consistent with the species observed in the stability experiments. Addition of THF-d_8 to the reaction mixture induced complete conversion to **3**, as observed by NMR spectroscopy. Crystals of the solvent-free system were obtained by layering a toluene solution with hexane. However, the

crystals were highly sensitive and decomposed when attempting to mount them on the diffractometer.

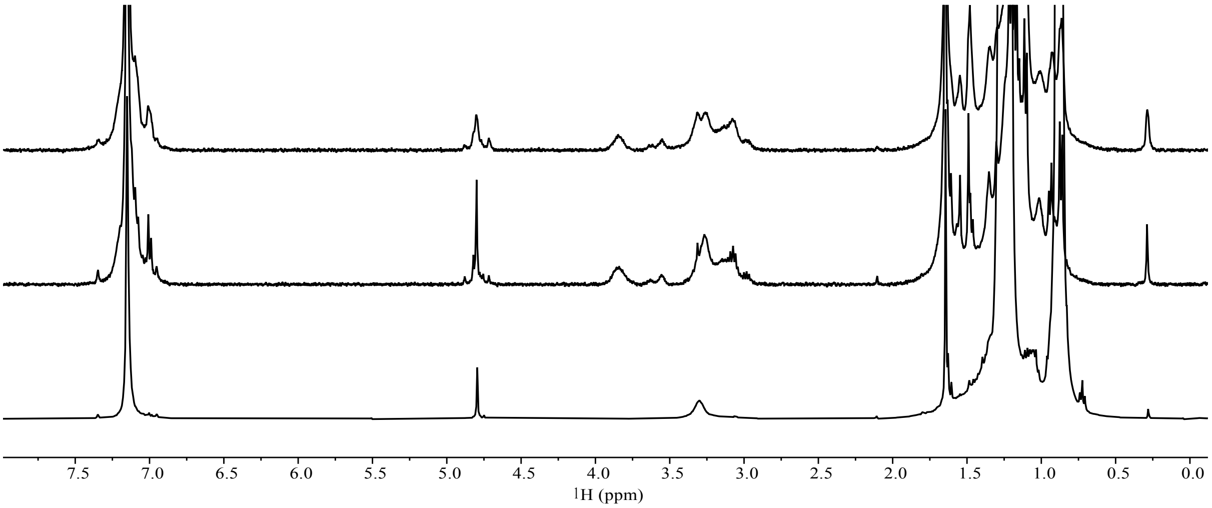

Figure S6. ^{1}H NMR spectra (400 MHz, 295 K) displaying the stability of **3** to vacuum, showing decomposition after each cycle.

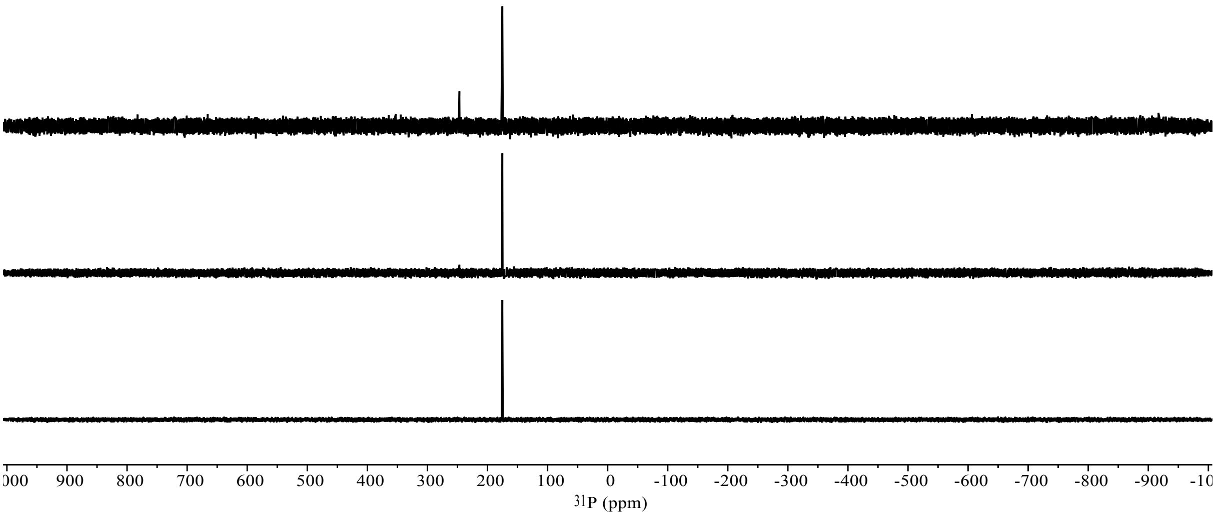

Figure S7. ^{31}P NMR spectra (162 MHz, 295 K) showing the stability of **3** to vacuum, showing decomposition after each cycle.

1.6. NMR spectra for [Mg(^{Dipp}NacNac)(CP)(IMes)] (**5**)

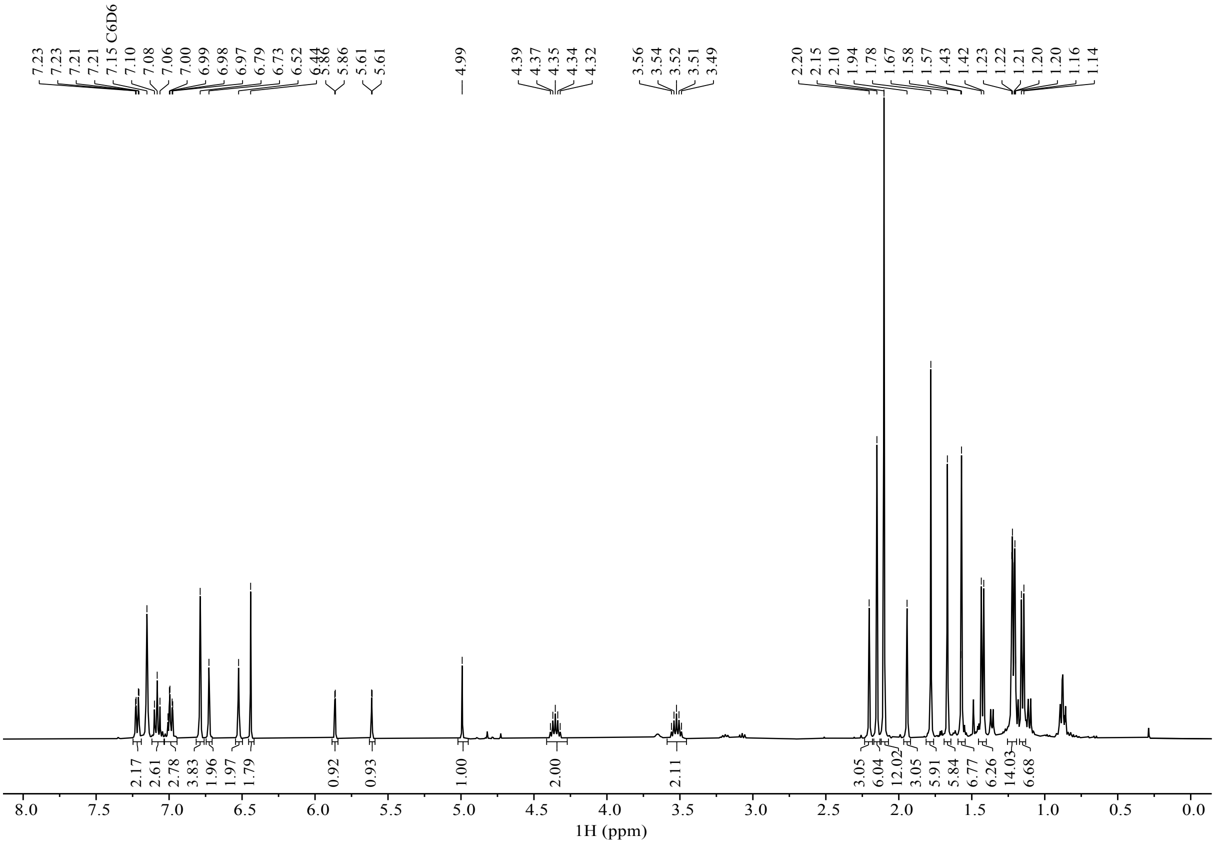

Figure S8. ^{1}H NMR spectrum (400 MHz, 295 K) of **5** in C_6D_6.

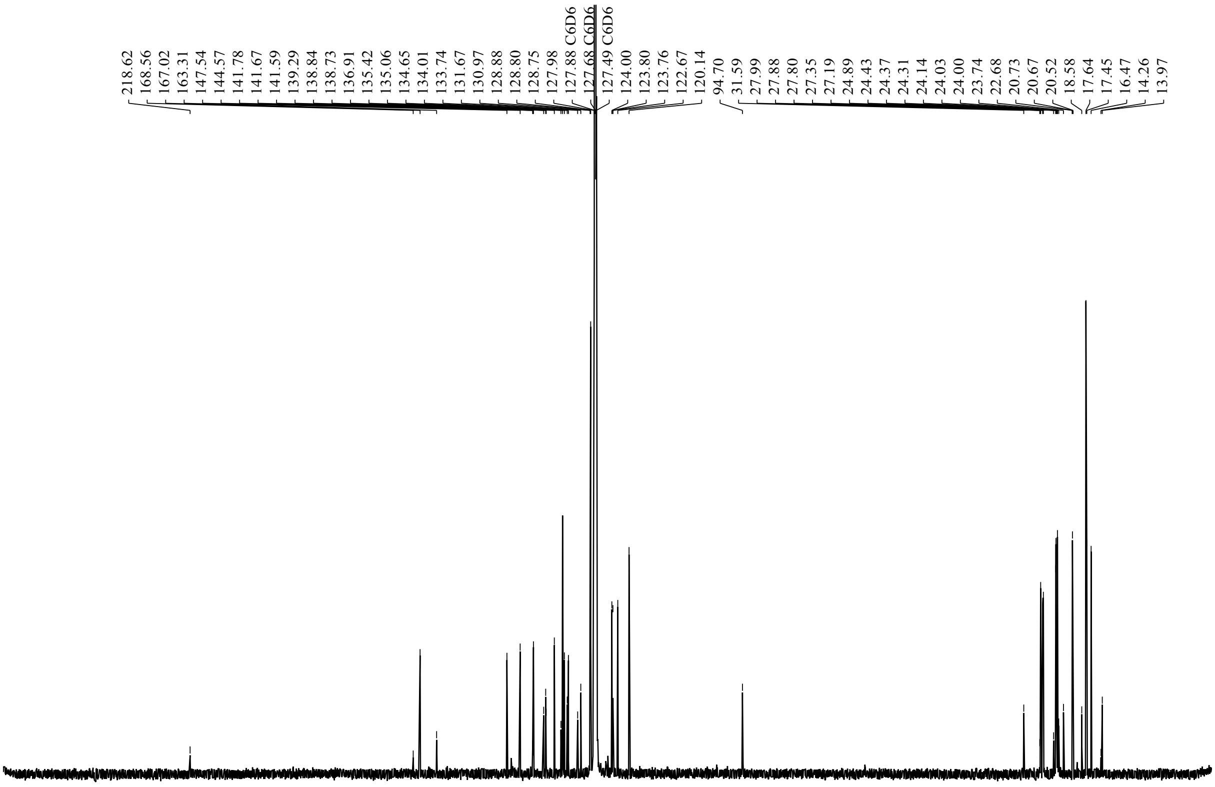

Figure S9. ^{13}C NMR spectrum (126 MHz, 295 K) of **5** in C_6D_6.

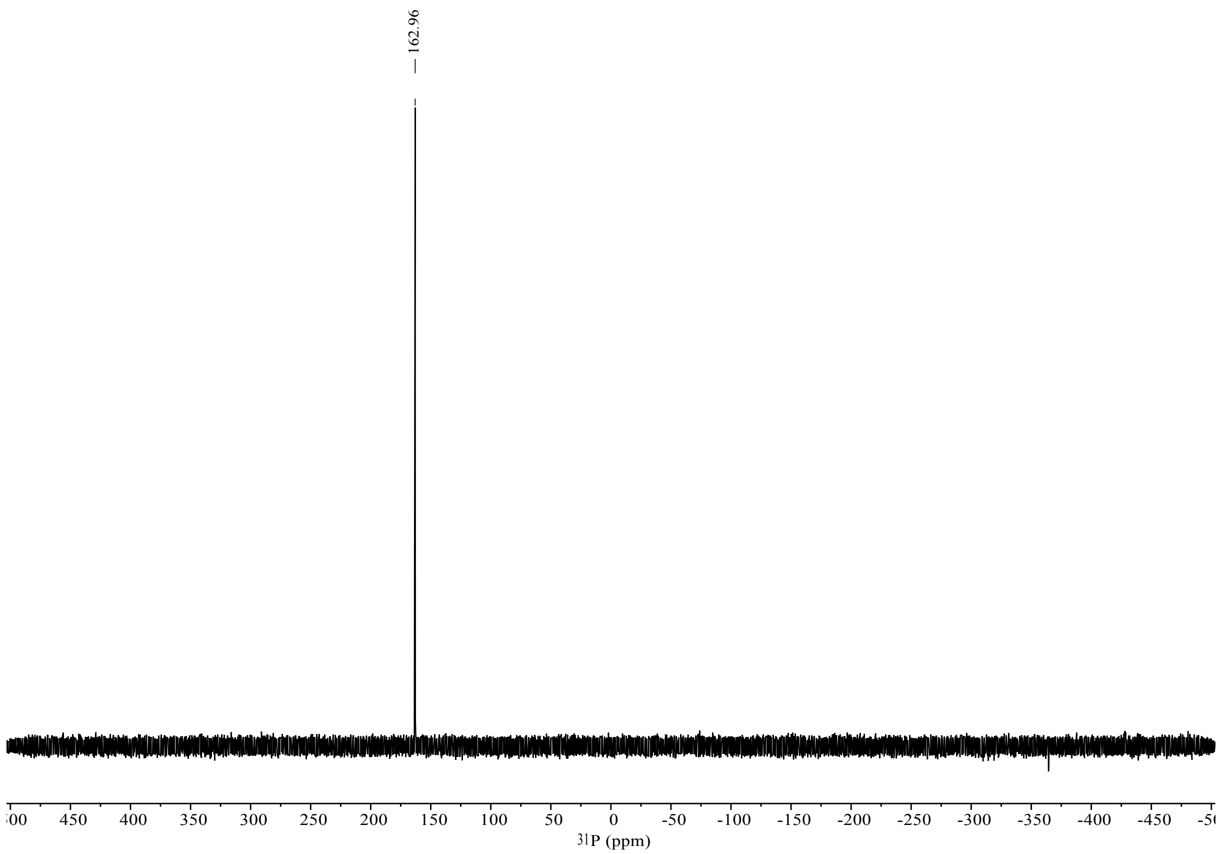

Figure S10. ^{31}P NMR spectrum (162 MHz, 295 K) of **5** in C_6D_6.

1.7. NMR spectra for [Mg(^{Dipp}NacNac)(CP)(I^{i}Pr)] (**6**)

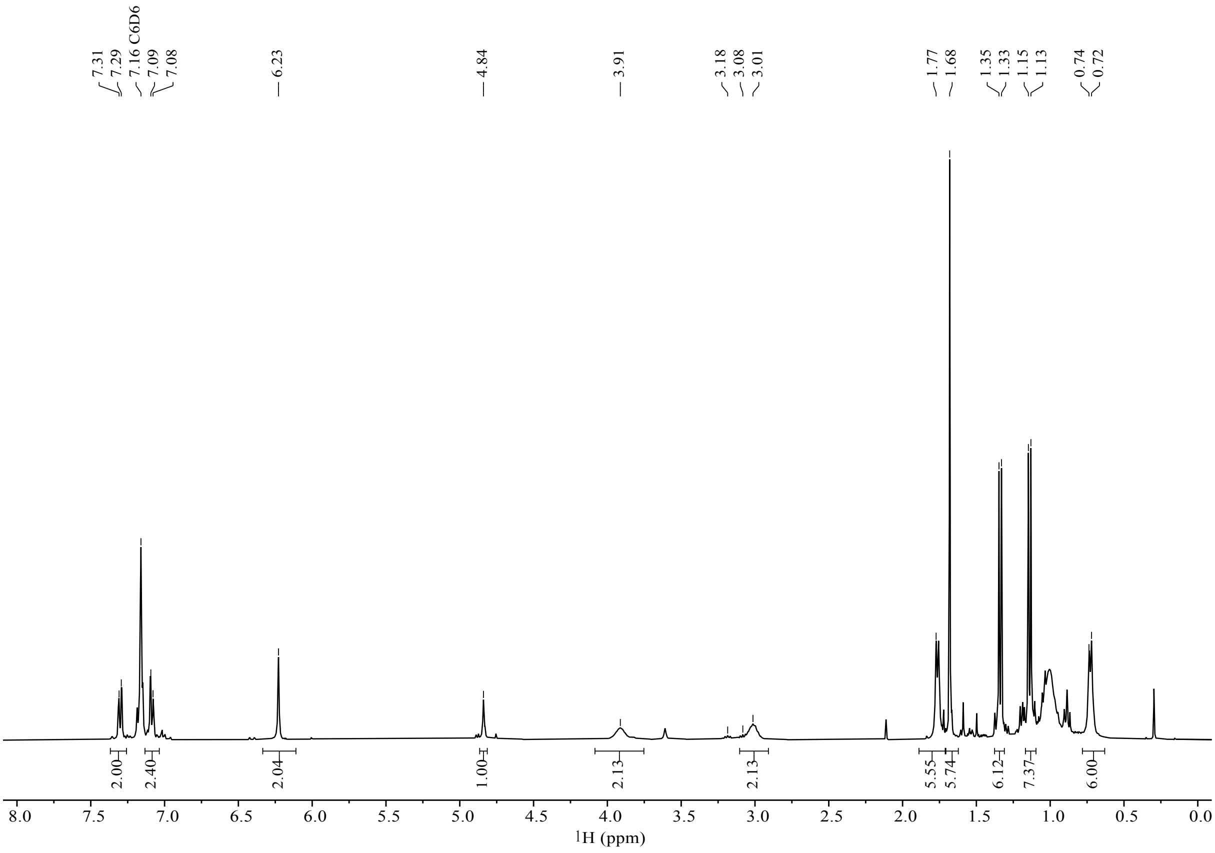

Figure S11. ^{1}H NMR spectrum (400 MHz, 295 K) of **6** in C_6D_6.

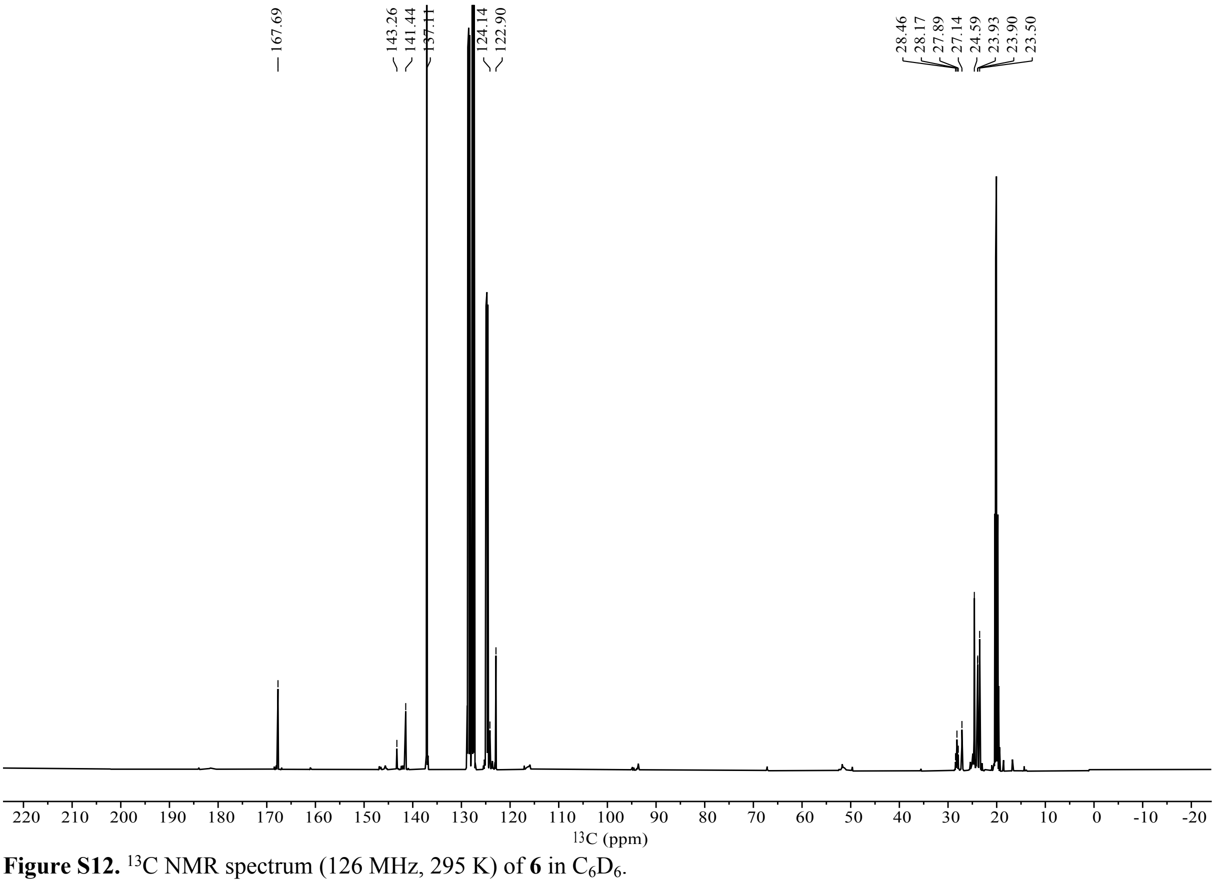

Figure S12. ^{13}C NMR spectrum (126 MHz, 295 K) of **6** in C_6D_6.

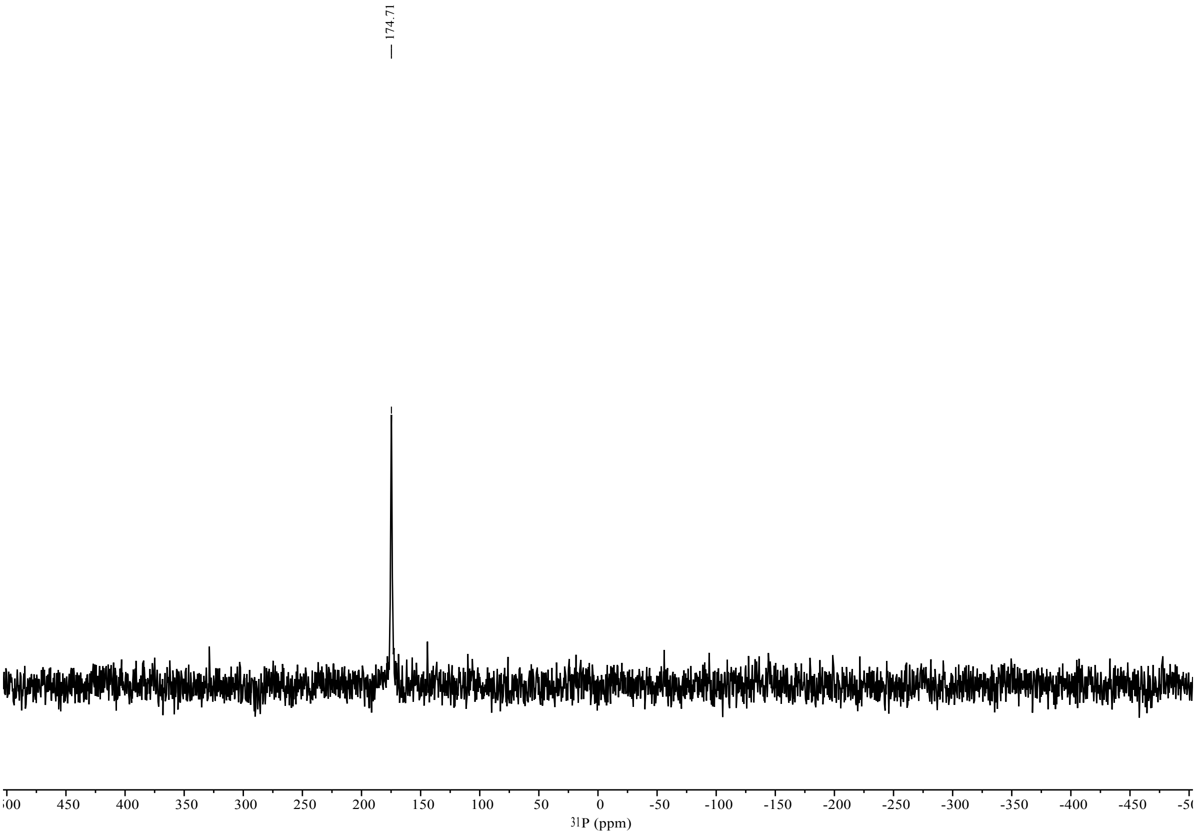

Figure S13. ^{31}P NMR spectrum (162 MHz, 295 K) of **6** in C_6D_6.

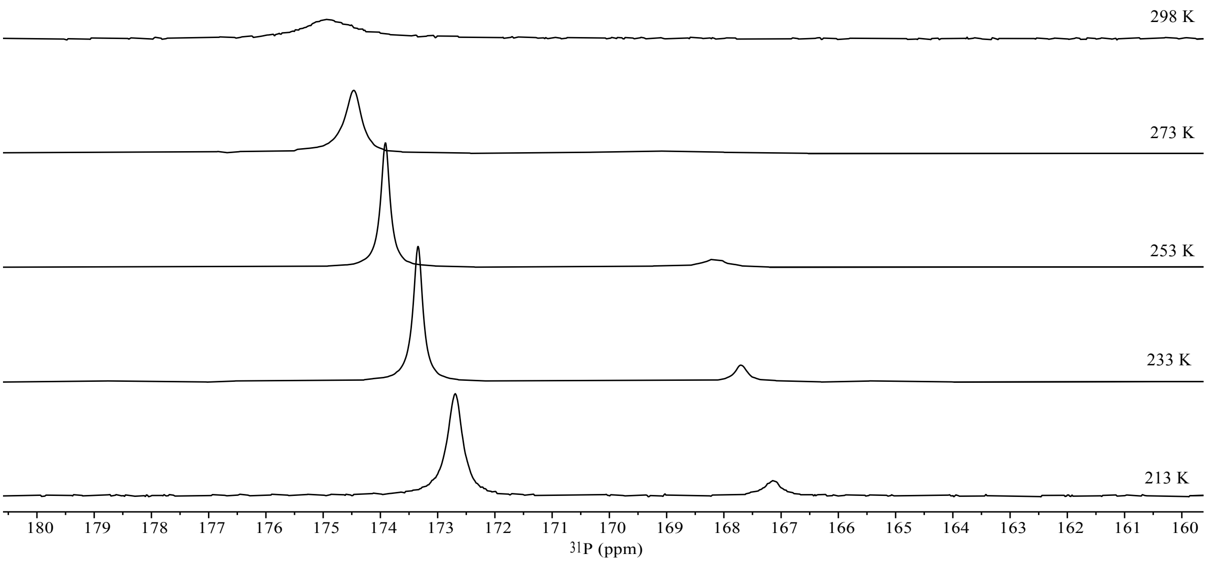

Figure S19. Variable temperature ^{31}P NMR spectrum (126 MHz) of **6** in d^8-toluene.

1.8. NMR spectra for the *in situ* formation of [Ge(^{Dipp}NacNac)(CP)] (7)

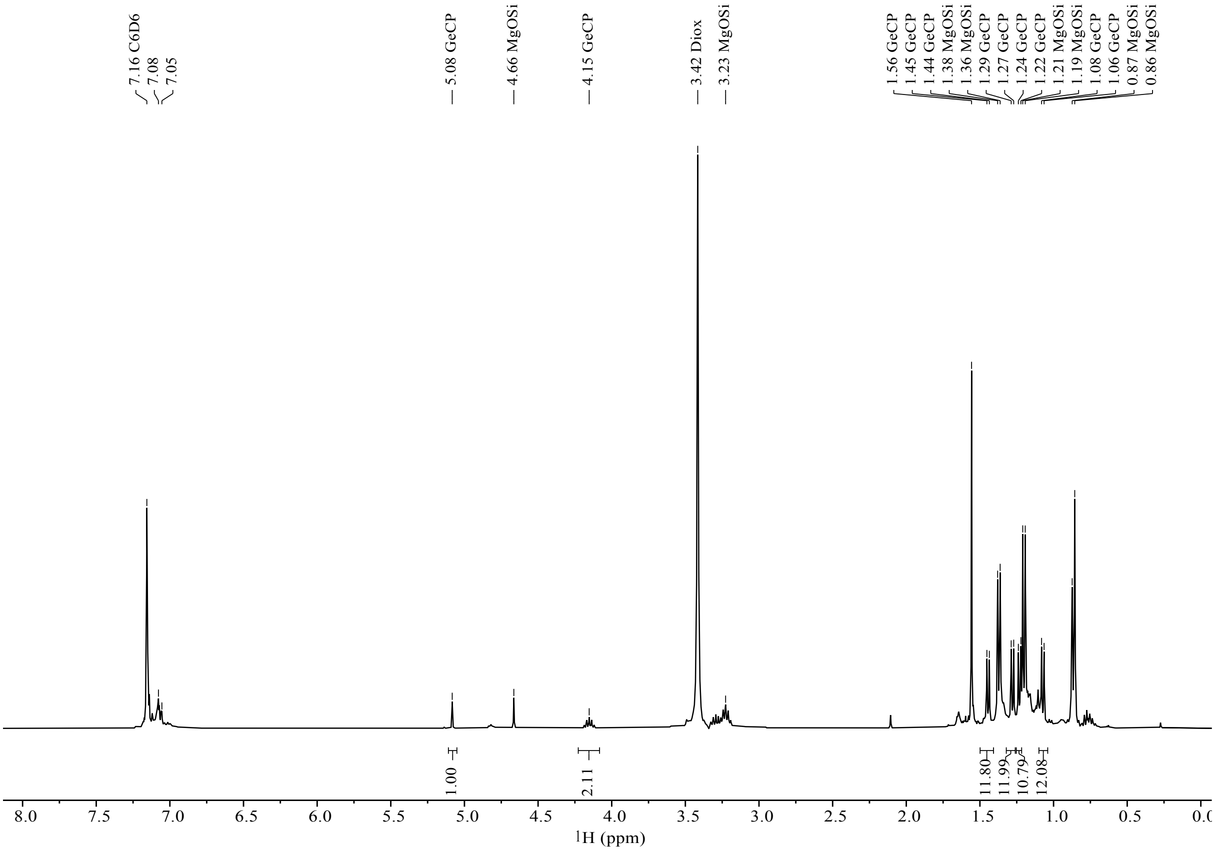

Figure S14. Representative ^{1}H NMR spectrum (400 MHz, 295 K) for the *in situ* generation of **7** in the presence of **2**.

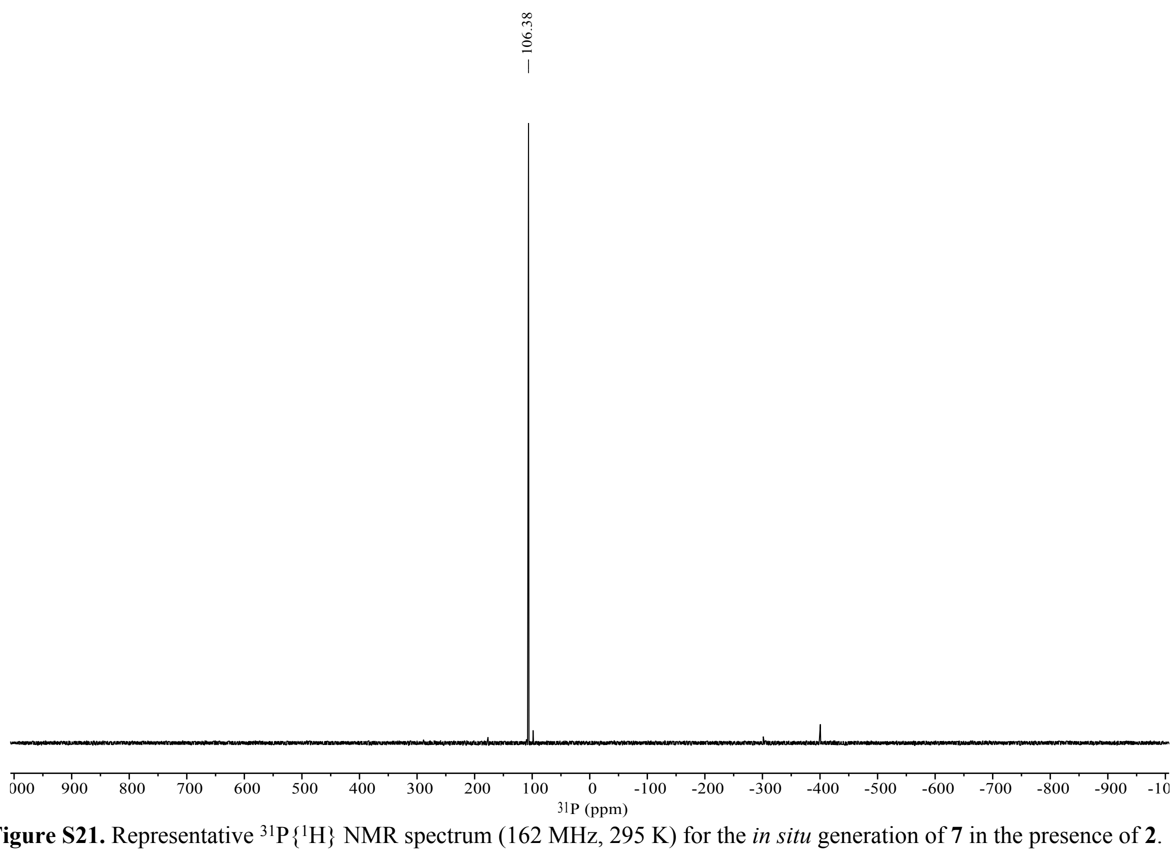

Figure S21. Representative ^{31}P{^{1}H} NMR spectrum (162 MHz, 295 K) for the *in situ* generation of **7** in the presence of **2**.

1.9. NMR spectra for [Sn(^{Dipp}NacNac)(CP)] (**8**)

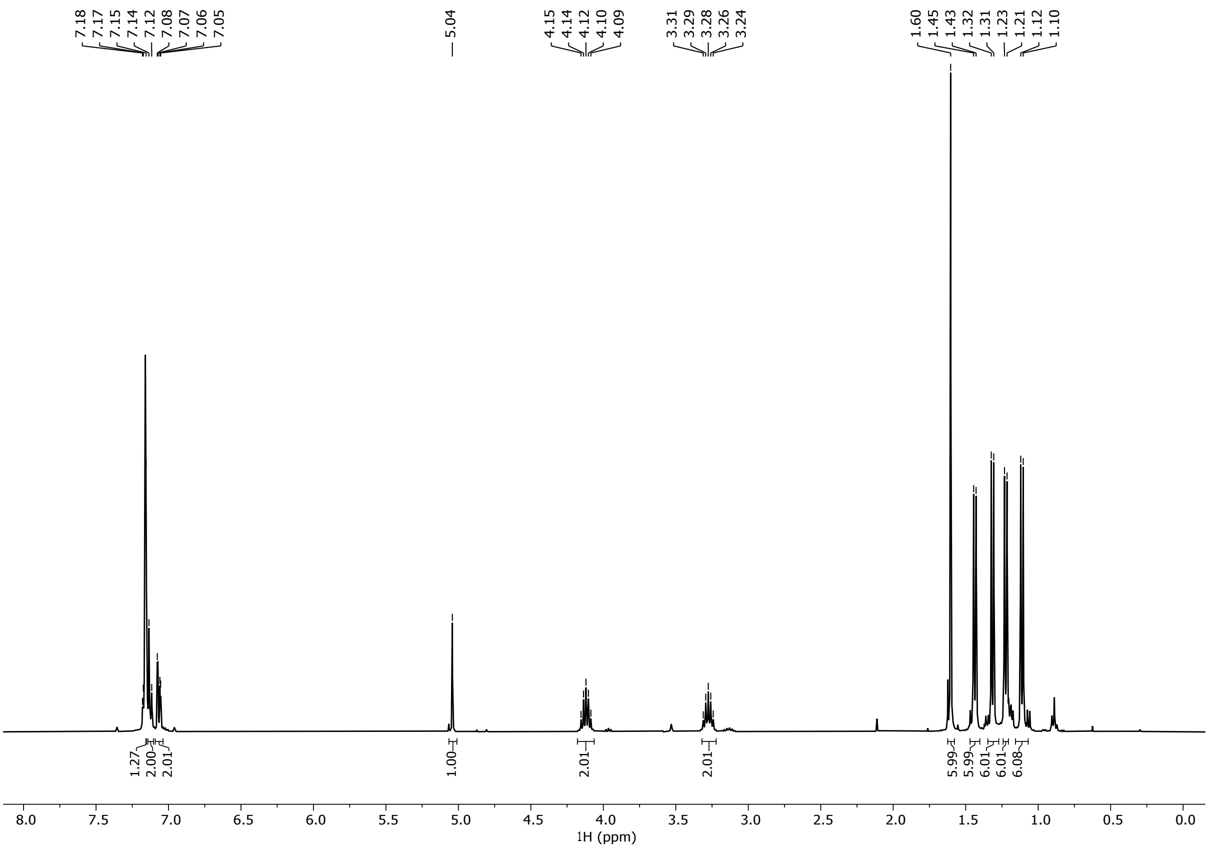

Figure S15. ^{1}H NMR spectrum (400 MHz, 295 K) of **8** in C_6D_6.

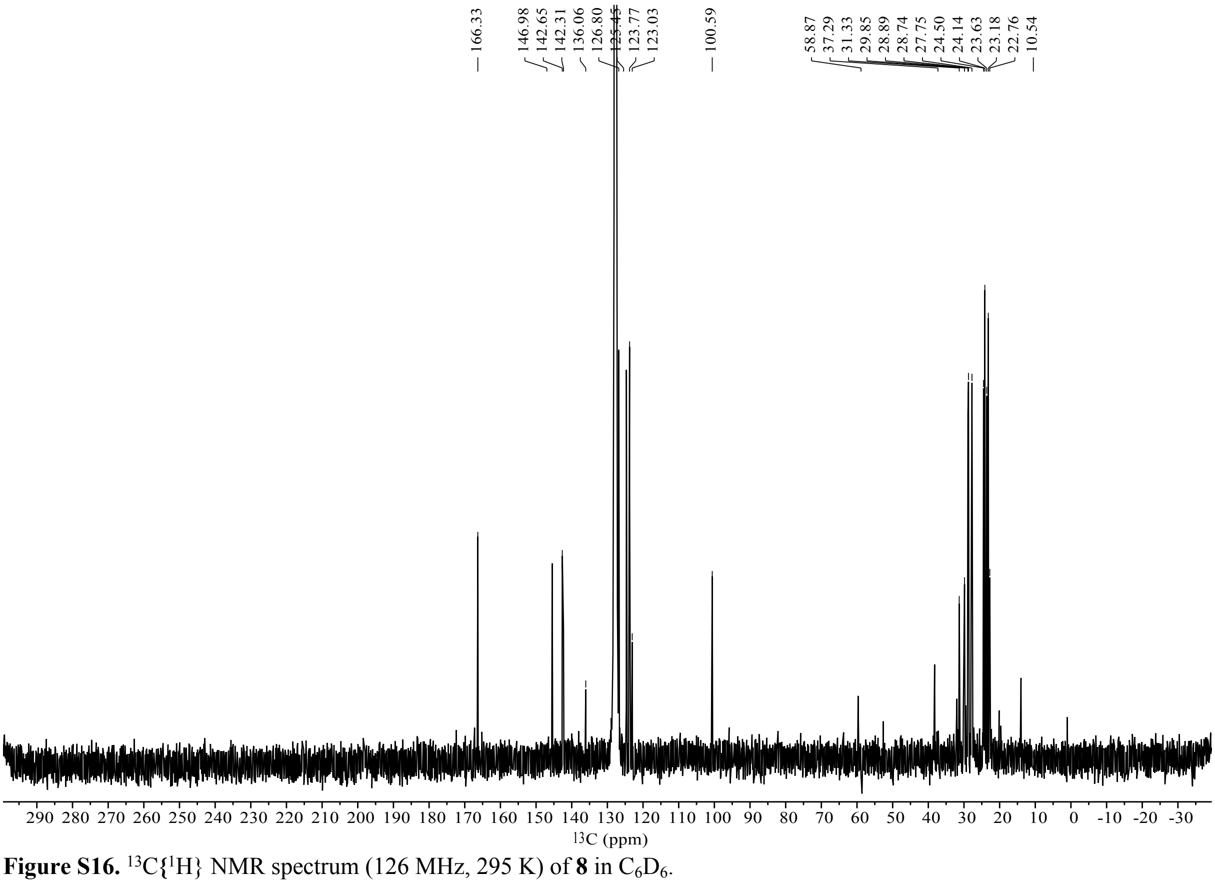

Figure S16. $^{13}C\{^{1}H\}$ NMR spectrum (126 MHz, 295 K) of **8** in C_6D_6.

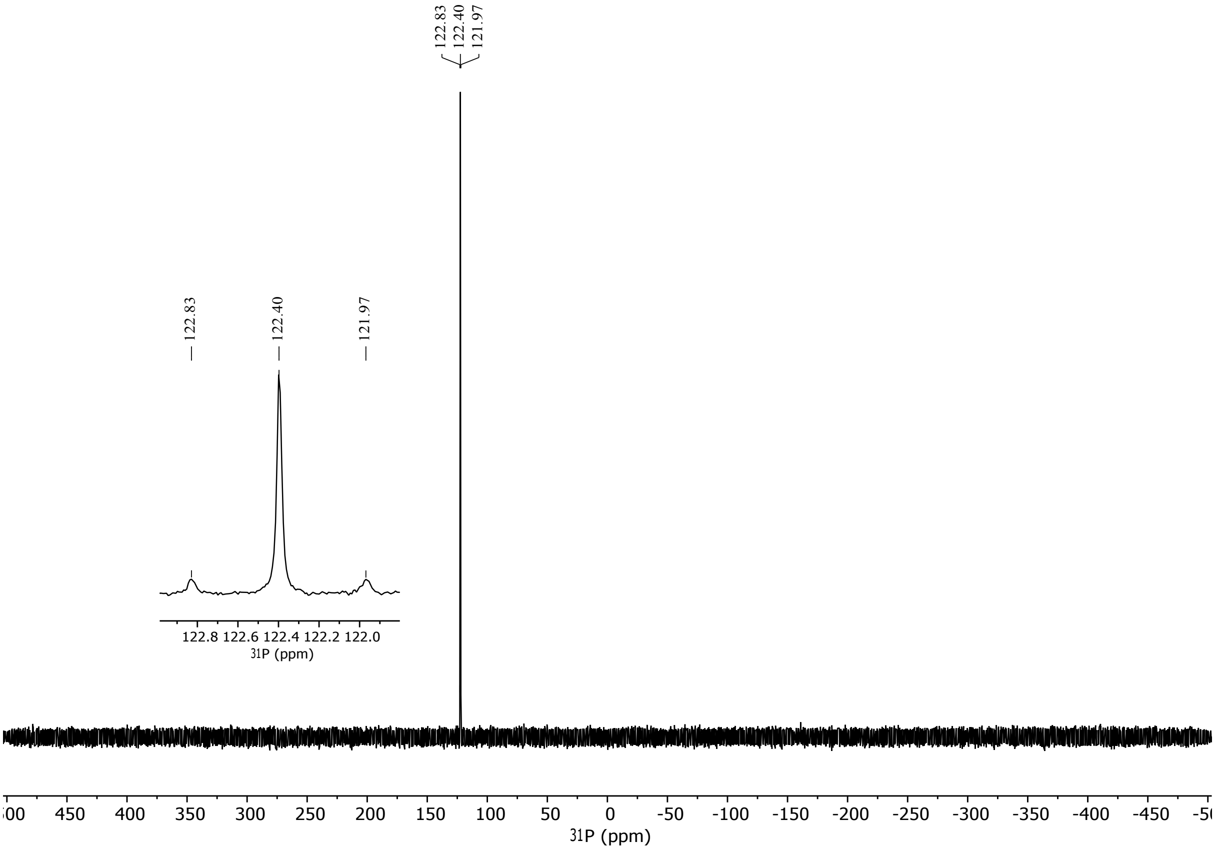

Figure S24. $^{31}P\{^{1}H\}$ NMR spectrum (162 MHz, 295 K) of **8** in C_6D_6.

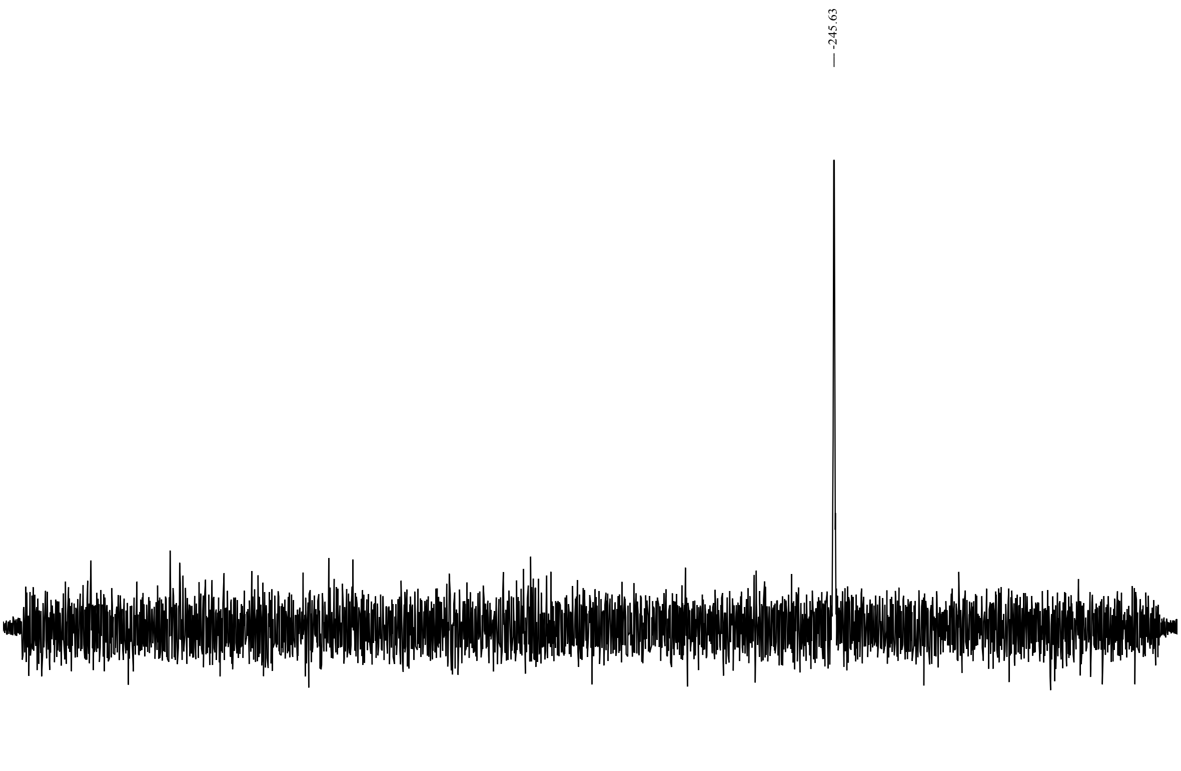

Figure S25. $^{119}Sn\{^{1}H\}$ NMR spectrum (186 MHz, 295 K) of **8** in C_6D_6.

1.10. NMR spectra for [Au(IDipp)(CP)] (**9**)

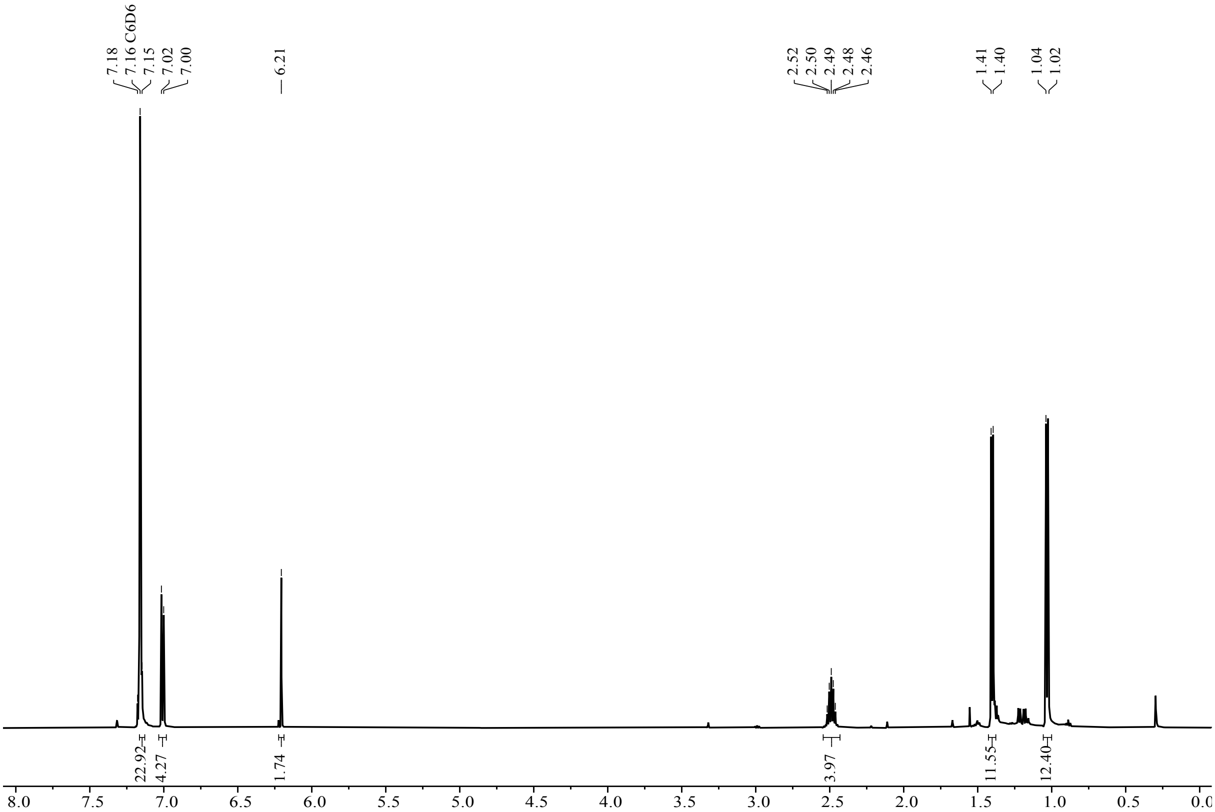

Figure S17. ^{1}H NMR spectrum (500 MHz, 295 K) of **9** in C_6D_6.

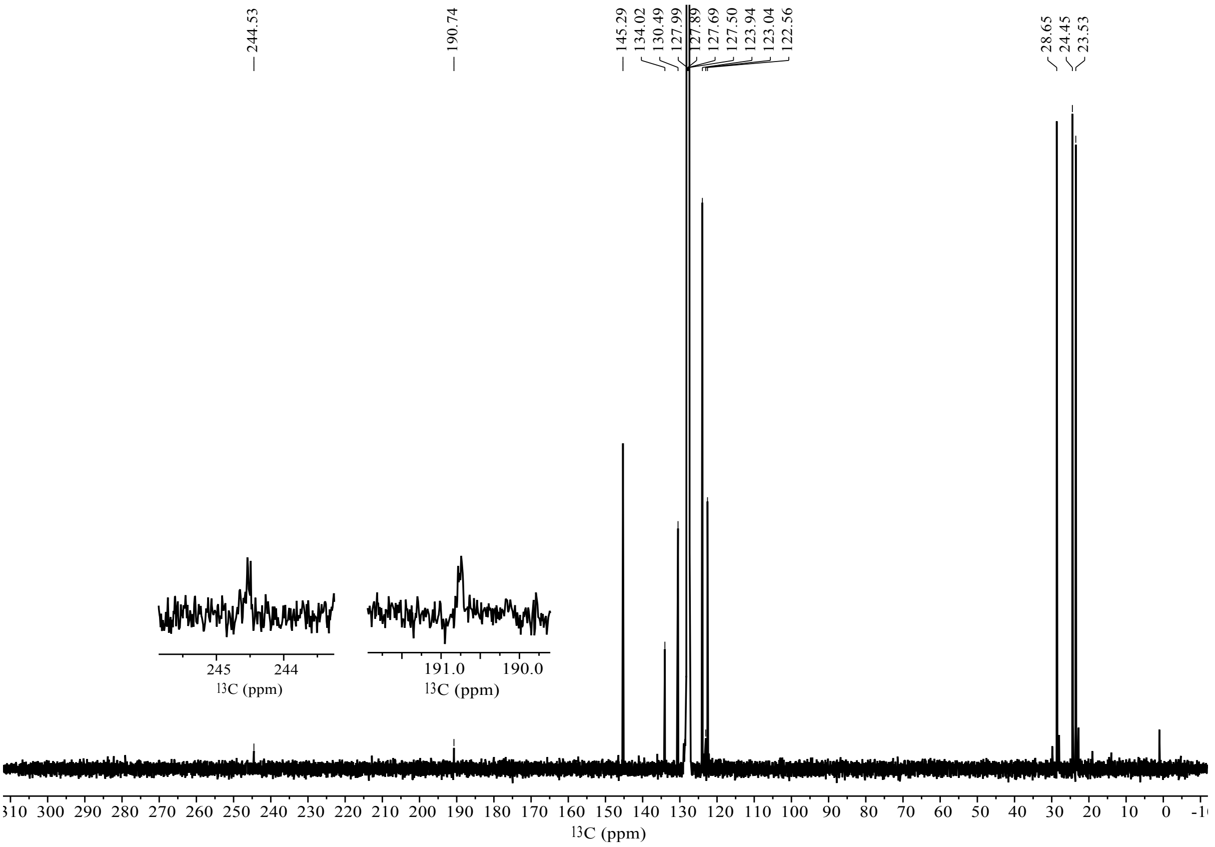

Figure S18. $^{13}C\{^{1}H\}$ NMR spectrum (126 MHz, 295 K) of **9** in C_6D_6.

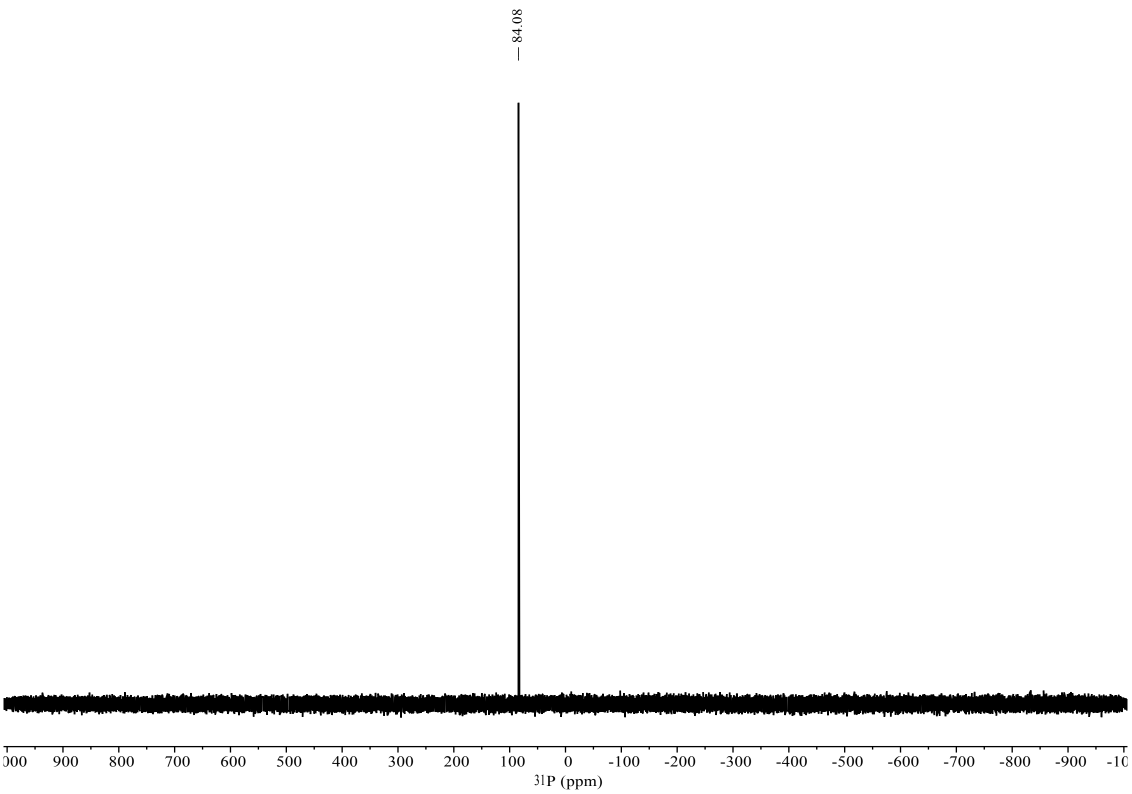

Figure S28. $^{31}P\{^{1}H\}$ NMR spectrum (162 MHz, 295 K) of **9** in C_6D_6.

1.11. NMR spectra for [(^{Dipp}PDI)Co(CP)] (**10**)

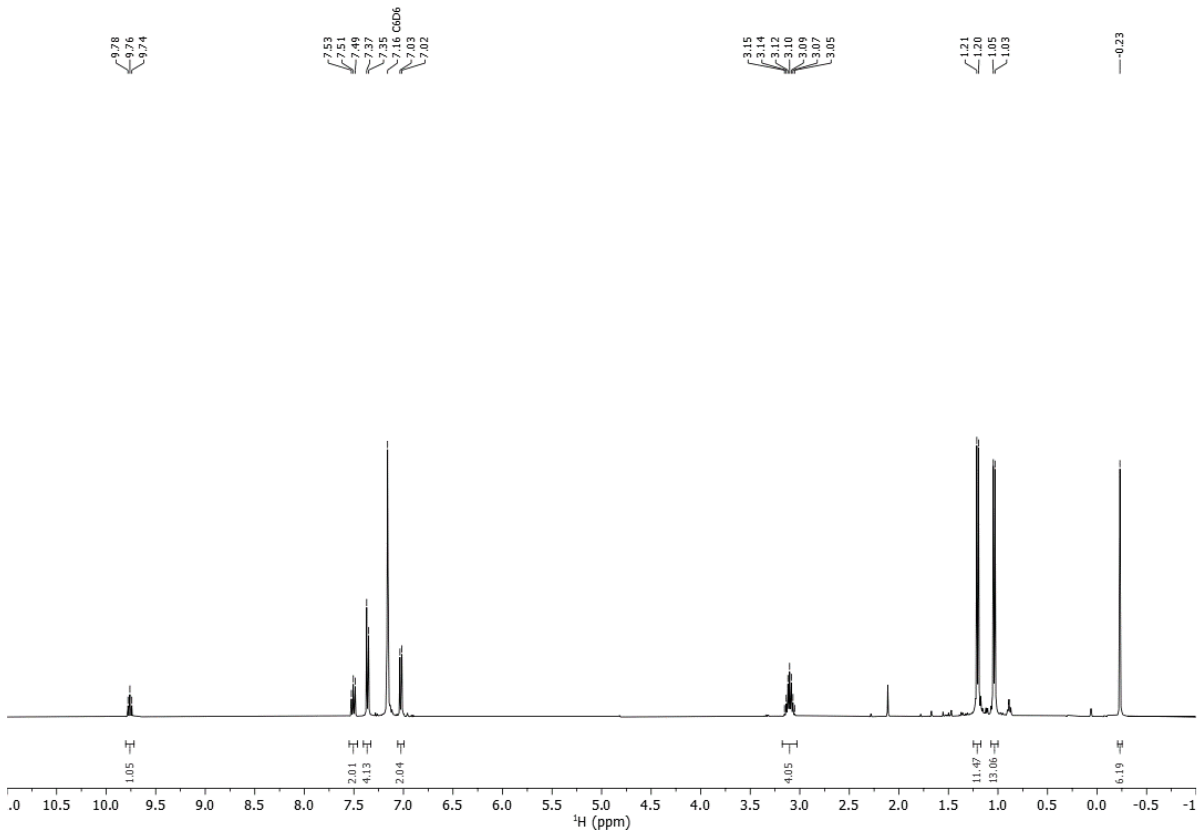

Figure S29. ^{1}H NMR spectrum (400 MHz, 295 K) of **10** in C_6D_6.

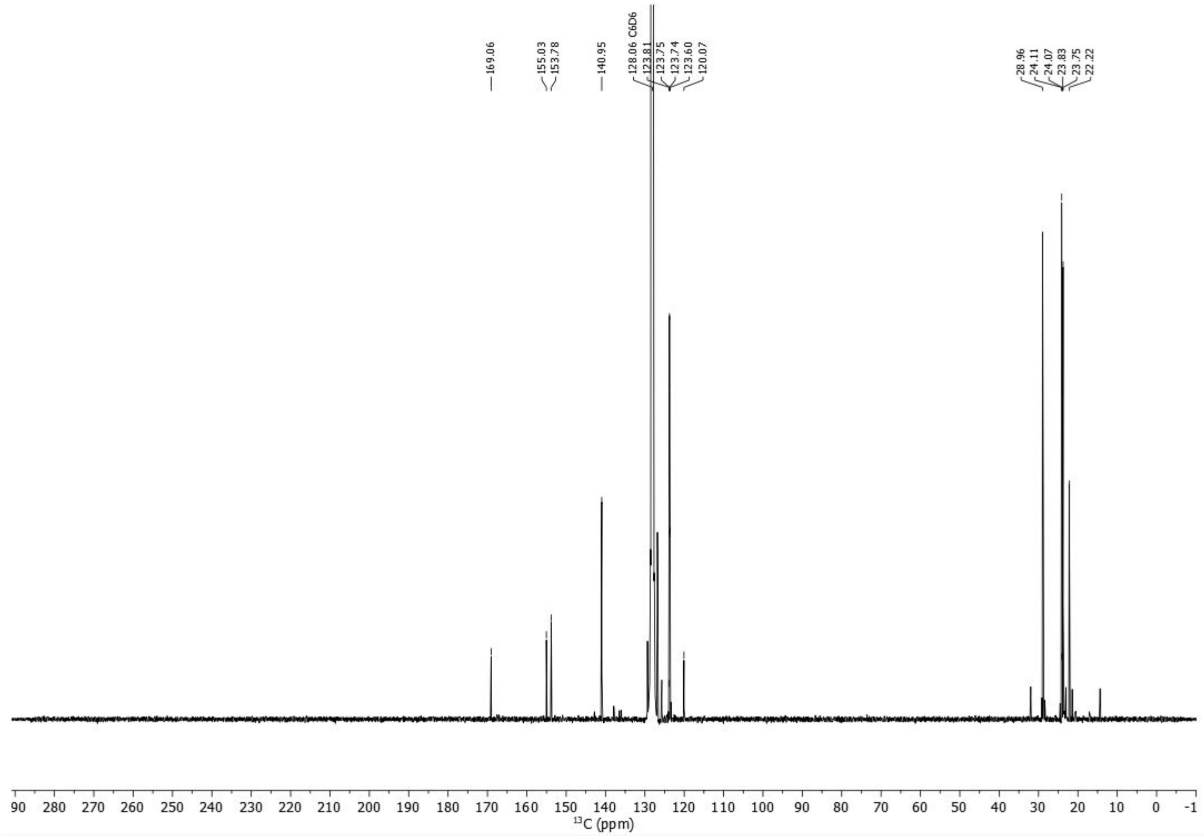

Figure S30. $^{13}C\{^{1}H\}$ NMR spectrum (151 MHz, 295 K) of **10** in C_6D_6.

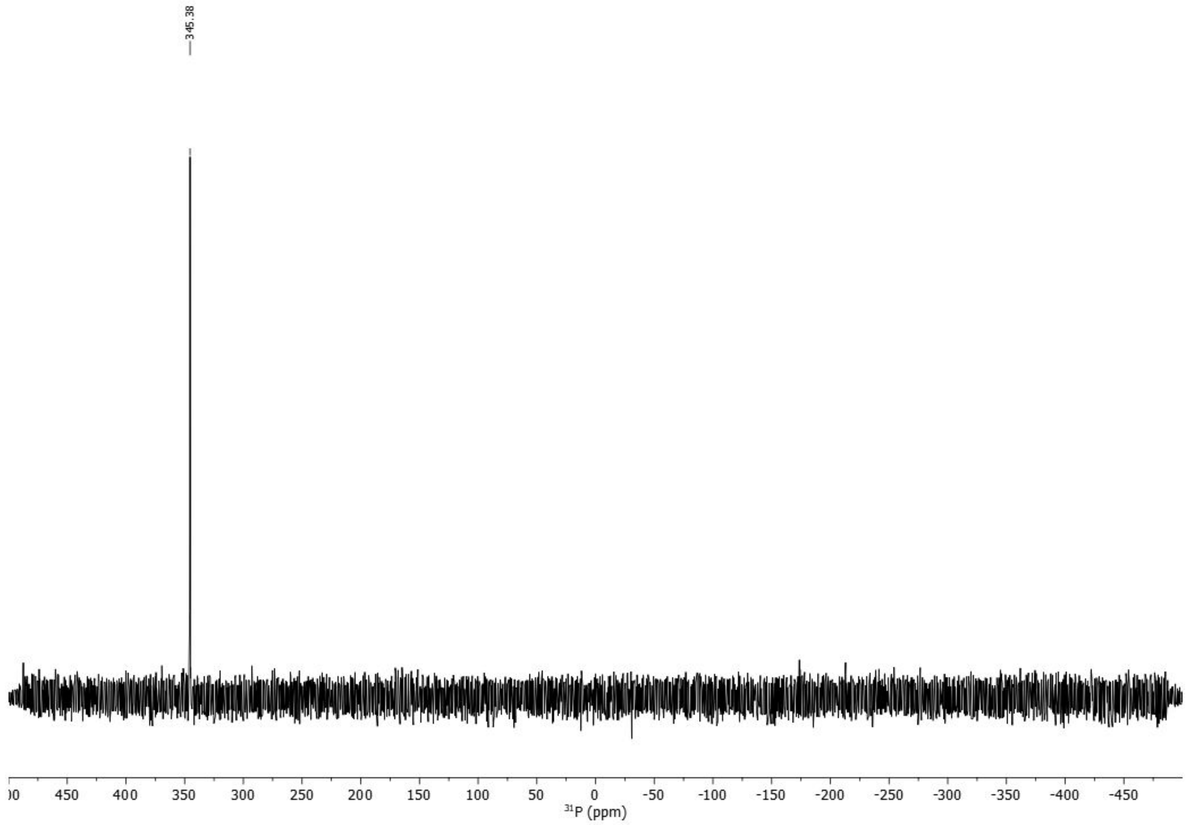

Figure S31. $^{31}P\{^{1}H\}$ NMR spectrum (162 MHz, 295 K) of **10** in C_6D_6.

1.12. NMR spectra for [Mg(^{Dipp}NacNac)Cl]

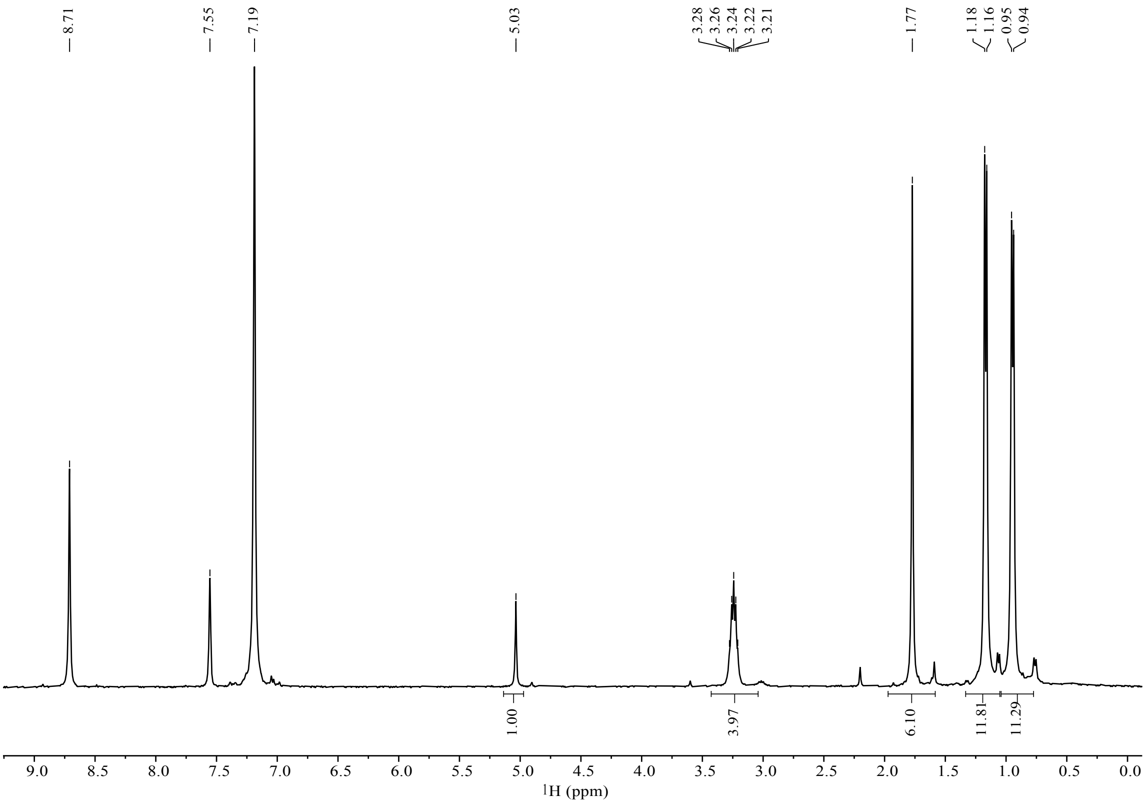

Figure S32. ^{1}H NMR spectrum (500 MHz, 295 K) of [Mg(^{Dipp}NacNac)Cl] in pyridine-d_5.

2. ATR-IR spectra of cyaphide complexes

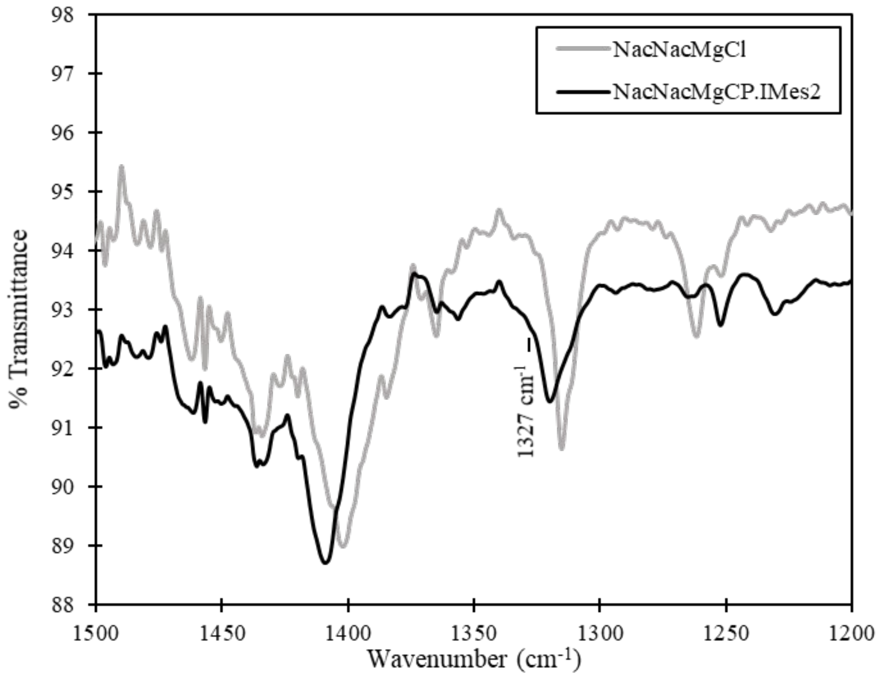

Figure S33. ATR-IR of **5** and [Mg(DippNacNac)Cl].

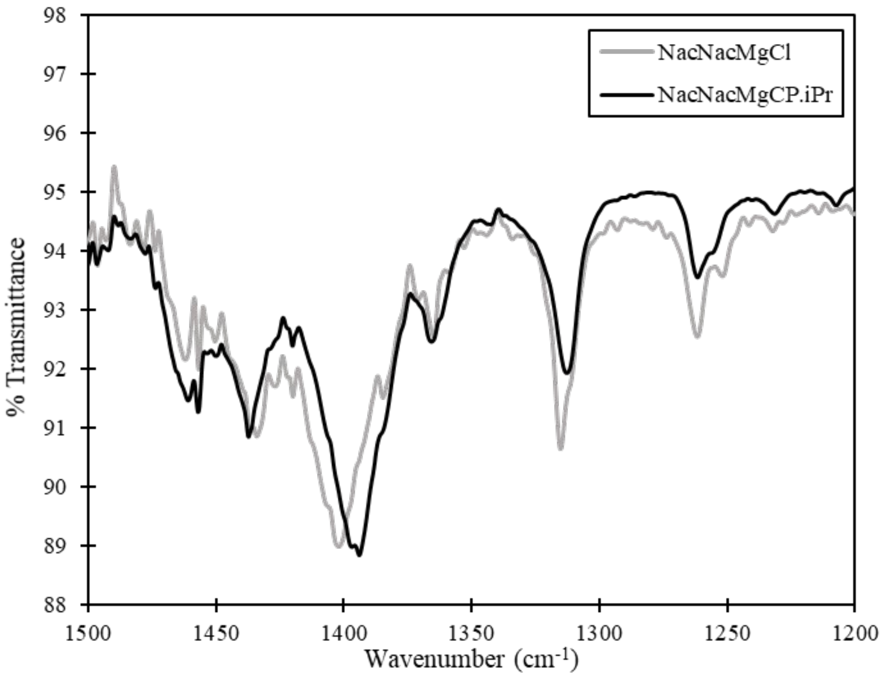

Figure S34. ATR-IR of **6** and [Mg(^{Dipp}NacNac)Cl].

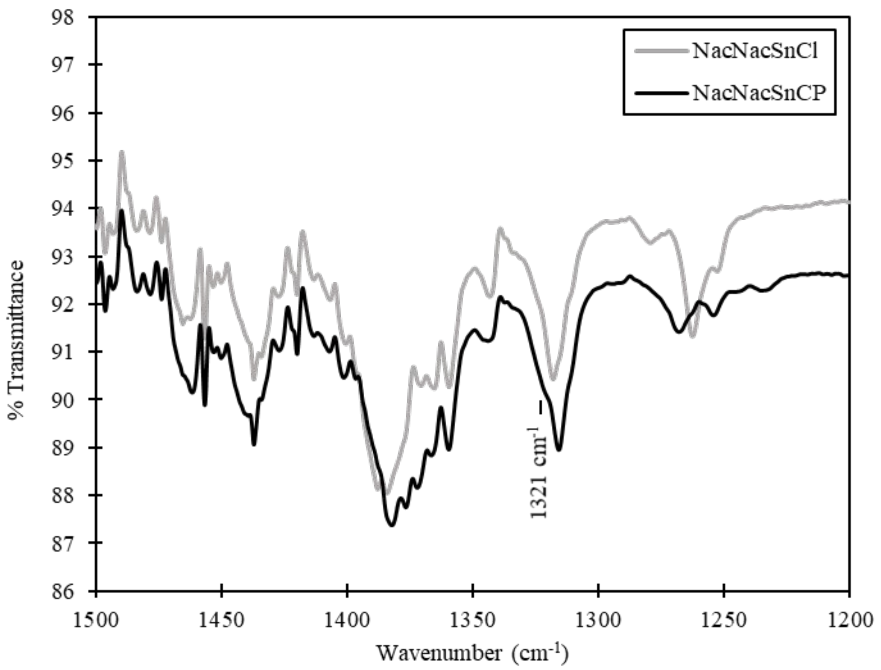

Figure S35. ATR-IR of **8** and [Sn(^{Dipp}NacNac)Cl]

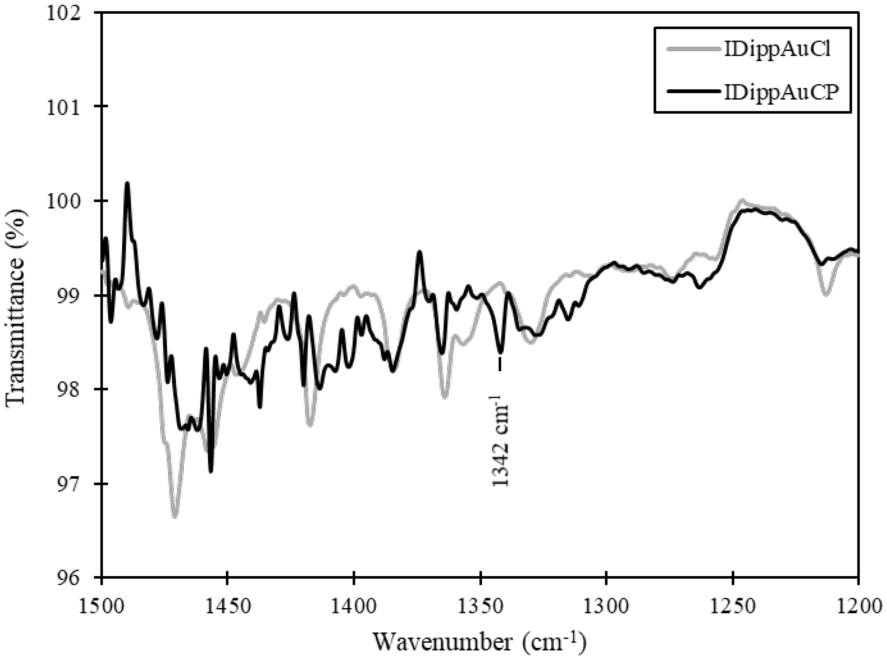

Figure S36. ATR-IR of **9** and [Au(IDipp)Cl].

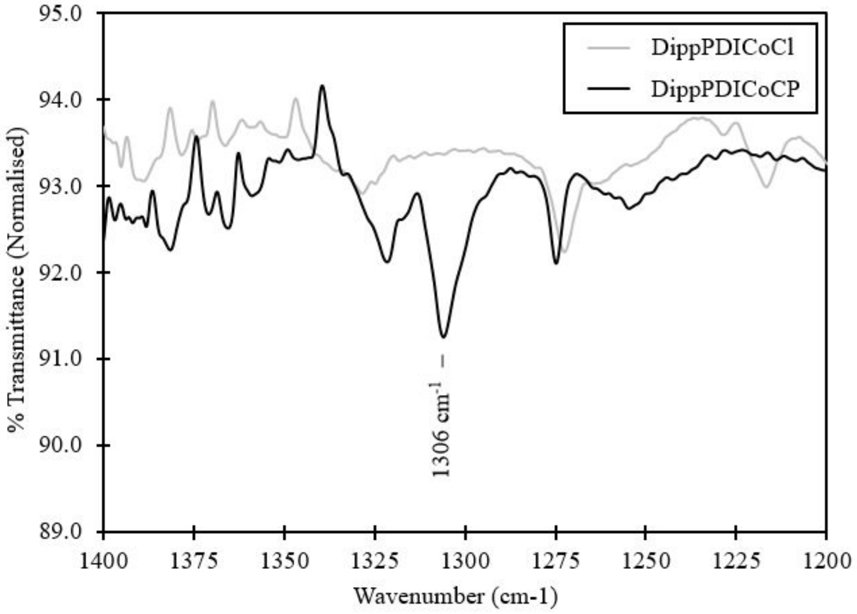

Figure S37. ATR-IR of **10** and [Co(^{Dipp}PDI)Cl].

3. Single crystal X-ray diffraction data

Single-crystal X-ray diffraction data were collected using an Oxford Diffraction Supernova dual-source diffractometer equipped with a 135 mm Atlas CCD area detector. Crystals were selected under Paratone-N oil, mounted on micromount loops and quench-cooled using an Oxford Cryosystems open flow N_2 cooling device. Data were collected at 150 K using mirror monochromated Cu K_α (λ = 1.54184 Å) or Mo K_α (λ = 0.71073 Å) radiation and processed using the CrysAlisPro package, including unit cell parameter refinement and inter-frame scaling (which was carried out using SCALE3 ABSPACK within CrysAlisPro).[2] Equivalent reflections were merged and diffraction patterns processed with the CrysAlisPro suite. Structures were subsequently solved using direct methods.[3]

Table S1. Selected X-ray data collection and refinement parameters for **1**, **2**, **3** and **5**·IMes.

	**1**	**2**	**3**	**5**·IMes
Formula	$C_{34}H_{49}MgN_2O_2P$	$C_{42}H_{70}MgN_2O_3Si$	$C_{34}H_{49}MgN_2OP$	$C_{72}H_{89}MgN_6P$
CCDC	2078056	2078057	2078058	2078059
Fw [g mol^{-1}]	573.03	703.40	557.03	1093.77
Crystal system	monoclinic	monoclinic	orthorhombic	monoclinic
Space group	$P2_1/c$	$P2_1/n$	$P2_12_12_1$	$P2_1/n$
a (Å)	17.3179(5)	11.5488(1)	9.7611(2)	14.4725(3)
b (Å)	11.7439(5)	20.5766(2)	16.1991(4)	21.5957(3)
c (Å)	16.7377(6)	17.9650(1)	21.4038(3)	21.1743(4)
α (°)	90	90	90	90
β (°)	102.437(4)	92.549(1)	90	98.667(2)
γ (°)	90	90	90	90
V (Å^3)	3324.2(2)	4264.89(6)	3384.39(12)	6542.3(2)
Z	4	4	4	4
Radiation, λ (Å)	Cu Kα, 1.54184	Cu Kα, 1.54184	Cu Kα, 1.54184	Cu Kα, 1.54184
Temp (K)	150(2)	150(2)	150(2)	150(2)
ρ_{calc} (g cm^{-3})	1.145	1.095	1.093	1.110
μ (mm^{-1})	1.147	0.906	1.091	0.798
Reflections collected	18539	48288	12403	31707
Independent reflections	6935	8888	6382	13557
Parameters	371	458	363	742
R(int)	0.0628	0.0305	0.0280	0.0308
R1/wR2,[a] I ≥ 2σI (%)	4.62/9.98	4.38/11.60	5.01/13.25	4.88/11.80
R1/wR2,[a] all data (%)	8.26/11.49	5.05/12.30	5.86/14.03	8.13/13.44
GOF	1.011	1.037	1.031	1.032

$R1 = [\Sigma||F_o| - |F_c||]/\Sigma|F_o|$; $wR2 = \{[\Sigma w[(F_o)^2 - (F_c)^2]^2]/[\Sigma w(F_o^2)^2\}^{1/2}$; $w = [\sigma^2(F_o)^2 + (AP)^2 + BP]^{-1}$, where $P = [(F_o)^2 + 2(F_c)^2]/3$ and the A and B values are 0.0439 and 0.27 for **1**, 0.0617 and 1.60 for **2**, 0.0758 and 0.69 for **3**, and 0.0576 and 0.80 for **5**·IMes.

Table S2. Selected X-ray data collection and refinement parameters for **6**, **8**, **9** and **10**.

	**6**	**8**	**9**	**10**
Formula	$C_{39}H_{57}MgN_4P$	$C_{30}H_{41}N_2PSn$	$C_{28}H_{36}AuN_2P$	$C_{34}H_{43}CoN_3P$
CCDC	2078060	2078061	2078062	2086971
Fw [g mol^{-1}]	637.16	579.31	628.52	583.61
Crystal system	monoclinic	triclinic	orthorhombic	orthorhombic
Space group	*C*2/*c*	*P*−1	$P2_12_12_1$	$P2_12_12_1$
a (Å)	39.9913(11)	10.6402(6)	12.6983(1)	8.4877(3)
b (Å)	10.7547(3)	12.3336(7)	14.2088(1)	17.8622(6)
c (Å)	19.9756(4)	12.4077(7)	15.6353(1)	20.1786(5)
α (°)	90	88.304(5)	90	90
β (°)	97.248(2)	69.334(5)	90	90
γ (°)	90	70.944(5)	90	90
V (Å^3)	8522.7(4)	1433.26(15)	2821.04(3)	3059.26(17)
Z	8	2	4	4
Radiation, λ (Å)	Cu Kα, 1.54184	Mo Kα, 0.71073	Cu Kα, 1.54184	Cu Kα, 1.54184
Temp (K)	150(2)	150(2)	150(2)	150(2)
ρ_{calc} (g cm^{-3})	0.993	1.342	1.480	1.267
μ (mm^{-1})	0.913	0.967	10.445	5.084
Reflections collected	32215	21091	95325	23760
Independent reflections	7505	7335	5918	6325
Parameters	420	317	297	362
R(int)	0.0459	0.0529	0.0585	0.0544
R1/wR2,[a] I ≥ 2σI (%)	5.92/16.22	4.10/7.35	2.97/7.47	4.21/1047
R1/wR2,[a] all data (%)	7.81/17.36	5.73/8.10	3.16/7.69	5.03/11.05
GOF	1.047	1.043	1.121	1.018

$R1 = [\Sigma||F_o| - |F_c||]/\Sigma|F_o|$; $wR2 = \{[\Sigma w[(F_o)^2 - (F_c)^2]^2]/[\Sigma w(F_o^2)^2\}^{1/2}$; $w = [\sigma^2(F_o)^2 + (AP)^2 + BP]^{-1}$, where $P = [(F_o)^2 + 2(F_c)^2]/3$ and the A and B values are 0.0877 and 5.71 for **6**, 0.0208 and 0.93 for **8**, 0.0562 and 0.88 for **9**, and 0.0616 and 0.65 for **10**.

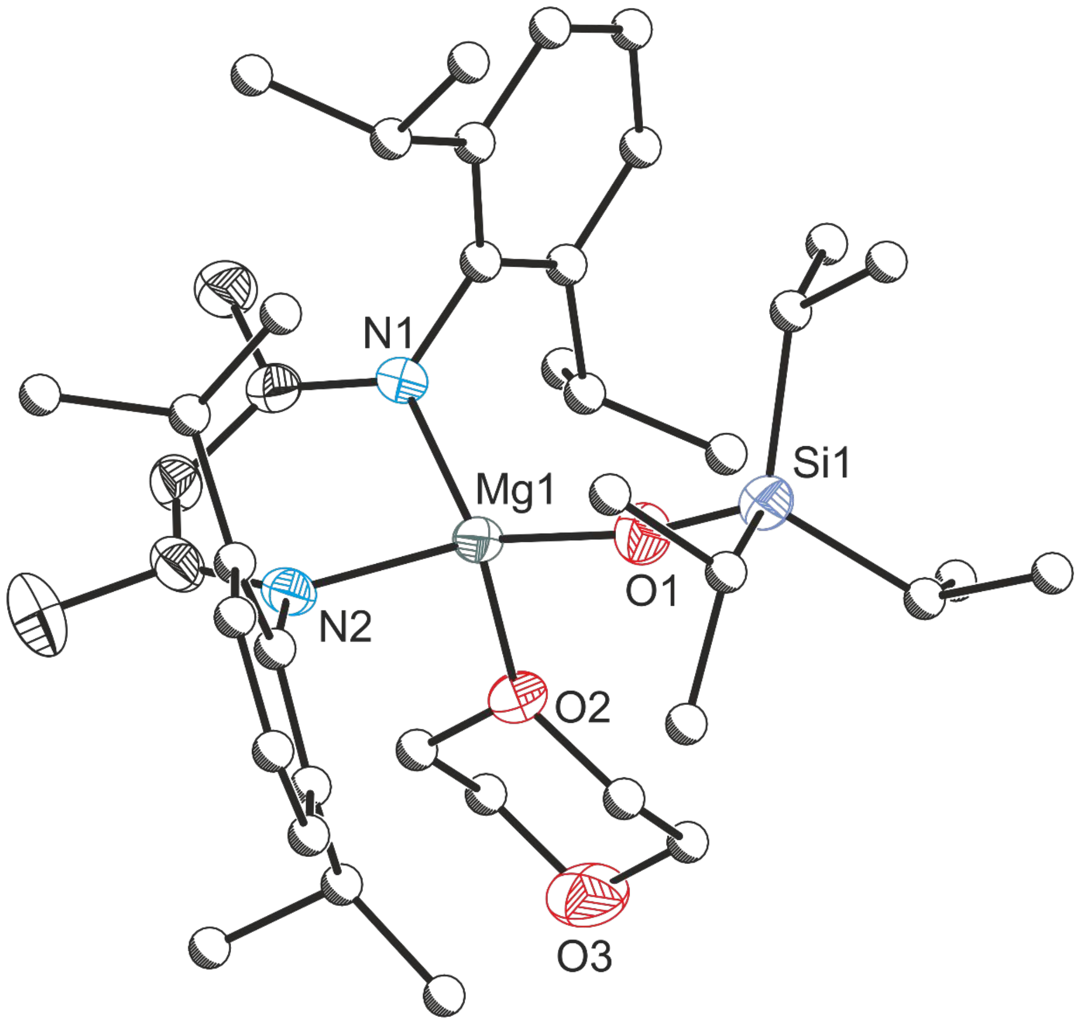

Figure S38. Single crystal X-ray structure of **2** (anisotropic displacement ellipsoids set at 50% probability; hydrogen atoms omitted for clarity; carbon atoms of Dipp and dioxane are pictured as spheres of arbitrary radius).

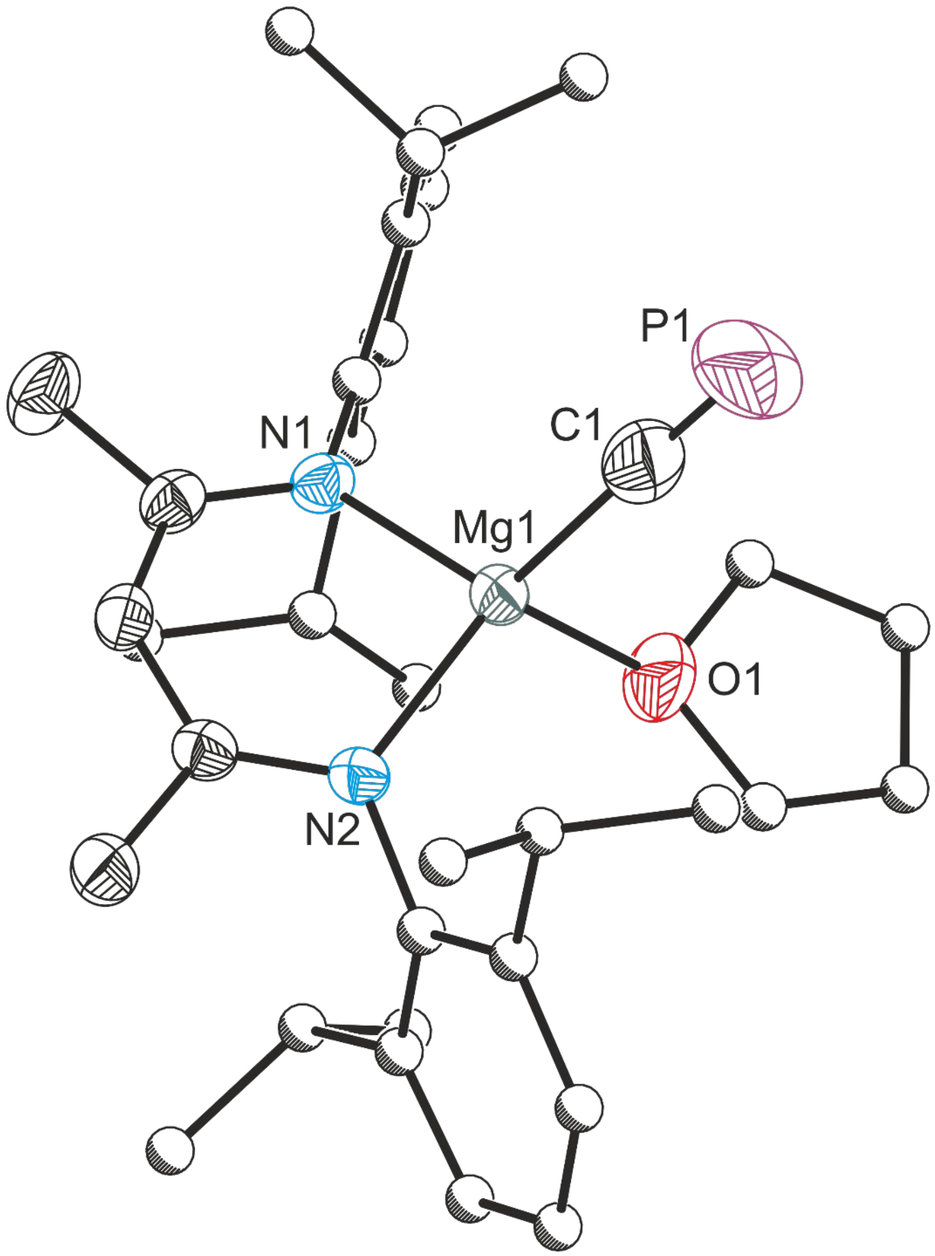

Figure S39. Single crystal X-ray structure of **3** (anisotropic displacement ellipsoids set at 50% probability; hydrogen atoms omitted for clarity; carbon atoms of Dipp and THF are pictured as spheres of arbitrary radius).

4. Computational details

3.1 General computational methods

All calculations were carried out using Orca 4.2.0[4,5] All methods were used as implemented. Geometry optimizations and single point calculations were performed for all structures using Becke's '88 gradient exchange functional,[6] and Perdew's '86 correlation functional (BP86),[7] with the resolution of identity (RI-J) approximation.[8] Geometry optimizations were performed using the Karlsruhe split valence basis set (def2-SVP) with the general Weigend J auxiliary basis set (def2/J), and single point energies were obtained using the Karlsruhe triple zeta valence basis set (def2-TZVP).[9,10] Single point energies were corrected using Grimme's D3 dispersion correction with Becke-Johnson damping (D3BJ),[11] and gCP geometric counterpoise correction.[12] For structures containing Sn or Au, relativistic calculations were done using the ZORA method, using the SARC-ZORA-SVP basis set for Au, and the ZORA contracted def2-SVP basis set for all other atoms, as well as the SARC/J auxiliary basis set by Pantazis et al.[13,14]

^{31}P NMR predictions were performed using optimized geometries obtained by the PBEh-3c composite method developed by Grimme and coworkers,[15] which uses a composite valence-double-zeta Gaussian AO basis set (def2-mSVP) for all atoms. The NMR shielding tensors were calculated using the Gauge Including Atomic Orbitals (GIAO) method,[16–18] and were carried out with the one-parameter hybrid Perdew-Burke-Ernzerhof (PBE0) functional,[19] using Jensen's segmented contracted pcsSeg-3 basis set for phosphorus,[20] and the composite def2-mSVP basis set for all other atoms. Relative chemical shifts were obtained using H_3PO_4 in water as a reference (δ = 0 ppm), and scaled using an empirical linear scaling method that has been previously shown to improve the accuracy of chemical shift predictions.[21–35]

Solvent corrections for all structures were performed using the conductor-like continuum polarization model (C-PCM), using spherical gaussian charges and a scaled van der Waals solute cavity surface.[26,27] All stationary points were identified as being minima (NIMAG = 0) or saddle points (NIMAG = 1) by frequency calculations.

3.2 Calculated energies and mechanisms

Table S3. Relative enthalpies, entropies, and Gibbs free energies (at 298.15 K) for the mechanism of the formation of **C** in benzene (BP86/def2-TZVP); A: [Mg(^{Dipp}NacNac)]$_2$ + ^{i}Pr$_3$SiOCP, B: Intermediate, C: [Mg(^{Dipp}NacNac)(CP)] + [Mg(^{Dipp}NacNac)(OSi^{i}Pr$_3$)].

	ΔH<br>kcal / mol	ΔS<br>cal / mol K	ΔG<br>kcal / mol
A	0.00	0.00	**0.00**
TS1	−3.96	−55.11	**12.47**
B	−41.34	−55.84	**−24.69**
TS2	−38.02	−59.40	**−20.31**
C	−49.42	9.29	**−52.19**

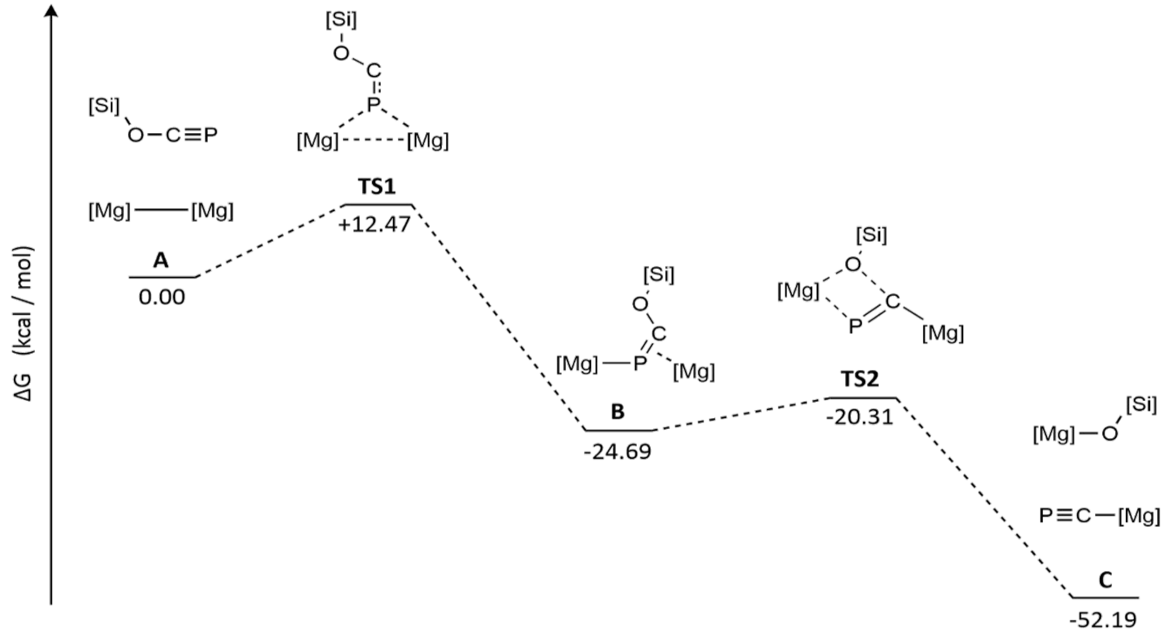

Figure S40. Energy diagram for the mechanism of the formation of **C** in benzene (BP86/def2-TZVP).

Table S4. Relative enthalpies, entropies, and Gibbs free energies (at 298.15 K) for the isomerization mechanism of $^{i}Pr_3SiOCP$ to $^{i}Pr_3SiPCO$ in benzene (BP86/def2-TZVP).

	ΔH<br>kcal / mol	ΔS<br>cal / mol K	ΔG<br>kcal / mol
$^{i}Pr_3SiOCP$	0.00	0.00	**0.00**
TS	25.83	−1.55	**26.68**
$^{i}Pr_3SiPCO$	−10.41	0.17	**−10.77**

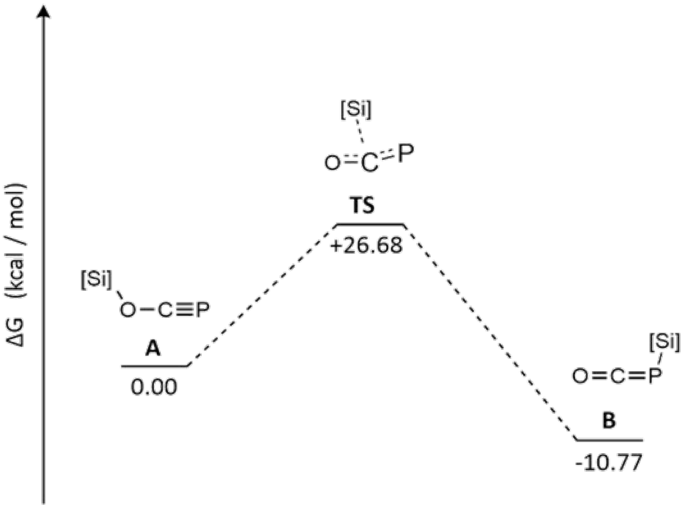

Figure S41. Energy diagram for the isomerization mechanism of $^{i}Pr_3SiOCP$ to $^{i}Pr_3SiPCO$ in benzene (BP86/def2-TZVP).

3.3. Calculated C≡P stretching frequencies

Table S5. Calculated vibrational modes corresponding to C≡P stretches for cyaphide compounds (BP86/def2-TZVP). *Not measured due to compound instability. [a] On the shoulder of an adjacent stretch. Experimentally determined C−P bond lengths also provided for comparison

	Calc. v (cm^{-1})	*IR Intensity (km/mol)*	*Exp. v (cm^{-1})*	*C−P bond length (Å)*
$(CF_3)_3$B(C≡P)	**1461.31**	21.944	**1468**	1.563(10)
[Ru(dppe)$_2$(H)(C≡P)]	**1230.59**	92.115	**1229**	1.573(2)
[Mg(^{Dipp}NacNac)(dioxane)(C≡P)]	**1327.14**	9.627	*	1.553(2)
[Mg(^{Dipp}NacNac)(IMes)(C≡P)]	**1310.73**	4.424	**1327**[a]	1.550(2)
[Mg(^{Dipp}NacNac)(I^{i}Pr)(C≡P)]	**1311.85**	4.891	**Not observed**	1.531(3)
[Sn(^{Dipp}NacNac)(C≡P)]	**1327.30**	12.250	**1321**[a]	1.542(4)
[(IDipp)Au(C≡P)]	**1331.61**	62.954	**1342**	1.552(6)
[(^{Dipp}PDI)Co(C≡P)]	**1282.60**	21.886	**1306**	1.506(4)

3.4. Calculated ^{31}P NMR shifts

Table S6. Calculated ^{31}P NMR shifts for of CP compounds relative to H_3PO_4 in water (PBE0/def2-mSVP, pcSseg-3(P)). *Scaled parameters were obtained using a linear regression method, where $\delta_{scaled} = 0.94(\delta_{unscaled}) - 4.13$.

	abs shielding (ppm)	rel shift (ppm)	scaled shift (ppm)*	experimental (ppm)
H_3**P**O_4	294.4	0.0	**−4.1**	**0**
[Mg(^{Dipp}NacNac)(dioxane)(C≡**P**)]	94.4	200.0	**184.5**	**177**
[Mg(^{Dipp}NacNac)(IMes)(C≡**P**)]	116.2	178.2	**164.0**	**163**
[Mg(^{Dipp}NacNac)(I^{i}Pr^{normal})(C≡**P**)]	104.1	190.4	**175.4**	**175**
[Mg(^{Dipp}NacNac)(I^{i}Pr^{abnormal})(C≡**P**)]	107.4	174.0	**172.3**	**169**
[Ge(^{Dipp}NacNac)(C≡**P**)]	176.6	117.9	**107.0**	**106**
[Sn(^{Dipp}NacNac)(C≡**P**)]	150.3	144.1	**131. 8**	**122**
[(IDipp)Au(C≡**P**)]	207.0	87.4	**78.3**	**84**
[(^{Dipp}PDI)Co(C≡**P**)]	−71.1	365.5	**340.6**	**345**
$(CF_3)_3$B(C≡**P**)	245.1	49.4	**42.4**	**40**
[Ru(dppe)$_2$(H)(C≡**P**)]	127.4	167.1	**153.4**	**165**

3.5. XYZ coordinates

[Mg(^{Dipp}NacNac)(dioxane)(CP)]

BP86/def2-SVP

Mg	-0.03018479046469	0.04313664446977	-0.00876107174975
C	-0.15238290997797	0.28367610011576	2.10885502182748
P	-0.27306954324589	0.48259741459234	3.67705664484083
O	-0.07326912265800	2.08912034743860	-0.60525947566935
H	-2.12474394260470	2.19954034375155	-0.36217515568401
H	1.98642367081216	2.22305577708390	-0.70465341716763
C	-1.26821218511295	2.90062177396258	-0.42047786730173
C	1.12277968248021	2.91291334372909	-0.61977988337770
H	1.31051118346239	3.11501526381384	1.53312722267056
H	-1.09983974176461	3.09869000448259	1.73077269243937
H	-1.38789088068190	3.53889842840191	-1.32429781592707
H	1.08220203910283	3.55620975876456	-1.52756209701209
C	-1.13618881005095	3.75945520244251	0.83174224830620
C	1.19077801954894	3.77231064293995	0.63852189534057
H	-2.01126842970015	4.43554307638115	0.91752037682241
H	2.06278702430004	4.45536515637763	0.58084074378896
O	0.02650422882390	4.58034200950521	0.76178722357860
N	-1.53899390440944	-0.94289984978285	-1.07847032126931
N	1.53982067217906	-0.99432461981058	-0.92328038557115
C	2.47198993870919	1.65633951507853	-4.15026237010670
C	-1.58317656763272	1.58538474788243	-4.04229136892872
C	-5.26028133497349	1.06469877221082	-1.58167203015210
C	4.57210599499541	0.76948864569154	-2.24753207365452
C	-4.36936129149279	0.95577902583905	-2.65621154616184
C	5.45270602871388	0.68891837389177	-1.15953949708173
C	2.34619746548698	0.31742857129531	-3.39855059583960

C	-4.93176876407459	0.47704952943537	-0.35223119871624
C	3.27880059687957	0.21249546352121	-2.18823813482874
C	-3.96847994074897	-0.11410711109015	2.38564953361130
C	-3.14418493758865	0.26809888563765	-2.53148165697089
C	-2.20587544799225	0.20561536808339	-3.73934847958976
C	5.03656901222651	0.05166561679988	0.01486125803495
C	3.45685064515726	-0.24144215016754	2.63800938023612
C	-3.72098375762112	-0.22169642416356	-0.16774475262983
C	-2.81489049533062	-0.32436936538624	-1.27243684003970
C	2.87040555812457	-0.46125823242378	-0.98977813729437
C	-2.90343913293460	-0.36219680171257	-4.99342763100793
C	3.75469304826439	-0.52770844975494	0.13058382160267
C	2.54704516944886	-0.85470343025579	-4.38438896332891
C	-3.41609994050485	-0.88674085158150	1.17589634606502
C	3.37152584281282	-1.21206263004980	1.44357829422364
C	-3.90570653353705	-2.35146476309760	1.20927696707324
C	-1.29122934735033	-2.17734871374365	-1.54685563533454
C	1.28765698872275	-2.25382465437881	-1.33378827719365
C	4.22991661674494	-2.46753028030318	1.70919105097215
C	-0.00923741542287	-2.79368149368030	-1.54092022166980
C	-2.40736855981577	-2.99478888776388	-2.17857216389912
C	2.44023160920870	-3.21734735353004	-1.58105047049276
H	2.38883885113399	2.52845968604382	-3.47042535382423
H	-2.36571726043418	2.34210756519416	-4.26071169695165
H	1.67218183739681	1.74532970972825	-4.91375804417090
H	-0.98142061656613	1.95097433984588	-3.18639110469948
H	3.43896588878956	1.74393563581060	-4.68883560445596
H	-0.91516101761291	1.52912456778158	-4.92732718825222
H	-6.21242971581216	1.60435338633831	-1.70191732247378
H	4.89904375084789	1.27876188317651	-3.16690731118703

H	-4.63085142734930	1.41793502342703	-3.62134529826501
H	6.46000891948050	1.12797405010672	-1.22708651396572
H	-3.67291480750459	0.95360089385646	2.36249755782206
H	1.30928967321518	0.24256833742471	-3.00627136587791
H	-5.63641401989511	0.55748882087682	0.48931147658387
H	2.82398234727641	0.65284176575434	2.47795113184793
H	-5.07632607870837	-0.16553271785238	2.44979880386420
H	4.49974873325584	0.09237038712477	2.82169188730576
H	-3.69543368630885	0.31878330460459	-5.37063940932822
H	5.72745078070180	-0.00344938691492	0.87130662815181
H	-3.56461864200323	-0.54577872138647	3.32402909374234
H	1.86108374745759	-0.75983334818418	-5.25172508805868
H	-1.36979776479376	-0.47682126811995	-3.48417107239663
H	-2.17196349111614	-0.50306657086039	-5.81618052574543
H	3.09466441077020	-0.73307265879672	3.56409940101592
H	3.58701462265429	-0.86766500018796	-4.77326349645158
H	-2.30988193174345	-0.90884148381558	1.28949443311206
H	-3.38067980076286	-1.34185118078984	-4.78961315322707
H	2.35248016920948	-1.83521825906897	-3.91023422584537
H	2.31292935038047	-1.53454850631953	1.35509514682302
H	-5.00767548041730	-2.40599567194216	1.07622945372777
H	5.29713000587679	-2.20215503283497	1.86615198190974
H	-3.65681795757486	-2.82115303280397	2.18405322976078
H	-3.43792033377115	-2.96583110619010	0.41575167809340
H	-2.41472749396395	-2.84802461267812	-3.27999233469233
H	3.36016636856558	-2.70074899445518	-1.91225982026974
H	3.88003093142476	-2.99028361582768	2.62385106201314
H	4.18958266464927	-3.18756271367323	0.86677472380941
H	-3.40533422675614	-2.70278760805786	-1.80145822933592
H	2.68640396305214	-3.74134374606076	-0.63258306474769

H -0.01290654413907 -3.84250060256894 -1.86774619415471

H -2.25128837606823 -4.07608645451210 -1.99520676504909

H 2.17072767734976 -3.99072841073570 -2.32439057603955

[Mg(^{Dipp}NacNac)(IMes)(CP)]

BP86/def2-SVP

Mg -0.10579386695025 -0.01429262084303 0.00863326810909

C -0.64881572582284 1.84168515728104 -0.97523526676981

P -1.07699720272570 3.13893802364620 -1.78534457352274

N 0.40498922455303 1.09228853206442 3.15489866027574

C -0.00971468593017 0.84279140767127 4.41806609305875

H 0.23749033556905 1.42977221391254 5.30853749955902

N -0.79698284271926 -0.25161910262639 4.39197894910534

C -0.86669222595468 -0.68646195634484 3.06390613477249

H -1.47736757591569 -1.55959971789639 2.80613653323713

C -0.11000902373485 0.14420458108549 2.24291468427797

C 1.25672285392902 2.22928440520155 2.86266901736770

C 0.65110195568705 3.46672820543988 2.53618494044748

C 1.50064548582004 4.57040527604488 2.32776919581583

H 1.04493785786178 5.54102896632524 2.07334062434762

C 2.90114828897280 4.47125445869931 2.43416257429996

C 3.45691517970802 3.21885582989514 2.75816117267176

H 4.55091659447537 3.11834012567793 2.84498309968783

C 2.65958584936747 2.07841159771110 2.97491585910785

C -0.84146129684613 3.60623270749344 2.37613255340269

H -1.15539572623447 3.19640128035378 1.39019813876583

H -1.14503200618160 4.67010531585230 2.40887557244447

H -1.40656047501565 3.06051348693029 3.15863781213954

C	3.78442359471849	5.66915418362074	2.17259417485537
H	4.02524498326364	5.75039388478375	1.09037472462444
H	4.74669044945159	5.59682640236454	2.71746586881512
H	3.28889205228642	6.61598370236893	2.46660227156625
C	3.29764197144135	0.74611308867015	3.28399108725273
H	2.75968589021379	0.19017818067980	4.07815186970304
H	4.34807188491696	0.87712995668101	3.60630348991987
H	3.30877953347870	0.09509532481481	2.38352108147141
C	-1.43780131300858	-0.85134816383651	5.53960318968126
C	-0.66859338429076	-1.66771015241777	6.40270349661186
C	-1.32316525187187	-2.24807359500726	7.50651578920676
H	-0.73913911704498	-2.88702850216438	8.18878638077443
C	-2.69299596245423	-2.04048027089847	7.75892007402045
C	-3.41718544265097	-1.22082540738384	6.87030787644481
H	-4.48923782255451	-1.04143964439944	7.05331226286770
C	-2.81881199767530	-0.61630457491906	5.74871929382657
C	0.80406754410812	-1.90915691020941	6.16318016073871
H	1.02175889156391	-2.11414241831610	5.09540299851507
H	1.16360340477090	-2.77021549280885	6.75824981953171
H	1.41791494411897	-1.02994583919663	6.45503185346996
C	-3.37825119810663	-2.70170706254917	8.93239747354749
H	-2.65708109779834	-2.98030683040259	9.72569791270879
H	-3.89558379425320	-3.63322093636914	8.61522304796745
H	-4.14801887929480	-2.04172292494471	9.38046186010015
C	-3.62856167691280	0.23977686128579	4.80499043230194
H	-3.10041922952066	1.17656447807857	4.53476745426428
H	-4.60179850982123	0.51130443470099	5.25641647282115
H	-3.83737423749597	-0.29319448477586	3.85251038393384
N	1.51117642656789	-0.97119563555823	-0.98699652316860
N	-1.46149639423565	-1.58325881963412	-0.52457750644151

C	-0.93586620816236	-4.74060004881173	2.24358037701012
C	2.61854906458947	-3.11472990797478	2.11942667845094
C	5.56941031717913	-0.41434448328924	0.20347527039943
C	-3.64647739667236	-3.53856649841522	1.81078419651481
C	4.87782201469520	-1.58000263554200	0.55907265797473
C	-4.76482077911445	-2.70128212369436	1.92973704788416
C	-1.35638538258972	-4.14559677418625	0.88560270903139
C	4.91506943485397	0.56419071035132	-0.55555838752843
C	-2.55237983485579	-3.19437612113185	0.99116728893795
C	2.87996528162867	2.84714433144672	-1.05436741335982
C	3.54111679868221	-1.79081092407111	0.16399928430820
C	2.83284463081023	-3.07762486072414	0.59178886392004
C	-4.79245543959356	-1.49295778940997	1.22167880589149
C	-3.99207265308038	1.42226655697759	0.57891668523294
C	3.57888905388178	0.40505508652661	-0.97835854031798
C	2.88091304478855	-0.79031539221463	-0.61713735892005
C	-2.59417026875016	-1.96180239786153	0.25647486031434
C	3.57140607416376	-4.34364721010331	0.11097328719242
C	-3.72692894352260	-1.09978804639372	0.38435261509240
C	-1.61688732588889	-5.28263934634161	-0.12714937168397
C	2.92938352951972	1.50828665686055	-1.81564623369433
C	-3.82923252032220	0.22228302523247	-0.37420644329496
C	3.62934695354024	1.69218729725596	-3.17924839288572
C	1.21046047815041	-1.61550086292927	-2.12706740686786
C	-1.31653783001551	-2.03685705233128	-1.78373609503205
C	-4.96564824376676	0.20054667964922	-1.41830712509871
C	-0.10710947077455	-1.97524465697437	-2.52446162835756
C	2.32889708145183	-2.04073773742878	-3.06503688548239
C	-2.50811457610483	-2.63380279953860	-2.52064717706510
H	-0.74215408322251	-3.95344504985999	3.00023655698695

H	3.58586761516130	-3.08874727085139	2.66492977362922
H	-0.00735706970972	-5.33780353347652	2.13090915348973
H	2.01263578930871	-2.24903818887848	2.45670875649406
H	-1.70661512487233	-5.42378092325249	2.65852186209624
H	2.08745753767806	-4.04084986760951	2.42267084759402
H	6.61596672786989	-0.27064430062976	0.51528379134286
H	-3.62377522712412	-4.48787161680687	2.36855305342540
H	5.39006859818617	-2.35157964214096	1.15673082055799
H	-5.61242304407807	-2.99320835660557	2.56959656126158
H	2.35525733589835	2.74418549527880	-0.08435179595741
H	-0.50684821515494	-3.54325340665559	0.49971994601718
H	5.45748440024881	1.48093383179376	-0.83774813499563
H	-3.17312761685970	1.45384111648119	1.32561457494007
H	3.89974241150042	3.24128088158370	-0.85459582211652
H	-4.95805858860081	1.38681246994316	1.12742490095184
H	4.57116803328991	-4.44314176502287	0.58423143155681
H	-5.67253499410522	-0.83520241763917	1.30870746127009
H	2.32707494377141	3.60551629315168	-1.64545253571697
H	-0.73105829214382	-5.94711347225926	-0.20921142519935
H	1.83011086508375	-3.07122389677842	0.11822098594848
H	2.99311151470346	-5.25637247100101	0.36607064948703
H	-3.95626613514304	2.37320658074854	0.00937111273627
H	-2.48003202634303	-5.90600663034416	0.19046916802633
H	1.87917178174151	1.20968422385905	-2.01154324746192
H	3.72411461666619	-4.33679868340564	-0.98746589488139
H	-1.83802551263291	-4.89304234645889	-1.13882192228392
H	-2.87530315449951	0.37382267362427	-0.92086475177984
H	4.67640451744221	2.04347196839756	-3.05793287031178
H	-5.96313015615653	0.11533989515244	-0.93602953535064
H	3.09237226145822	2.44713618669784	-3.79034564518585

H 3.66150698927531 0.74973184679654 -3.76311163154919
H 2.95298380695147 -2.82652827546156 -2.59078521047060
H -3.21662984863418 -3.13852403791598 -1.83737219014624
H -4.96036903416104 1.13583696415530 -2.01576957914576
H -4.86303486105440 -0.65156684546611 -2.12009858886474
H 3.01301481031116 -1.19871878751827 -3.28848868748200
H -3.06884215598662 -1.81406670429158 -3.01981306407845
H -0.17805829398900 -2.38316012024661 -3.54222926635438
H 1.93045458224016 -2.44355174152540 -4.01415266111423
H -2.19165982285862 -3.34491375632712 -3.30698043046596

[Mg(^{Dipp}NacNac)(I^{i}Pr)(CP)]

BP86/def2-SVP

Mg -0.17314300339193 -0.06086214155950 0.02212452053897
C -0.28303948423817 0.43727418233909 2.12842744381956
P -0.44005889348875 0.73027635787715 3.68109109811692
N -0.85621742575709 2.53781941778550 -2.08608490061707
C -0.14666631263762 1.98355543193389 -1.04784212066849
N 0.60886652120434 3.03047219559379 -0.57846796416457
C 0.38039809062615 4.19331680863343 -1.29990120613123
H 0.88612735852933 5.13902877947643 -1.08435258871323
C -0.55046260203710 3.88233389364193 -2.25577769823272
N -1.58561188077761 -1.54596963873013 -0.52179021458141
N 1.50749129640760 -1.34713832453530 -0.29477463649002
C 2.30948216037282 0.28019237719365 -4.16536141309596
C -4.62447253089142 -0.78759433610963 2.04874769005482
C -5.38641768754657 -0.74504384360609 -2.28585299686336
C 4.44660579723683 0.10536104439073 -2.10290544048799

C	-5.10034194240470	-0.29613418040858	-0.99074961134155
C	5.35651997827638	0.31884266689873	-1.05787822104187
C	2.23726289107579	-0.75792863551910	-3.03238844738891
C	-4.42779301071207	-1.50214384931086	-2.97419421430182
C	3.17855413351738	-0.46001106360181	-1.86516037059948
C	-2.82716587288922	-3.97297763801157	-3.72250444632873
C	-3.86567294718179	-0.57278304924219	-0.36740137050217
C	-3.64051617013541	-0.10949296371610	1.07180209730438
C	4.99342833316149	-0.02923416049261	0.24789188883234
C	3.43906711518053	0.29718007533464	2.91619237434633
C	-3.17974604426292	-1.81375852625419	-2.39824564822698
C	-2.88041267991568	-1.32310851819001	-1.08323120483919
C	2.81688309726215	-0.82239319786828	-0.52791220650278
C	-3.70058863974611	1.42338436258426	1.21651313283069
C	3.73520694451421	-0.59468667333146	0.54684551994068
C	2.48565160685189	-2.17902248088910	-3.58467444646291
C	-2.18974966982434	-2.66852006143692	-3.19918409735454
C	3.42537026706146	-0.94780567208609	2.00549749616774
C	-1.55990104645026	-1.87814581914184	-4.36364263216300
C	-1.27429276434556	-2.78255083022076	-0.07238599246119
C	1.31268443043346	-2.64761980555679	-0.04094851026402
C	4.40373224235824	-2.00657930358995	2.56234641189359
C	0.03733329503338	-3.24111573840631	0.19346254393024
C	-2.39096612887262	-3.79103824721936	0.15293465637136
C	2.47678371042342	-3.62909261621473	0.00778395730597
H	2.15927880176875	1.31228412147930	-3.78819213387221
H	-5.67435680692272	-0.47799318200037	1.85869437077787
H	1.52934241169899	0.07188924477940	-4.92678608739273
H	-4.58429877994550	-1.89323200977163	1.96900319499255
H	3.28363999588158	0.25365950136254	-4.69742590195240

H	-4.37698133516327	-0.51525497911471	3.09566838698882
H	-6.35831386357454	-0.52037837073691	-2.75265948697376
H	4.73479632485143	0.37419954498464	-3.13052442531268
H	-5.86170177580118	0.27653422667148	-0.43679170326883
H	6.34631138270516	0.75571565921917	-1.26300452049245
H	-3.30832874919504	-4.55421314337010	-2.90958894319716
H	1.20429342506944	-0.74430182320312	-2.62140425760798
H	-4.65960797241842	-1.87274591040105	-3.98577977306967
H	2.68770424601633	1.04310070161088	2.59476380986404
H	-3.60298395870394	-3.77643342168631	-4.49201225624631
H	4.43892939034125	0.78106653267676	2.93188664924077
H	-4.69987929451008	1.82691727748039	0.94554643165396
H	5.70890835779757	0.14010830773609	1.06850664556706
H	-2.05732961937916	-4.62048165965843	-4.19160541777270
H	1.77575157056672	-2.41572449541558	-4.40476205900251
H	-2.61907196314585	-0.42124968656914	1.37347510609794
H	-3.48183548302972	1.71980150980390	2.26242727236714
H	3.18457354042346	0.01545223845191	3.95867301388356
H	3.51642450880679	-2.26908709774605	-3.98835769403821
H	-1.36191731368437	-2.95165536166281	-2.51945076310132
H	-2.94245878592009	1.92011905814472	0.57673323026476
H	2.36374694430673	-2.94970664830634	-2.79888136346747
H	2.39865699351815	-1.36508521580930	2.04500840406905
H	-2.33359563352191	-1.52043380884146	-5.07590565068065
H	5.43579939102978	-1.60294712090302	2.63669798827722
H	-0.84928718489774	-2.51336304868874	-4.93310847881841
H	-1.00021901664649	-0.99555822273233	-3.99639186357958
H	-2.85229119800839	-4.11260562209708	-0.80236732640739
H	3.42297784224648	-3.18558318060320	-0.35122891871751
H	4.09797982291876	-2.31746544952420	3.58302824648078

H	4.44941899770790	-2.91361146149608	1.92878846680912
H	-3.20553643717757	-3.33843182490128	0.75354852201778
H	2.63359879297224	-3.98101910965466	1.04861045352197
H	0.08850848559466	-4.28547598393794	0.53347646238234
H	-2.01961947001422	-4.69153396281532	0.67570516266915
H	2.24753499128696	-4.52653591533184	-0.60058161380356
H	-1.01120931502790	4.50770167446002	-3.02592718457217
H	-0.42534447434366	1.42918548822478	-4.52974238841670
H	-1.45598446609883	2.87229146693708	-4.81486349250570
C	-1.44249738181039	1.84511729695583	-4.39433802194215
H	-2.14929712164848	1.23340274643979	-4.98783588029180
C	-1.86087696546327	1.83534487629265	-2.91810526878195
H	-1.86237825471556	0.79157086307949	-2.54434258278701
C	-3.25792539424390	2.43344509102769	-2.70741497180049
H	-3.31997963824388	3.47125586913606	-3.09705059931635
H	-4.00921157847266	1.81963687019926	-3.24127893434211
H	-3.53227759463092	2.44021800312839	-1.63550197091656
H	0.09728613441853	3.71152336614935	2.00706050342094
H	1.20386234577999	4.98617168935125	1.35836013617668
C	1.13213399950315	3.92458983382656	1.67577327513578
H	1.79758261169594	3.79042136191016	2.55139481055470
C	1.55566042024867	2.96107366828629	0.56134815549128
H	1.44652061849986	1.92920064034737	0.95564012499792
C	2.99522834940473	3.17984013639519	0.08305818547637
H	3.14426542196005	4.20720121808117	-0.31167695805519
H	3.69441415768355	3.04109524638092	0.93055989124175
H	3.27369401763619	2.45208610753469	-0.70442623954558

[Mg(^{Dipp}NacNac)(CP)]

BP86/def2-SVP

Mg	-0.00880998207329	-0.04093100756747	-0.02102067997000
C	0.01918576211556	-0.06670920768852	2.06080046781909
P	0.03926706524977	-0.07474147331483	3.64071758496821
N	1.49062642633420	-0.02662609334026	-1.40829728532362
N	-1.54585637540764	-0.04154462539892	-1.36685848016968
C	2.42131051829313	-0.00741010733260	-3.69493343125594
C	1.24574059825756	-0.02139191865556	-2.73284766969774
C	-0.05367472808599	-0.02700468789781	-3.30605372216569
C	-1.33695299746993	-0.03578315436418	-2.69751475567128
C	-2.53827982450284	-0.03790111221249	-3.62765093950706
C	2.84029371879405	-0.01967501470958	-0.91266742998348
C	3.47922736745359	1.22419980926813	-0.62499325955343
C	4.76666844875255	1.20353593246740	-0.05156299324557
C	5.41798390583742	-0.00392652870879	0.23570316513554
C	4.77848121568434	-1.21873778372762	-0.04490796310904
C	3.49148111076123	-1.25532820196528	-0.61940818727016
C	2.77390223273850	2.56034118285865	-0.86777475695421
C	2.25163660258297	3.14711943722207	0.46445604591481
C	3.65198020094126	3.58563766124152	-1.61498467013339
C	2.80971296420089	-2.60311454625314	-0.86678602181958
C	2.38908383967579	-3.25854570699385	0.46886066488377
C	3.67521758287514	-3.56811475381561	-1.70563900340174
C	-2.88229882771280	-0.04672998899252	-0.83634062348939
C	-3.52376464317093	1.19082369892095	-0.52919341293036
C	-4.79715100928301	1.15790285772651	0.07469696286440
C	-5.43195241493920	-0.05551931530012	0.37260389721131
C	-4.78869167991198	-1.26438765813718	0.07460426864055

C	-3.51519773073857	-1.28871410989977	-0.52960294427879
C	-2.83795504420700	2.53480771441299	-0.78442976474372
C	-2.33716111441162	3.14952079767225	0.54338176690654
C	-3.72828396038272	3.53311227373061	-1.55409912494824
C	-2.82204241556928	-2.62849187706045	-0.78709947652412
C	-2.32589516348598	-3.24816378925047	0.54017685265260
C	-3.70495369125218	-3.62640553830637	-1.56594094692824
H	3.05948682575616	0.88802048153106	-3.52390553421858
H	2.08750793195823	-0.00837674655352	-4.75251504690096
H	3.07850471626281	-0.88968043935587	-3.52764403841052
H	-0.06916416872319	-0.02303201199514	-4.40646655710795
H	-3.18085249084038	-0.92668378253994	-3.43940347699815
H	-2.23279020590107	-0.03907648079478	-4.69384256875780
H	-3.18136781486806	0.85116398711628	-3.44210397410014
H	5.27033395123241	2.15572359082099	0.18446090460438
H	6.42402952769960	0.00227816419627	0.68641144381604
H	5.29089314158694	-2.16514322122552	0.19677971737807
H	1.88817936390377	2.36052558684167	-1.50988329310634
H	1.59742182049238	2.42532730549010	1.00617165561809
H	1.66783250246755	4.07917934233152	0.28700301202738
H	3.09510191230027	3.39517724177320	1.14828577762073
H	4.52644922264903	3.91030998807388	-1.00734149773664
H	3.06257983305652	4.49930106258227	-1.85406299590980
H	4.04212699949529	3.17227413669274	-2.57119752900331
H	1.88227832798059	-2.40674515460342	-1.44854152727319
H	3.27996949389580	-3.50507841708147	1.09033356948622
H	1.82806148427080	-4.20358896532370	0.28757010099042
H	1.74428453281744	-2.58172757384303	1.07496197192126
H	3.98317794735169	-3.11033784425803	-2.67171030791075
H	3.10908134685070	-4.49889519738245	-1.93520459556578

H	4.60142462442645	-3.87030402838238	-1.16734712476574
H	-5.30199690555378	2.10551235094334	0.32677558985147
H	-6.42760332070659	-0.05902506752832	0.84590955927188
H	-5.28698439058101	-2.21548901993401	0.32669451415994
H	-1.94391075516010	2.34028651274935	-1.41661676639098
H	-3.19012089681031	3.39326128429182	1.21704774528122
H	-1.76889130650517	4.08932593502455	0.35688918091284
H	-1.67411225639894	2.44669514611055	1.09876439217923
H	-4.09685855656555	3.10163373097947	-2.51087451482744
H	-3.15641520573878	4.45726480434104	-1.79569133869791
H	-4.61778682782724	3.84325567996654	-0.96097886746827
H	-1.92545476599255	-2.42726442930374	-1.41367423091841
H	-4.59605542405194	-3.94224335479452	-0.97826947304196
H	-3.12861728399183	-4.54736651657452	-1.80911911532249
H	-4.07071130914309	-3.19196697453778	-2.52247944987344
H	-1.75690925330379	-4.18716133733910	0.35187400986433
H	-3.18130924326044	-3.49537508360746	1.20948064745666
H	-1.66520108247363	-2.54762784952563	1.10107589794408

[Mg(^{Dipp}NacNac)(dioxane)(OSi^{i}Pr$_3$)]

BP86/def2-SVP

Mg	-0.00032121623149	0.00615431674373	-0.00134167335701
O	0.08044934205169	0.01315153427310	1.88219913288491
Si	0.19442218785863	-0.52134039531950	3.45065270724970
C	2.12136183010600	-2.56112716079453	2.75724998575756
C	-2.56793966673972	-1.45286064463285	3.21036096490130
C	1.92734038467544	-1.35300096751847	3.69133895452894
C	-1.20226781519680	-1.81098075277771	3.82626737845408

C	1.27625380629945	1.91490642736759	4.61460104158135
C	-1.25088947255578	1.72765765440089	4.61953012759403
C	0.07743987520579	0.95131633752759	4.70381749481578
C	2.32003869669093	-1.70931041473101	5.13802724718395
C	-1.35782502338077	-2.16270068424786	5.31943654881541
H	1.93132539828365	-2.29867219797860	1.69584275287560
H	-2.49271250390675	-1.19535928902492	2.13507789778382
H	3.15986170978840	-2.95786130357088	2.81043781378988
H	1.44152965205291	-3.39985949136224	3.02568984257155
H	-3.28650405010987	-2.29788523779582	3.30211994879962
H	2.62961571837239	-0.55868334575623	3.34264872255763
H	1.33103311536940	2.40998031542749	3.62088226105792
H	-1.39664241813541	2.18177405547254	3.61615039578861
H	-0.84226740730252	-2.72967179239123	3.30246713184314
H	-3.03230855699334	-0.58425511687576	3.72288089801945
H	2.24773843807427	1.40219243912244	4.77076497941273
H	-2.13042780007626	1.07907376097929	4.80788130078805
H	1.20651896192701	2.72589922411638	5.37458763152455
H	-1.28831358584769	2.55760275586562	5.36113768887623
H	3.37749833804037	-2.05336329836714	5.19395604940180
H	1.69867957880419	-2.53765587480736	5.54064638067837
H	0.11490547388410	0.46642816126422	5.70797016790923
H	-2.10403403191237	-2.97616050499578	5.46760235696684
H	2.21671965312042	-0.85329654580816	5.83717516968982
H	-1.71605343292151	-1.29034137152663	5.90845194249827
H	-0.40921815971507	-2.50154033353159	5.78283457568904
O	-0.01297466972193	2.14514029018706	-0.31296796247224
H	0.05899386754289	2.13037836436898	-2.37246358510862
H	-0.04015876874978	2.39613909281437	1.71676967009178
C	-0.18189766402546	2.85066385801709	-1.56578626108504

C	-0.24301238852541	3.02066918544563	0.82402915899391
H	-1.25014890045933	3.15357599642002	-1.66082036721688
H	-1.31307475422587	3.32095867974830	0.81749559304789
H	1.78909407955961	3.76380904871047	-1.59605919751408
H	1.71777940422168	3.96765458628341	0.85253990216241
C	0.71669751428666	4.08191223906268	-1.59761172168324
C	0.64462519162495	4.25433486538888	0.73277133529058
H	0.52857251356296	4.66238154008027	-2.52423884840915
H	0.38537660544640	4.96001976947039	1.54773568598458
O	0.44997510696264	4.94410719583524	-0.49955407339475
N	1.46633632385201	-0.62410289186473	-1.37828829806118
N	-1.62915176920009	-0.56526371434171	-1.21946102063394
C	4.38754791643069	2.08588135118986	-1.24988612839158
C	-3.82751860837553	2.14627100549643	1.01641532149349
C	-4.45848621631468	2.23617896225513	-1.43815849352924
C	3.68424736265291	1.72108307722769	1.16546523699632
C	2.67852648868185	-4.27989849388917	-3.00083872626900
C	-2.66561860009571	-4.36124287486398	-2.11548695601658
C	-5.48515409459809	-1.79588155086075	0.16148130830928
C	-1.92816555077330	-4.31512304480361	0.31264457131297
C	1.61579284906896	-4.50254082205615	-0.71862105325950
C	5.30849582556603	-2.11381596743656	-0.24166437935330
C	4.35362055977108	-2.96701133615601	-0.80705165934224
C	-4.51642794629346	-2.74323906953774	-0.18716622572555
C	-3.61649976284029	1.50480467788245	-0.37115446845487
C	-5.16257741735465	-0.43617524973761	0.09311843407101
C	2.24611296612646	-0.52598694888579	-3.73314704725127
C	2.09666718537599	-3.50963861252676	-1.79631108337992
C	4.99765398223191	-0.75994163310429	-0.07529430648553
C	-2.61971490803915	-0.62928067495124	-3.49526341643485

C	-2.21764913769097	-3.46398269781049	-0.94208770646048
C	3.49976469232613	1.26176402609500	-0.29304369575557
C	3.08721959162002	-2.49896363027068	-1.21110581269723
C	3.74592828675670	-0.23747705841607	-0.45929642617782
C	-3.89491645438891	0.00128576675837	-0.34239801909706
C	-3.23349994190170	-2.36259022252959	-0.62928747051165
C	-0.17103151378749	-0.29438469830280	-3.17449687546890
C	2.77236359510396	-1.11320022063829	-1.03071900232087
C	-1.43878481551362	-0.48200328312169	-2.54878404248450
C	1.15129542000793	-0.47455051200658	-2.67892790028799
C	-2.92471920712722	-0.97092233929520	-0.73804476527840
H	4.23812660565856	1.78586002439705	-2.30783181798095
H	4.15905469786692	3.17055366987132	-1.16879849476450
H	5.46575403717155	1.95579479605193	-1.01708688984816
H	-3.20586476249490	1.65554623860524	1.79207107342409
H	-3.56998997690408	3.22687268364226	0.99507950748537
H	-4.88644815190260	2.07641963831668	1.34138952947583
H	-5.54394640773147	2.15792812677156	-1.21706982830803
H	-4.20227937182963	3.31650250280062	-1.47256452403325
H	-4.29544361042174	1.81834040125920	-2.45206699327187
H	4.71953485489184	1.54485230212635	1.52419547815046
H	3.49177822469119	2.81057828738213	1.26250306169462
H	2.99056312056963	1.18717930141304	1.84513603409780
H	3.50719186320701	-4.95421572966479	-2.69737555294184
H	1.89797159985860	-4.91420641882613	-3.47038427729478
H	3.07667348746268	-3.60025840911199	-3.78116506430099
H	-2.85735144210347	-3.77592517745391	-3.03841641612707
H	-1.88548331849592	-5.11412359916740	-2.35203687957820
H	-3.59789267182029	-4.91499260253762	-1.87460725912947
H	-6.48261979153482	-2.11673423941715	0.49901996691247

H	-2.84591192205635	-4.81596905023868	0.68536692601944
H	-1.18675672654921	-5.10781305101351	0.08763726810578
H	-1.52910839433369	-3.69663342548766	1.14105575334165
H	1.12535527410738	-3.98604229845607	0.12864413971016
H	0.89091987757349	-5.22658506747401	-1.14700336647538
H	2.46525019975322	-5.08487954285434	-0.30465925731252
H	6.29119774429390	-2.50252794962858	0.06646935433940
H	4.59631303410629	-4.03336236938327	-0.93719144296033
H	-4.76009481806413	-3.81518921743915	-0.11061357908188
H	-2.54834346803265	1.62993087295442	-0.64092033546355
H	-5.91581746665491	0.31183981119450	0.38619968456059
H	2.53223548805504	-1.57397181490449	-3.95464306506124
H	1.90709550822463	-0.06698365019316	-4.68073372371837
H	3.16682040542265	-0.01629809696259	-3.38842945197810
H	1.20762100352487	-2.95056901645139	-2.15162134275470
H	5.75084488881253	-0.08413513705223	0.35929685869242
H	-3.50235084213687	-0.06121375650722	-3.14458313436276
H	-2.35864794700164	-0.30382767700667	-4.51923673855401
H	-2.93944886040498	-1.69007923997907	-3.54566216928818
H	-1.26991139229776	-2.97033374976525	-1.24023559842949
H	2.44432090269190	1.45541096779088	-0.57468065176279
H	-0.22545273353024	-0.14738562767759	-4.26225182190112

[Mg(^{Dipp}NacNac)(OSi^{i}Pr$_3$)]

BP86/def2-SVP

Mg	0.02020324687358	0.00141080240531	-0.03152196820730
O	0.05427515242043	-0.03700092266954	1.81209828760307
Si	0.13880415635795	-0.24041181759649	3.46185160855431

C	1.08277468723366	-2.51331598319891	5.13455585940474
C	-2.73053176388636	0.21088277612361	3.38059889367423
C	1.24005230652113	-1.80396020563425	3.77469045730408
C	-1.64608483923601	-0.57591169943531	4.14414352293624
C	0.12227187373346	2.61596138332971	3.97354068963468
C	1.42096423649445	1.25293119871161	5.67472384346102
C	0.96507411406137	1.34490496859082	4.20408532486727
C	2.72640997904939	-1.54351704160974	3.45408643206710
C	-1.81754258436652	-0.38500220924930	5.66589464112545
H	0.03062076847081	-2.81688567231962	5.33160296361683
H	-2.69027204987535	0.02586797095977	2.28465387047728
H	1.70382390715169	-3.44058378494926	5.17532313566308
H	1.40948842609660	-1.87111581726391	5.98435186023615
H	-3.75177321983357	-0.07267999340827	3.73252402563344
H	0.85194837112512	-2.50765271081336	2.99541105438374
H	-0.79753260151659	2.61955231054208	4.60130022762396
H	0.56084049568483	1.13206297757043	6.37041583451809
H	-1.81364160984177	-1.66045908514844	3.92248125023859
H	-2.62737782358285	1.31012436475698	3.53472579697863
H	-0.20126046843551	2.71340733954418	2.91383006568340
H	2.11162513793160	0.39945186716917	5.85296093864004
H	0.69738718864501	3.53781415379719	4.23481939157060
H	1.96010205548756	2.18136486527069	5.98525181797722
H	3.31371448168296	-2.49312858707173	3.46091946534300
H	3.19700657522173	-0.86889901210994	4.20640251078464
H	1.88546113436773	1.44393144226378	3.57690314195084
H	-2.83089332746059	-0.71725648547851	5.99842696980160
H	2.86717214878927	-1.07318922713765	2.45513843139010
H	-1.72251515975490	0.68671826507189	5.95319179263801
H	-1.07334048722858	-0.95435175608923	6.26397549498712

N	-1.53018320619728	-0.02132072281024	-1.37621077211742
N	1.50214828365284	0.08698970955785	-1.44968434867089
C	-2.54137146068739	-0.03990829937837	-3.63082201536398
C	-1.33442081893996	0.01164688762573	-2.70903203572071
C	-0.06100425084135	0.09271842248927	-3.33088596686187
C	1.24204019658530	0.13551649473778	-2.77060363152751
C	2.40260108947377	0.24650283148485	-3.74386783754988
C	-2.86690022610378	-0.07993524135895	-0.84958411259102
C	-3.43104002071892	-1.34438781899354	-0.50427622770321
C	-4.70949483265546	-1.37303578951039	0.08948440190933
C	-5.42475096514182	-0.19364556619588	0.33668982685203
C	-4.86085716149732	1.04235563407237	-0.00738921806396
C	-3.58578806750702	1.12792455400721	-0.60240661298359
C	-2.66647522686723	-2.65061075853352	-0.72889669713325
C	-3.50168931500304	-3.71761379997758	-1.46776959269199
C	-2.11839550851827	-3.20323369563463	0.60695364881184
C	-2.99454323031914	2.50160973786915	-0.92925949795514
C	-2.59114351917439	3.25149248618146	0.35997550864611
C	-3.93788692007179	3.36788661720070	-1.79227521772469
C	2.85890528524881	0.09920668415393	-0.97495221450510
C	3.56074161718167	-1.13254283862665	-0.80849907520532
C	4.84047774261108	-1.10219641864711	-0.21805202100266
C	5.42895334292401	0.10300578677302	0.18910718652765
C	4.74025184033984	1.30858631002027	-0.00234266411085
C	3.45775530622721	1.33514254379179	-0.58733195436281
C	2.95355156848034	-2.47292822714309	-1.23258754471063
C	2.53480460474393	-3.31623241224679	-0.00871470390948
C	3.89153408491767	-3.27891771434689	-2.15766477056401
C	2.72915665595392	2.66822011219698	-0.77034690752347
C	3.59159327115976	3.73018815633749	-1.48492927966650

C	2.20212745465955	3.20364468851073	0.57963762857805
H	-3.16052451378222	-0.94037493819731	-3.42061980103036
H	-2.24074233623601	-0.05605794460055	-4.69823842303803
H	-3.20553906822235	0.83690574155974	-3.46296970517998
H	-0.08958369097584	0.12768852515308	-4.43036401610497
H	3.01076011012669	1.15326656678645	-3.52816835847879
H	2.05308364055480	0.29759809078272	-4.79501122039604
H	3.09425424619751	-0.61919004378197	-3.64201878585984
H	-5.15504173219711	-2.34213986685231	0.36985168692607
H	-6.42246794579925	-0.23733283220996	0.80344140032984
H	-5.42497058184086	1.96744711357894	0.19947459252701
H	-1.79068837954282	-2.41923317154389	-1.37445749871715
H	-4.36561051815372	-4.06739164451016	-0.85889387834546
H	-2.87680868746675	-4.61064013942424	-1.69429906715096
H	-3.90350564032934	-3.32918639831754	-2.42961428450529
H	-1.47756560588633	-2.45526851872995	1.12867635310156
H	-1.51209277533536	-4.12223026947486	0.43739683910522
H	-2.94879842920060	-3.46323102154838	1.30199806011225
H	-2.06805184765090	2.33714679888653	-1.52106788013414
H	-3.47855992152437	3.45039473422894	1.00253273433135
H	-2.12138376206026	4.23165366069184	0.11723337500099
H	-1.87019750909863	2.66297234799287	0.97100028677916
H	-4.24746338643098	2.84025949913685	-2.72171216706902
H	-3.43271227446214	4.31402283936762	-2.09041934915048
H	-4.86390052535537	3.64869821889308	-1.24227482466046
H	5.39015571538803	-2.04680863279902	-0.06794662454298
H	6.42862772086183	0.10274746274443	0.65371386718506
H	5.21130769752581	2.25417927218062	0.31422935185924
H	2.02981705247330	-2.25684559127737	-1.81214729851624
H	3.41178744658866	-3.56550667303155	0.62986460595839

H	2.06482750530290	-4.27289410194358	-0.33089751167913
H	1.80727599241029	-2.77275049985149	0.63474082915283
H	4.20615516061647	-2.68325966823062	-3.04346594249902
H	3.37960592768259	-4.19576895481790	-2.52714061492947
H	4.81513861139674	-3.60755983126922	-1.63060336802947
H	1.84505939157524	2.47747359754327	-1.41757516051450
H	4.46666551369409	4.04102978859584	-0.87081882649875
H	2.99053218845317	4.64551913060791	-1.68561189384888
H	3.98006136339323	3.35656743381678	-2.45800971842422
H	1.55157558842760	2.45921275743834	1.09278401704860
H	1.61233842805408	4.13719064117524	0.43384082631710
H	3.04175770853091	3.43546349272007	1.27374647990255

TS1

BP86/def2-SVP

P	0.60852981993776	3.10283222228875	1.32023886475340
C	-0.04810040284993	4.35548225276642	2.24886128676215
O	-0.84226702331127	4.56395447963591	3.28269135347139
Si	-1.05082420093927	6.26694062610474	3.78959730805149
C	-2.11549122108035	8.55402324595105	2.30044215815325
C	1.79483024320033	6.21274273193543	4.38042945113257
C	-2.15744204928137	7.01611568740729	2.41414489624177
C	0.66478254389975	7.12030053095155	3.85836082294590
C	-1.07788178953951	5.37632570979604	6.54392149144560
C	-2.58968859092354	7.35268146862881	6.00142873879415
C	-1.94922700253829	6.05294866030610	5.46508567001067
C	0.63965112104384	8.45746059815781	4.63569475134424
H	-1.08412151964524	8.93540304148144	2.13377014845046

H	1.88274310768606	5.28647053977406	3.77403949041103
H	-2.73200284104784	8.89747699434044	1.43587691850879
H	-2.52250509504970	9.05808651692058	3.20680623885584
H	2.77378955475651	6.74653517671836	4.32919261890172
H	-1.68042906568613	6.60142329528180	1.49122631710742
H	-0.25998744552105	6.04902438776604	6.89052227052333
H	-1.82116210687182	8.10841800470624	6.27599053794083
H	0.88701990674994	7.34407742189277	2.78733098428567
H	1.63676011079285	5.91859449253680	5.44202259994141
H	-0.60748412605599	4.43556147553849	6.18148830682573
H	-3.27633186714577	7.82903772727662	5.26580164205320
H	-1.68953775238040	5.12208235717573	7.44173790585023
H	-3.18683578997063	7.14399313005648	6.92060208203818
H	-2.77995356809643	5.34896769923550	5.21669999136701
H	1.61048399422438	8.99346639139851	4.51099778730357
H	0.50313166287555	8.29234748667833	5.72881706266633
H	-0.16344697425075	9.14902602284950	4.29858619176177
Mg	-0.09060687603549	1.00627480870979	-0.28125416886241
Mg	0.56120810110170	1.13568781235121	3.12581690737161
N	-0.22164725420655	2.04789892163165	-2.13653989392655
N	-0.28754184485505	-0.87244531626244	-1.23867854879437
N	2.35596520373077	1.18611154137841	4.31149311633966
N	-0.69663939948248	0.74709580947085	4.78881022533247
C	-0.66054051234874	2.26781402489931	-4.56846953211399
C	-0.56844817062765	1.44474276546131	-3.29150267233211
C	-0.82947380087687	0.06003334420824	-3.43766355203170
C	-0.64134891079203	-1.01150791390986	-2.53434469041500
C	-0.80784576516060	-2.40600884745454	-3.12178678222704
C	0.12475477848996	3.44553687764569	-2.20286107229321
C	-0.88215592300630	4.44573060814954	-2.06226372623635

C	-0.49131543053482	5.80016014899018	-2.04790676785936
C	0.85168738401906	6.17441478470073	-2.18911037635551
C	1.82655987999103	5.18525244216878	-2.37240834709006
C	1.48931250007728	3.81762817299721	-2.39574373503062
C	-2.36645208105082	4.09188436162484	-1.93509999096599
C	-2.91209040432915	4.43826084493807	-0.53587737381779
C	-3.23188424726600	4.76010480106405	-3.02634669497537
C	2.58600405871500	2.77777608795243	-2.64470808925839
C	3.58153203550183	2.70146683509659	-1.46990422155650
C	3.33289330708901	3.02528857805937	-3.97396757861195
C	0.01616863361469	-2.07443934521010	-0.50973003426506
C	-1.01619325284546	-2.77798158141928	0.17913716326466
C	-0.67378382230951	-3.93377851074977	0.91030633785812
C	0.64430490916742	-4.40852485041141	0.95022411868981
C	1.64633762325457	-3.72231186888163	0.25085422832451
C	1.36045869817466	-2.55482492905072	-0.48572844593926
C	-2.47413720991900	-2.31665982956202	0.13595425690912
C	-2.90565122619833	-1.72563919908791	1.49121670958766
C	-3.44553014778715	-3.43390121351085	-0.30408808052552
C	2.48509427862943	-1.84884840471803	-1.24846400230336
C	3.48973924040848	-1.18122164514863	-0.28774821375613
C	3.21138942645224	-2.79229330164905	-2.23111928495983
C	3.43800459050691	1.92341056902240	6.44138456672651
C	2.22186895253760	1.61307865415016	5.57588958358666
C	0.97525512249420	1.81328396911022	6.23969628341903
C	-0.32447600527771	1.33204121368918	5.95142038576666
C	-1.34396354454393	1.46614656330809	7.07520746868786
C	3.65918717448954	0.88549106229724	3.77653794677784
C	4.06384420488648	-0.48628771754851	3.70003885301652
C	5.30651150594568	-0.80161864567695	3.11606642677203

C	6.15401808665967	0.19604093932094	2.61659395933535
C	5.76380584265645	1.53513213727040	2.71614642309729
C	4.53293576218127	1.91127101925926	3.29612330076630
C	3.21410621507118	-1.59956978339334	4.31548384428179
C	3.53601703902122	-1.76414219443396	5.81884114900969
C	3.34823415743571	-2.95333514782151	3.59573701314963
C	4.23275822136158	3.40640288050043	3.42142704678303
C	4.16390253819546	4.10340669207775	2.04741918892198
C	5.27258185785706	4.12365509287134	4.31570584463674
C	-1.92323098422348	-0.00790570595524	4.80233146989997
C	-1.89824604204502	-1.36578679470585	5.25743124065891
C	-3.10128456160652	-2.09929285339630	5.29561188201857
C	-4.31631203033579	-1.53463381960661	4.88794417230819
C	-4.33130896984853	-0.21268716511242	4.42934709338023
C	-3.15895685537196	0.57171540233109	4.38481326355100
C	-0.61161750440743	-2.05805882255515	5.71310919061342
C	-0.30185976709217	-3.29402030325683	4.84324742227541
C	-0.65114010328145	-2.44456209096304	7.20854941990342
C	-3.27330145136677	2.01766778529326	3.90334250836330
C	-3.60396460169971	2.08183709822976	2.39937874994312
H	-1.41180616274189	3.08152392388188	-4.47489661296819
H	-0.93256117359490	1.63453285097970	-5.43690321331387
H	0.30805663036948	2.76780251036868	-4.78754941718774
H	-1.13679110479883	-0.24324718029459	-4.44879109173327
H	0.16095733743923	-2.95292278306087	-3.11429998394398
H	-1.17352316096549	-2.35930026221346	-4.16736811885285
H	-1.51275617396809	-3.02199977010635	-2.52406618708760
H	-1.25957735293054	6.58309881546313	-1.92689042452929
H	1.13772885312757	7.23968571987754	-2.16802060316589
H	2.87995203619198	5.48575180254296	-2.50679925528400

H	-2.45833558642904	2.99121965643941	-2.06278871246277
H	-2.28766765765592	3.98204255019002	0.26100779570153
H	-3.96000288106007	4.07890163869499	-0.41698749513745
H	-2.91379273625860	5.53969627289417	-0.37048917578623
H	-3.24873118443745	5.86839096158673	-2.91575457877693
H	-4.28494671244255	4.40313154682589	-2.96080033242581
H	-2.86083372105318	4.53413382643901	-4.05095102755679
H	2.09107293184931	1.78540082252444	-2.72718315509820
H	4.11147943234933	3.67005588183372	-1.32433305767833
H	4.35059237484972	1.91734413949334	-1.65279161597612
H	3.06710109674020	2.46719695609537	-0.51284563082680
H	2.63379760740165	3.06847522852547	-4.83880995657663
H	4.06534554874794	2.20894989099232	-4.16813314726926
H	3.90053640058679	3.98308115218004	-3.95458450291541
H	-1.46200713822012	-4.48228882489160	1.45301403513660
H	0.88994042565756	-5.32010993150442	1.51979587481826
H	2.67907560891121	-4.11073823612774	0.26928032772779
H	-2.54416759455870	-1.50317840459060	-0.61893390543384
H	-2.86616114289488	-2.49030106316730	2.29959199600880
H	-3.94654867324986	-1.33601115056913	1.44563592136345
H	-2.24127672557972	-0.88814315359769	1.80075784217263
H	-3.14727936858358	-3.88291145713940	-1.27752129490562
H	-4.47430951409757	-3.02487842578358	-0.42037223799552
H	-3.50224685644270	-4.25877061699270	0.44220654273170
H	2.01854761923305	-1.04095697285008	-1.85286585192948
H	2.99018965255476	-0.44998568843304	0.38789302841140
H	4.27966530400448	-0.63662722829822	-0.85263706976809
H	3.99766322583142	-1.93129887510065	0.35957864162454
H	3.75666223018885	-3.60527988693488	-1.70001029294098
H	3.96181744892312	-2.22718214889213	-2.82943178082212

H	2.50325893745019	-3.27361084980796	-2.94209457898618
H	3.30731325764073	1.51305176956232	7.46532043453686
H	3.55323351560889	3.02591913110076	6.54679916591869
H	4.37713475682323	1.52215096934646	6.01247231131944
H	1.07341310715872	2.27234815078644	7.23489577652263
H	-2.25896958976823	1.98602283466767	6.71709718375114
H	-0.92606902564483	2.02890673591067	7.93462937667230
H	-1.67694817644532	0.47069632619249	7.43934017774968
H	5.62461822918973	-1.85452178613937	3.05765082691157
H	7.12197579207770	-0.07236971063278	2.16143028574046
H	6.43964708597120	2.32206272659189	2.34191565324613
H	2.14920502378814	-1.28077137473581	4.24205026796016
H	4.60040074502585	-2.06170762462703	5.95856127053321
H	2.89706407902765	-2.55342847363625	6.27611804781092
H	3.37231446533692	-0.82365288439788	6.38582061051489
H	3.16472976556666	-2.85384538362863	2.50413792343392
H	2.60475061195123	-3.67577139369072	3.99721496466419
H	4.35448015199858	-3.40925446265273	3.74001035399392
H	3.23479804379614	3.51158463520980	3.89852131515174
H	5.13447842672444	4.02674043043465	1.50641007023610
H	3.92834849212204	5.18392645271987	2.16963948896120
H	3.36627994788344	3.66118416791859	1.41413900016911
H	5.40426883991212	3.62420270671672	5.29901014044414
H	4.96069333351740	5.17585969038990	4.50426808446696
H	6.27141034429737	4.15714869266668	3.82478177990720
H	-3.08493848830360	-3.14099988463708	5.65717188535410
H	-5.24814478170682	-2.12396197495777	4.92409468585096
H	-5.28715480739909	0.23214392883015	4.10517357868565
H	0.22139536822847	-1.33569302987665	5.57884802309256
H	-1.09830280259745	-4.06810780882380	4.93212699299922

H	0.65377002327811	-3.76556382189911	5.16532269474279
H	-0.20947668987731	-3.02722703935669	3.76754559404865
H	-0.84407164068125	-1.56388462078459	7.85929918359943
H	0.32165329509714	-2.88619531116201	7.52349805988002
H	-1.44415870064728	-3.19869388201384	7.41690023937398
H	-2.28792177525707	2.51322856782612	4.04286333801263
H	-2.80072958104833	1.61076599076774	1.78923293285819
H	-3.69735992207927	3.13892051044355	2.06749802003878
H	-4.56312367001828	1.56434657403841	2.16740023471892
H	-5.34524380334393	2.41952272092147	4.58233206018981
C	-4.31447213828784	2.81982974038263	4.71456265372646
H	-4.08746404387043	2.80664574685624	5.80391163423325
H	-4.32994644494559	3.88098622862907	4.38035734314690
H	-3.65480130632535	5.38598479899668	2.47496998959259
C	-3.60806836994519	6.49769901278141	2.46763310802094
H	-4.18704220124831	6.84673435063987	1.58070005468223
H	-4.14732968529189	6.86458311011610	3.37093584824942

B (Intermediate)

BP86/def2-SVP

P	0.50331809979286	1.23530381952235	2.08070337020174
C	-0.48496564151192	2.57544533421002	1.55516930263933
O	-0.91693399976704	3.53920682887781	2.41163699618707
Si	-1.69243269079734	5.05103511918937	2.01279888934147
C	-4.06855881041185	5.71207157932726	0.38486462075287
C	0.97367142287445	5.90160941386575	1.30718710702122
C	-3.37535168283763	4.59818242160560	1.19587092858153
C	-0.50000031232373	6.02157385816023	0.86604137372575

C	-0.68702097646688	6.02854355739731	4.56558564082517
C	-2.83597264824818	7.10527898816288	3.74322554217066
C	-1.97674387171188	5.82215869381027	3.74774050841972
C	-4.34767428714067	3.98567450316362	2.22595501380472
C	-0.87817121289377	7.50366253686928	0.65246405358732
H	-3.42834225247052	6.10323610448759	-0.43443735017492
H	1.29094748068374	4.84210863281387	1.41630857141180
H	-5.00556252760033	5.32856444333974	-0.08522971357181
H	-4.36012502914788	6.57606116618289	1.02452430654958
H	1.64093301147073	6.37083060070997	0.54814368464539
H	-3.08948057528902	3.78576435589762	0.48145122610415
H	-0.07760875053744	6.86814967856295	4.16145320634022
H	-2.31885416941762	7.95171127515776	3.23863929757181
H	-0.58619268012595	5.50302192711552	-0.11799522255851
H	1.15446495114135	6.41563927276444	2.27723516784527
H	-0.04682656622870	5.11908846843306	4.56874424156113
H	-3.81546908626545	6.96440005720956	3.23501720454556
H	-0.92516868593541	6.28169141358413	5.62664385500439
H	-3.05401954066395	7.43449400139018	4.78719698373148
H	-5.26673529235557	3.60288829359601	1.72266335144905
H	-4.68278711161480	4.73986485955558	2.97405057443569
H	-2.57005458640429	5.03124774740320	4.26471812538164
H	-0.23149757969228	7.95861997268880	-0.13452191641305
H	-3.89208875216117	3.13676511881011	2.78103052377478
H	-0.72377725054779	8.10078017863963	1.57997958184251
H	-1.93481897224968	7.64705850687235	0.33680534771654
Mg	-0.30197470894382	1.54620707213088	-0.39240326278205
Mg	1.10470105171851	1.03686931333673	4.55487316214399
N	0.50800668203117	2.26677491003482	-2.22025138918674
N	-1.11799107384382	-0.18739724259093	-1.29844951985520

N	2.94107894112692	1.26400044697014	5.52425637595762
N	0.19420602937014	0.08341918854321	6.16864431689442
C	0.39912677633730	1.58697813852769	-3.37831924490910
C	-0.39328091878763	0.42680629121253	-3.56016955942327
C	-1.09188828789977	-0.38444734130058	-2.63284897472030
C	-1.80542811298725	-1.58687221096402	-3.24482841552574
C	1.19056474084786	3.52582308643540	-2.23627982557302
C	0.58238129820152	4.66186712395257	-2.86001106816154
C	1.27246014300394	5.89091931158277	-2.83481238794597
C	2.53061185145674	6.01238761628588	-2.22853098349629
C	3.11643072377722	4.89333842536517	-1.62156968081028
C	2.46716142710533	3.64246781849630	-1.60644958223242
C	-0.79215960069462	4.58450998720954	-3.53783352139178
C	-1.91653764332293	5.07621195654061	-2.60378497291566
C	-0.84729274836431	5.33422669359115	-4.88585212718477
C	-1.60267926708489	-1.27161309887127	-0.49480919903701
C	-2.85502073991895	-1.15671594458283	0.17922080221083
C	-3.32474250051806	-2.25832726046307	0.92337973118333
C	-2.58705864260692	-3.44620375181555	1.01369676828658
C	-1.33944567111126	-3.53450863221094	0.38192410010591
C	-0.81816031396650	-2.46194576308869	-0.36817357291176
C	-3.69337451300818	0.11955049023489	0.07078205577933
C	-4.63513245693147	0.33411203707870	1.26879616877982
C	-4.49222420578641	0.18424429786952	-1.25088613387722
C	0.58434289571822	-2.58143357400937	-0.97640813381993
C	1.66182982606795	-2.25791663343828	0.08228795881729
C	0.86208842299192	-3.95140403156569	-1.62966858235556
C	4.54049727654671	0.93586846116056	7.39909233908653
C	3.14654961388297	0.84910638959831	6.79125761895141
C	2.15395423477931	0.27107498598289	7.62416248867265

C	0.81923978432357	-0.11505892423520	7.34674135769159
C	0.07720019987679	-0.82835659459508	8.46608695273493
C	4.06636846022981	1.77600514911568	4.79026321469907
C	4.96700932881958	0.87632042490367	4.14538585157614
C	6.07353674839086	1.41000650710278	3.45404307439939
C	6.29349750199344	2.79139300458000	3.38898275347107
C	5.38189849851951	3.66746540410631	3.99463992107022
C	4.25495979101353	3.18891579210621	4.69405294993304
C	4.73405905643144	-0.63680748715719	4.13954834672209
C	5.99019997563417	-1.45173234323277	4.51296873989791
C	4.17794533151729	-1.08964590528358	2.77071110130889
C	3.26689123596170	4.16976283698115	5.33566228315692
C	3.20509590086890	5.52806346894265	4.61258097673126
C	3.53459122004996	4.39477571533028	6.84190055905246
C	-1.12666255613879	-0.45764961656596	5.98597578759661
C	-1.27237445119653	-1.77838431707661	5.46428224196543
C	-2.56941123713168	-2.25385905746327	5.18813904036132
C	-3.70273063472836	-1.46668816583568	5.43667077578590
C	-3.54946529901987	-0.18787196910875	5.98825632679317
C	-2.27331521262318	0.33568187236364	6.28074656159097
C	-0.05399431048355	-2.66436486892077	5.19509553158794
C	0.33223146176994	-2.63934880899653	3.70187315759018
C	-0.23894556280157	-4.11397605319885	5.68997918434910
C	-2.14859020083451	1.72632584727841	6.90404427032584
C	-2.99126890996504	1.87929320312323	8.18883142773901
C	-2.49956361647681	2.82991671054030	5.88612291013774
H	-0.43230385063226	0.06405237036240	-4.59903886242811
H	-1.16101049963619	-2.49402034066445	-3.22673750963056
H	-2.06412042195542	-1.38401218764068	-4.30531455735271
H	-2.73170826734296	-1.84388060118623	-2.69069473108307

H	0.81842469941931	6.77617239023850	-3.31042685008540
H	3.05594484755455	6.98237377654230	-2.23022617773923
H	4.10617527430810	4.99421063838647	-1.14776083540502
H	-1.01212709088127	3.51765829800485	-3.74680971277572
H	-1.95381814500358	4.47351438358806	-1.67018465637060
H	-2.90935146569572	4.99233613739629	-3.10229086277807
H	-1.76525582410699	6.14198745344478	-2.31899224116767
H	-0.79237906560537	6.43842019909982	-4.75373111930500
H	-1.80645800394880	5.11713040635677	-5.40775705546576
H	-0.01393943671878	5.03647365239999	-5.56033198087895
H	-4.29383512001346	-2.19096380712178	1.44133448221486
H	-2.98182592349366	-4.30172529850448	1.58760358988930
H	-0.75219882533973	-4.46284170474214	0.47771463983811
H	-2.97720130738437	0.97756350425338	0.06366047999958
H	-5.46666893967242	-0.40655367522501	1.28153099424152
H	-5.10137645878792	1.34107769713155	1.20971441263876
H	-4.09208067173610	0.26244093293877	2.23647372283318
H	-3.82684348588204	0.16613786755013	-2.13955307657475
H	-5.09305902150696	1.12122534841705	-1.29882489257760
H	-5.19589090637976	-0.67590189105281	-1.32766395701648
H	0.68190159772814	-1.81362961360799	-1.77207913714824
H	1.49520275770489	-1.25422029733703	0.53295252441118
H	2.67876090830830	-2.27424965040837	-0.37309585733160
H	1.64331399363649	-3.00571305112534	0.90738699019291
H	0.92920036310865	-4.76921985957354	-0.87689347995848
H	1.83521979137975	-3.92823435914229	-2.17005547252359
H	0.07215288508984	-4.23404586176888	-2.36131721597244
H	5.16989318859204	0.09213378108228	7.03478803380342
H	4.49758659654861	0.86964259348047	8.50575127134559
H	5.06851208047141	1.86825823041321	7.11158969579721

H	2.49735900578539	0.02817509480657	8.64019318168757
H	-0.87761441599074	-0.31222787707008	8.70651644485585
H	0.69123165749997	-0.88762684295159	9.38748539663075
H	-0.20006235848538	-1.86162233412167	8.16015696178266
H	6.78033348048556	0.72698450732111	2.95349402511801
H	7.17018336385185	3.18976960282618	2.85121871602247
H	5.55218060673266	4.75256801519391	3.91610229596788
H	3.95617293723231	-0.86212280631909	4.90069190255989
H	6.78943153791976	-1.36191878875634	3.74308794093457
H	5.73811833692279	-2.53281634622683	4.59798163399352
H	6.42335306913803	-1.12470229313001	5.48449694134712
H	3.24586951349860	-0.54265056301239	2.50337937823147
H	3.94737013669205	-2.17910725136009	2.77533332227789
H	4.91627925511138	-0.90052784702498	1.95850782730983
H	2.25450446917481	3.70498207144659	5.25303690885531
H	4.12879620905854	6.12795914023238	4.77633602638397
H	2.35553902095262	6.12883727976087	5.00457559997955
H	3.06097473322678	5.40595132066179	3.51757586640672
H	3.41977960071993	3.46397972037999	7.43533720881887
H	2.81912236511743	5.14264852373542	7.25327175434996
H	4.56523003747974	4.78474467732775	7.00451599734654
H	-2.69707947376557	-3.26550339730089	4.76928318251574
H	-4.70938167296185	-1.85670940359128	5.21069929219038
H	-4.44558714237471	0.41941180251541	6.20140360676376
H	0.80016529451431	-2.23500907720759	5.76219762057620
H	-0.48204621124688	-3.05139832839706	3.06539451192997
H	1.25370660759295	-3.23856464189841	3.52112249804163
H	0.51880599827694	-1.60286729195672	3.33686870050937
H	-0.54438198706239	-4.14774149091789	6.75986154209677
H	0.71468279108869	-4.67958049491016	5.58820384435687

H	-1.00783777378311	-4.66333025450940	5.10156462577309
H	-1.08350026219513	1.86881796244110	7.18854722118983
H	-4.08309085667477	1.82298402289991	7.97806069784606
H	-2.79722543734750	2.86726904449181	8.66460539595568
H	-2.75386334287070	1.08888996168452	8.93545028808010
H	-1.86797026416952	2.76477106565882	4.97179121863342
H	-2.35333358087280	3.83788559958694	6.33640697239621
H	-3.56217543465341	2.75340245100779	5.56096404083417
H	2.35730340804352	1.71515294804694	-0.67028371247540
C	3.15650595008039	2.42477107953387	-0.98745190205746
C	4.02376082688133	1.68311297358300	-2.03034130874869
H	4.50081313871073	0.78538827334839	-1.57525390214189
H	4.83419461478752	2.34509993423931	-2.41292363965645
H	3.42308885645463	1.34246138178779	-2.90122911762555
C	3.97929336355801	2.75185935109934	0.27051919511266
H	3.37472683092374	3.30772619543500	1.01925642893325
H	4.88685014451037	3.35343218360527	0.03693443051119
H	4.32674526979065	1.81392840602351	0.75632969777770
H	1.42818694888076	1.18114723837782	-5.25138737068627
H	2.05830628824676	2.62851840280493	-4.37571526997746
C	1.14367938598535	2.05403831921440	-4.62684176676576
H	0.50727645106904	2.71452820617620	-5.25787638986167

TS2

BP86/def2-SVP

P	1.18270377853961	0.62534139241137	1.84584537443254
C	0.13785684333019	1.83528778148091	1.42629228633120
O	-0.28964042469914	2.91951350130947	3.05162321170116

Si	-1.28185507263646	4.28044050647066	2.79503029988315
C	-3.89317983365159	4.61632626261780	1.43016770028128
C	1.04681758487299	5.42727348793657	1.52846809802256
C	-2.97961334461153	3.61218872269270	2.16137480098874
C	-0.48885078456591	5.40426644991150	1.44826867441637
C	-0.11923832132794	5.74973965021331	5.04089051259090
C	-2.58215535215804	6.18474995950133	4.62199522049336
C	-1.43977869681292	5.15283652632096	4.51427756097651
C	-3.76258444687525	2.85101410547654	3.24711101938450
C	-1.04448970122463	6.84309641196740	1.38732477322257
H	-3.39080677101073	5.09319062638241	0.56029513295619
H	1.47208827174857	4.40080697717504	1.53563444712184
H	-4.80855723326951	4.10719590927838	1.04145708653720
H	-4.24430021817141	5.43246440511243	2.10236377695982
H	1.48370415843993	5.96910376620186	0.65725886176071
H	-2.64099333073563	2.85692380632451	1.40845694966891
H	0.20736619806369	6.62814183874222	4.43921603156172
H	-2.40662910804324	7.06717458881858	3.96704341053232
H	-0.75732725132411	4.89731909471796	0.49170708757188
H	1.39335572884661	5.94414597172057	2.44999755202629
H	0.71369611899571	5.01143374205870	5.02776991192501
H	-3.57020298372329	5.75691726545395	4.34390482148906
H	-0.23163499777932	6.10085431869508	6.09490359224697
H	-2.67299115926300	6.56823504990075	5.66686926502823
H	-4.63949660883527	2.31631643269148	2.81226459255498
H	-4.16662297783327	3.54301596267408	4.02005393105796
H	-1.69786382575585	4.30346862404545	5.19140450077586
H	-0.63641563143517	7.38490518767174	0.50043500624855
H	-3.13789403290066	2.09203359752155	3.76779355572201
H	-0.74726670944499	7.43224560422431	2.28500966789594

H	-2.15379871572276	6.88312101776693	1.31820492861160
Mg	-0.00892806293580	1.32072523660074	-0.65579829699362
Mg	0.71194715756364	1.75854052306325	4.30072018758297
N	0.67297062379489	2.28431242201266	-2.37352747538539
N	-0.79624869313926	-0.32749215696949	-1.67978257925328
N	2.63125168092943	1.87586514431343	5.20230986671520
N	-0.24618378624318	0.87814980451841	6.01256677813882
C	1.22322951528838	2.41136598946032	-4.79617471577913
C	0.59286830757587	1.71711194267125	-3.59515834014857
C	-0.07309700123407	0.49935324617546	-3.87422659565240
C	-0.73283956620966	-0.42368476423689	-3.02438870473471
C	-1.37513403162546	-1.60821466930910	-3.73681031861395
C	1.25504866603138	3.59424430756828	-2.27787190507726
C	0.48507486429195	4.73639383795411	-2.66361825294514
C	1.07521399551474	6.01043458360514	-2.54765972913000
C	2.38783820742852	6.16819400236398	-2.07822342385923
C	3.13092058768103	5.04126788254797	-1.70572541852381
C	2.58683825959459	3.74271927382518	-1.79044965300358
C	-0.94997666944305	4.59738251252490	-3.18839826233524
C	-1.98735675328842	4.71119935177537	-2.05170512636224
C	-1.28635044122741	5.58571321285473	-4.32377392328173
C	3.44800016685337	2.53434607393004	-1.41382617407558
C	4.13545721877713	2.69264673206729	-0.04480590913269
C	4.48921165553267	2.21655537882747	-2.51168588881519
C	-1.38976314118239	-1.42060617707102	-0.96139005460294
C	-2.69764832894871	-1.25985749844920	-0.41003291514295
C	-3.28577029855466	-2.35187657857511	0.25994263010589
C	-2.60939773450979	-3.57185063528979	0.39723249817131
C	-1.31304078324619	-3.70586546745036	-0.11514678126493
C	-0.67584695656399	-2.64770459222959	-0.79462867790722

C	-3.46544149030183	0.05553219512587	-0.57252676749743
C	-4.49184430817662	0.31064093416081	0.54517200201641
C	-4.14759616862715	0.16987763332822	-1.95507826681416
C	0.75880498353230	-2.84287931849778	-1.29945188931124
C	1.77219282564076	-2.76155223902203	-0.13633334363712
C	0.95381529399514	-4.15992042728891	-2.08234318510566
C	4.09181313050627	1.94391856703813	7.23884117491525
C	2.74531794721666	1.74699104299301	6.54234957328843
C	1.70627030657652	1.35784726571140	7.42029365074694
C	0.41882712027973	0.80996289890772	7.17948834619335
C	-0.14757895265367	0.05512186088949	8.37840142516962
C	3.85871490553635	1.90841975453220	4.44881712815098
C	4.55685525950649	0.68532178277187	4.19409650519996
C	5.78101583871407	0.73450787093199	3.49514834381495
C	6.32066833550295	1.94647782988664	3.05331958227477
C	5.61985040758755	3.13739382508850	3.28826589835800
C	4.38741304248989	3.15065660573881	3.97373971100366
C	4.04500110611282	-0.68429591398957	4.65695495282455
C	5.00235597711543	-1.35692468847574	5.66788931134336
C	3.79843188357675	-1.63775127648886	3.46740242868629
C	3.66113470918481	4.48121195910055	4.21200401847593
C	4.09597864989151	5.59729202508131	3.24320750465076
C	3.79306482247252	5.00274191489554	5.66211258113775
C	-1.54124367159698	0.26551344543243	5.87736185145528
C	-1.64960957941094	-0.90320880035667	5.05582282393409
C	-2.92303719698807	-1.44885800622351	4.80220153983316
C	-4.07947039656951	-0.89060902852959	5.36355861920246
C	-3.96195143325288	0.21448444607412	6.21285051959449
C	-2.71399985369279	0.81035124296144	6.49628747271541
C	-0.40005345767736	-1.62074778336290	4.54243712994310

C	-0.61030880148493	-2.40198346300527	3.23640797300545
C	0.17999022129947	-2.54903534924501	5.63498613582553
C	-2.69792450538225	1.97597466291057	7.49512051578980
C	-3.19584721705939	1.53785724425357	8.89523912906032
C	-3.55044936383620	3.17589149981564	7.03182412900984
H	2.20776749436822	2.85454709422247	-4.53904845920207
H	0.58781176187304	3.24978411076356	-5.16043131278411
H	1.35680686567384	1.70029245313506	-5.63707045661522
H	-0.08084896637407	0.21974951943444	-4.93815139004636
H	-0.69531397517404	-2.48910932972099	-3.73965503935645
H	-1.59625499804503	-1.35462064809044	-4.79421685006548
H	-2.31290062739408	-1.93199896325714	-3.24002105078552
H	0.49961528229915	6.90395092155219	-2.83955363875131
H	2.83141968396969	7.17525501564873	-2.00360343224443
H	4.16366722536044	5.17184542315086	-1.34172708508296
H	-1.05967898099759	3.57424177367149	-3.60539401767189
H	-1.81318606432822	3.94439605916523	-1.26441753096491
H	-3.02015677030578	4.56708027745501	-2.44316757386644
H	-1.94008368102170	5.70971738809392	-1.56262875629557
H	-1.35132120564524	6.63627184352777	-3.96123261877115
H	-2.27511927426014	5.33529696104941	-4.76951153571327
H	-0.52652807092284	5.55628157475577	-5.13649939099290
H	2.77196577621753	1.65169692578526	-1.34542626998172
H	4.89605346124316	3.50560801258054	-0.05221450309559
H	4.66218830441797	1.75494482100073	0.23884926656040
H	3.40039537950822	2.91721583909574	0.75746598194351
H	4.01016552990539	2.01491478398062	-3.49459746852940
H	5.08763578505134	1.31888309212226	-2.23518508409161
H	5.19594653876981	3.06681498383388	-2.64809738133174
H	-4.29774794968053	-2.24994568187333	0.68197893805480

H	-3.09094669126935	-4.41687284015434	0.91746175962306
H	-0.77859866121380	-4.66141183223363	0.01740700605905
H	-2.71320258974174	0.88024689958479	-0.51375537889921
H	-5.36525872279757	-0.37613211149606	0.47045017758112
H	-4.88815040372322	1.34683814184916	0.46676001195695
H	-4.04202088717313	0.19098421853824	1.55426859116258
H	-3.41403647843994	0.13775345106183	-2.78786996670256
H	-4.70579393908283	1.13036802296302	-2.03502946451673
H	-4.87495278349554	-0.66060895976753	-2.10225391321338
H	0.99464043412942	-2.00587697414158	-1.99042006177024
H	1.68898688322137	-1.79379573398306	0.40552410145057
H	2.81509932432673	-2.85700117275602	-0.51635791587253
H	1.60179198965509	-3.57899619934860	0.60073557208837
H	0.86720586260353	-5.05180941910822	-1.42162347262801
H	1.96890342448104	-4.18961887723551	-2.53855290134653
H	0.20819949981010	-4.27847489537677	-2.89982293369427
H	4.52734742999775	0.96268897859793	7.53074074720650
H	3.94749900041065	2.52241691909902	8.17705882565502
H	4.83624469328551	2.46548942895458	6.60721440156094
H	2.02091534331976	1.29405848911342	8.47468827371887
H	-0.37446714301765	0.75675644668896	9.21118312964099
H	0.61112528078951	-0.66127981258706	8.76190108713285
H	-1.06906216035126	-0.50767605778913	8.13423212097763
H	6.32842600191054	-0.20337734857544	3.29931839495183
H	7.28493531660016	1.96668582639773	2.51794870922168
H	6.04966099356166	4.08371849667077	2.92687881661218
H	3.07056198246105	-0.52824533487039	5.16690799328111
H	5.98041599445723	-1.60472428691219	5.19609408005104
H	4.56417482039694	-2.30863325837585	6.04538651518752
H	5.21220355696919	-0.70728991987221	6.54437442176684

H	3.09224233287806	-1.19477229388713	2.73300455943057
H	3.36685894686564	-2.60061034169409	3.82453027640535
H	4.74492808404318	-1.87616920331725	2.93097204155325
H	2.57764395752670	4.28813872954414	4.02981973565782
H	5.13737114737816	5.93611188604488	3.44644966646412
H	3.43958111349617	6.48540381036300	3.37029390425125
H	4.03333510787581	5.27940777822504	2.18022847243483
H	3.32065035326956	4.32448032091626	6.40070937520411
H	3.29601449846103	5.99460308441823	5.75704567843589
H	4.86371806459411	5.13193477284078	5.94101883898199
H	-3.01200158192828	-2.33829984895559	4.15891237717313
H	-5.06958900342900	-1.32814275537345	5.15190973208214
H	-4.87130029852329	0.63221370877774	6.67708465992953
H	0.37642411653020	-0.84717982008351	4.33421253538390
H	-1.24578285859921	-3.30360286245424	3.38664066743312
H	0.36782757023439	-2.75682452249522	2.84390477972145
H	-1.08380959462694	-1.77800094470986	2.44790543730780
H	0.43207610653711	-1.99285033113614	6.56275325982574
H	1.10779389312259	-3.04784441255030	5.27297375350574
H	-0.55440719793356	-3.34268513512148	5.90274267302662
H	-1.64983338617716	2.33200555547864	7.59239680798578
H	-4.28328890231698	1.29948237856976	8.87177196792290
H	-3.05434637551184	2.36136618720817	9.63172838554434
H	-2.67006897694466	0.63963480391107	9.27952411826055
H	-3.24139244257218	3.53778853922668	6.03183571914036
H	-3.45288711398690	4.02087595943000	7.75052930172635
H	-4.63001424701140	2.91003601179111	6.97376177512175

($(^{i}Pr)_3SiOCP$)

BP86/def2-SVP

P	0.27965530690757	-1.47556159246208	2.62805750876218
C	0.19724383897323	-0.55775156799328	1.34335867786653
O	0.12832670113080	0.21625828853154	0.31999552427750
Si	0.05008173960467	-0.29532007237003	-1.38146516174099
C	-2.00100176927105	-1.54937515261397	-2.98311348874978
C	2.92859404817471	-0.48335318040797	-1.43084271577994
C	-1.53938427068798	-1.35160715515393	-1.52288569395686
C	1.64551062043063	-1.32211883331841	-1.60023734333529
C	0.67454620760102	2.53137710310966	-1.59252197473543
C	0.31334252088419	1.26902429637880	-3.77897858533590
C	-0.08021795931090	1.38043537547887	-2.28926428166291
C	-2.68634138040054	-0.79657475882817	-0.65190364584657
C	1.69002060330155	-2.18633004491126	-2.87682794929015
H	-1.21191431471732	-1.97463625111336	-3.63645548234813
H	2.92159109950156	0.11027414956926	-0.49445181511017
H	-2.86986257187547	-2.24127080175201	-3.02650759502548
H	-2.33237566812311	-0.59185737913548	-3.43836980712234
H	3.82464703027379	-1.14025975977460	-1.39869006454082
H	-1.25251410727803	-2.34777785528109	-1.11218742942352
H	1.77266469287874	2.37171519439537	-1.60916118731270
H	1.39546992751387	1.05174600954115	-3.89953792843741
H	1.58264635134080	-2.01670101430155	-0.72736524267738
H	3.07403855395501	0.22092925284546	-2.27713185265194
H	0.37199127216476	2.64699520958820	-0.53355434979095
H	-0.24722267881511	0.47735147003678	-4.31693052100935
H	0.47641696251877	3.49418808221048	-2.11164256029647
H	0.11594419129127	2.22634488213369	-4.30808736039862

H	-3.59033336036458	-1.43585224682121	-0.75066301683444
H	-2.98118243401667	0.22904507518973	-0.96187038607289
H	-1.16924744276704	1.61953178955928	-2.24526982069128
H	2.60748073079381	-2.81408130600334	-2.88887641110954
H	-2.41982709557078	-0.76257414604411	0.42306110551561
H	1.71028108468940	-1.56887000935247	-3.79954042020641
H	0.82513592156845	-2.87645355322993	-2.95268645392812

$(^{i}Pr)_3SiPCO$

BP86/def2-SVP

O	0.36755937048636	-2.07035527773881	2.06958146800012
C	0.28594094245854	-1.01408694175653	1.55380656661995
P	0.19284524329577	0.53902795177561	0.93051277310448
Si	0.05214957341613	-0.24942394031115	-1.26268210189169
C	-1.93318129040547	-1.59473889591934	-2.92654163431289
C	2.93438969676557	-0.46854273396588	-1.42747484498496
C	-1.55524808909588	-1.30733287422355	-1.45701966424943
C	1.64548190632032	-1.30257344753863	-1.56490193791804
C	0.65717299475643	2.60292084709737	-1.67991863406740
C	0.31676835076810	1.19171211582348	-3.76082322330621
C	-0.08260494341850	1.39284254624316	-2.28170309517362
C	-2.76376146514045	-0.70679242520392	-0.71009556293470
C	1.65747887074144	-2.14648660498922	-2.85552978011660
H	-1.10834068738251	-2.04772769770275	-3.51192825858291
H	2.95821233995992	0.10497537743459	-0.47820930927603
H	-2.79510799615679	-2.29611236073713	-2.97536554839502
H	-2.24883920220246	-0.66763582925921	-3.45083602534831
H	3.83185015194345	-1.12491357692402	-1.44408889078467

H	-1.29806954984065	-2.27776343335350	-0.97012182627920
H	1.75558576057132	2.44358218490578	-1.64778767951451
H	1.40563714946985	1.00365025663488	-3.86408589893343
H	1.61877477937717	-2.01750436945035	-0.70876379735422
H	3.04751487074021	0.25501478663101	-2.26259879054974
H	0.32539792294226	2.83218933718640	-0.64650214957859
H	-0.21544365115327	0.34803034910683	-4.24365439767762
H	0.47458688929979	3.51401036107014	-2.29131348129148
H	0.08974167816126	2.10565867359847	-4.35308754750715
H	-3.65013689203324	-1.37258713271063	-0.79797521779789
H	-3.05559974255274	0.27675976738719	-1.13758346802989
H	-1.17454540844999	1.61975005601558	-2.25327324953816
H	2.58080933509903	-2.76508375148373	-2.90301402810392
H	-2.55859616390410	-0.54802632447030	0.36704482755027
H	1.64319138784115	-1.51635572928854	-3.76867055551333
H	0.79724586732198	-2.84303126388329	-2.91527503626316

[(^{Dipp}NacNac)Mg]$_2$

BP86/def2-SVP

Mg	-0.01357419371274	-0.05974504011483	-0.00879644091471
Mg	0.01668186840819	0.01788903011354	2.90785388442721
N	-0.22986994357271	1.35391263364307	-1.58633762228288
N	-0.02488067443889	-1.68478384141030	-1.36539215743086
N	1.50744075763825	0.65147955080390	4.27390157925463
N	-1.39556514711890	-0.27881369146365	4.46781196425134
C	-1.13298503487428	2.04689887967019	-3.79559003954467
C	-0.75197413211676	0.98343778869245	-2.77563041498999
C	-0.93031325218479	-0.35655537475786	-3.20201209138530

C	-0.48974848979911	-1.57504600391023	-2.62189857951214
C	-0.52612342809969	-2.79845569161383	-3.52289538201128
C	0.19225683682078	2.71918261171143	-1.44569987962248
C	-0.58573955534997	3.65562062954518	-0.70025550408850
C	-0.14100060851450	4.99129872609099	-0.60883096511088
C	1.04651889234826	5.41225819089614	-1.21796131917473
C	1.82338929206060	4.48078305658239	-1.91886874142710
C	1.42442624199590	3.13594894775353	-2.04500962240753
C	-1.90334795572457	3.27020505417959	-0.02791682544664
C	-1.93057751296157	3.67419582409888	1.45858207081590
C	-3.11692148443180	3.85794656677092	-0.78043799652510
C	2.34738412784221	2.15638699231057	-2.77628188419310
C	3.59780492604354	1.85390307686030	-1.92395162510616
C	2.75528341905133	2.64318663180511	-4.18215192426351
C	0.46003355683506	-2.95134936061511	-0.89632314467343
C	-0.44702058827637	-3.93185870371641	-0.38727452133250
C	0.07709053679620	-5.12998516483014	0.13910474535354
C	1.45531882085246	-5.37892165831756	0.16517096655717
C	2.33614421146778	-4.41574390831356	-0.34227629092906
C	1.86669999005871	-3.19786962068234	-0.87614805726033
C	-1.96257110960711	-3.71978552812943	-0.37757605702104
C	-2.48603049748928	-3.50091855330555	1.05482939018593
C	-2.72641275919531	-4.87596206414093	-1.05832285844605
C	2.87737314245516	-2.18713188378488	-1.42229672166710
C	3.82432370053450	-1.68511943063453	-0.31470104975510
C	3.67292158366587	-2.75571034602920	-2.61557464571326
C	2.28494583332097	1.71148565238589	6.38260734139484
C	1.20237049564553	1.10888735265972	5.50032468563282
C	-0.10148648508794	1.06476377090086	6.06292143794734
C	-1.26299370374594	0.36795656812705	5.64617580833277

C	-2.40153879096089	0.32850455324768	6.65456090417731
C	2.87300871775473	0.62462804508502	3.83933881978937
C	3.57223620533531	-0.62365740495819	3.85032505424291
C	4.90483823411977	-0.65806072164469	3.39301723917787
C	5.54418167457434	0.49602468099374	2.92042713220747
C	4.84369560390475	1.70770048563968	2.89109786751074
C	3.51054777011415	1.80085693930202	3.34063646160385
C	2.91366050160695	-1.89404563777606	4.39419165230114
C	3.12710110504143	-2.02929608055625	5.91775627699126
C	3.35434833452251	-3.17759204794047	3.66945717639247
C	2.78419432889910	3.14314467582085	3.23525912154549
C	2.43693216928858	3.46218813828325	1.76815825166594
C	3.57514749252606	4.30615216580631	3.87006894605013
C	-2.50582448596223	-1.17995388255944	4.32641498114126
C	-2.40503403394694	-2.50109633518187	4.86864117147236
C	-3.50401686640838	-3.37439864747276	4.74777032326104
C	-4.68536135638310	-2.97907084749240	4.10680559160415
C	-4.76521092123521	-1.69682602330916	3.55129213979036
C	-3.69278229697913	-0.78494446417763	3.63863759974384
C	-1.12779053848145	-3.00784615382950	5.54434641446554
C	-0.43660226920542	-4.07777847430224	4.67190395830095
C	-1.37591772594178	-3.54268029673634	6.96998017067444
C	-3.84591396491154	0.59384081003071	2.99583531965780
C	-4.91690537707663	1.45189711187300	3.70202086722612
C	-4.14815103571042	0.47964357449913	1.48898076294099
H	-1.63439203241785	2.91080639148699	-3.31835573388639
H	-1.79656922059204	1.62814915981991	-4.57587944048372
H	-0.23228672017902	2.45253091726050	-4.30041821117418
H	-1.36931702001462	-0.45952160205223	-4.20296364508192
H	0.48948296033452	-3.23393028638364	-3.61986766754001

H	-0.89993639945859	-2.54698498712128	-4.53195664698762
H	-1.16188938539385	-3.60202174192379	-3.10053102463746
H	-0.74682757918976	5.72143785712985	-0.04928951299545
H	1.37111124184134	6.46156961789598	-1.13911264589962
H	2.76742096508992	4.80636283268232	-2.38327066370919
H	-1.98459045969914	2.16193476469529	-0.07582800367506
H	-1.07408964373571	3.23328350937225	2.00909729847873
H	-2.86423371076669	3.32290260731669	1.94320564093025
H	-1.88845601280038	4.77568715252003	1.59063936184863
H	-3.08970103538870	4.96842090526346	-0.78003013387056
H	-4.07027443500955	3.54446729046051	-0.30534709976538
H	-3.14506923613194	3.52458183785552	-1.83712494916907
H	1.79447608334652	1.20357438906915	-2.90302969162789
H	4.20480586951237	2.77047013104797	-1.76456897753849
H	4.24600306845308	1.10510089124535	-2.42638730035323
H	3.32651848285857	1.45871771995998	-0.92484202871338
H	1.87199141266198	2.86425146194462	-4.81582181623583
H	3.35863140899927	1.86950835775872	-4.70156753588020
H	3.37202294420981	3.56545441965234	-4.13732294732705
H	-0.61517799744576	-5.88740329423377	0.53857441839826
H	1.84221723969902	-6.32354072714436	0.57800804158458
H	3.42014113922140	-4.61445151516724	-0.32932412383948
H	-2.17553198940051	-2.79474607608542	-0.95183941508085
H	-2.28333419016944	-4.38260446628302	1.69743110158857
H	-3.58186289803674	-3.32776916722277	1.05882694358301
H	-2.00467224409932	-2.62423104684554	1.53515226094293
H	-2.35484333082064	-5.07676988000098	-2.08439813424976
H	-3.80823757479037	-4.63813092585464	-1.12872535089350
H	-2.63516953766812	-5.82214679507002	-0.48520165009619
H	2.30285533500136	-1.31316242507199	-1.79449731873000

H	3.26282090506275	-1.23345365481439	0.52895092663764
H	4.52721851531392	-0.91886932990786	-0.70206152742322
H	4.43491540275589	-2.51425261011425	0.10006314143923
H	4.30674195622253	-3.61613623132535	-2.31145731530748
H	4.34786385795424	-1.98394799138513	-3.04155162320076
H	3.00283391002904	-3.10585945209882	-3.42694969094411
H	3.22022144848791	1.12012426362210	6.33748348764700
H	1.95240677813119	1.78100003862466	7.43481661536768
H	2.54523963217056	2.73423504306580	6.04029888603625
H	-0.18834835480062	1.54494992559313	7.04654044587520
H	-3.37309537528379	0.55989006299019	6.17558768661423
H	-2.22867458908291	1.04215536769421	7.48100206067957
H	-2.51111432226847	-0.68438854891297	7.09284583221160
H	5.45522893754411	-1.61046947712010	3.40918845787300
H	6.58636421803918	0.44847445878779	2.56913359895549
H	5.34365667808797	2.61082828077769	2.50707010922539
H	1.81733272526485	-1.78233435443806	4.23531973414343
H	4.20852498851515	-2.10502649721546	6.15866512364373
H	2.62754981492250	-2.94130546619485	6.30586418934422
H	2.71650636390401	-1.16249720983546	6.47078520368808
H	3.22244151951220	-3.09696243220280	2.57194385360620
H	2.75132144856898	-4.03949373131222	4.02014988192940
H	4.41704587622636	-3.42799252976636	3.87167112548643
H	1.82445471006599	3.05079124881675	3.78390558190992
H	3.35316522347193	3.56945264172859	1.15162213888726
H	1.86453210502300	4.40892442579572	1.68767496956032
H	1.82198914488900	2.65881803633921	1.31209708870760
H	3.87206434843710	4.08844068521922	4.91659186447628
H	2.96579592027299	5.23395518246707	3.87369446977596
H	4.50415332028634	4.53144055459714	3.30518958228833

H	-3.43226284253707	-4.38930460702352	5.16893624485499
H	-5.53750719124353	-3.67259871786563	4.03077142182828
H	-5.68904192495111	-1.39131095600594	3.03513483707553
H	-0.43319282472939	-2.14781947171277	5.63077630231441
H	-1.08252689924586	-4.97223853986866	4.54628459972124
H	0.51280760617239	-4.41336989271676	5.13792385981523
H	-0.20431126651677	-3.69325642892176	3.65874842413382
H	-1.86721607863528	-2.78481302133163	7.61385873378026
H	-0.41697346616685	-3.82398581006343	7.45211069181614
H	-2.02037670186300	-4.44712946184233	6.96774010243418
H	-2.87140227383547	1.11499488453662	3.10388815728974
H	-5.92446294526812	0.99203429871903	3.61761217513622
H	-4.97404527734672	2.46368705759319	3.24893797930932
H	-4.69945943841758	1.57977355367122	4.78175444346247
H	-3.36119334683320	-0.10108194190853	0.96520229691215
H	-4.20381669049943	1.48300733696185	1.01947176049638
H	-5.11953519585097	-0.02422209214862	1.30078295671916

[(^{Dipp}NacNac)Mg(dioxane)]$_2$

BP86/def2-SVP

Mg	-0.01017058561806	0.06783659436046	3.18846657353100
O	-0.03614997022537	-2.11660164181222	3.93921193580516
N	-1.51552930012842	0.74989940838194	4.68724078306540
C	-2.30956556539103	0.93847137361067	7.03557870639319
H	-2.67728694748218	1.98422914406115	7.02655926817765
H	-1.94185217924650	0.71678131420025	8.05486119693697
H	-3.19058370930761	0.30246493343255	6.82328156248328
O	0.24057328429990	-4.86329182286922	4.68408220497568

N	1.56734758936585	0.73634469400201	4.57589916204553
C	-1.21267865596619	0.74272924876118	5.99821849396488
C	0.09556974549568	0.59011150432089	6.53133116735128
H	0.13440254492670	0.53606656550931	7.62813677307140
C	1.36693450838135	0.69165488816635	5.90620296745099
C	2.55280527875907	0.75746947809236	6.86224960898874
H	3.20967925537358	-0.12742693946332	6.73472704529387
H	2.21956409002387	0.79602618597679	7.91583840616235
H	3.19134995782456	1.63976733414533	6.66150881988427
C	-2.84334130453599	1.18165228609840	4.34215133098743
C	-3.88151502679664	0.23474815184555	4.08591946541743
C	-5.16979162486944	0.70151414981625	3.75240513488765
H	-5.97042244184865	-0.03005280755082	3.56011280696314
C	-5.45703455551736	2.06897901373383	3.67610941821985
H	-6.47065331999005	2.41342318405137	3.41880067613516
C	-4.43817689032432	2.99394660681712	3.93410552656268
H	-4.66266165287947	4.07173338229250	3.87863137944736
C	-3.13380762071013	2.58163120552779	4.27110903724919
C	-3.66225345209641	-1.27248057762728	4.21703091989739
H	-2.58939345911837	-1.42092562152874	4.45478479773450
C	-3.95699110305768	-2.01368543336981	2.90128002751557
H	-5.01440720326910	-1.89342697078101	2.59064216962910
H	-3.77648466152846	-3.10404972742177	3.01289807286151
H	-3.32946084988218	-1.64066512398594	2.06768930166499
C	-4.49143435382003	-1.87719178570462	5.37149623967809
H	-5.58074276528376	-1.79392583677693	5.17201767977605
H	-4.29436959814290	-1.37100177177179	6.33882703879373
H	-4.25862310734405	-2.95549405146111	5.50114310429462
C	-2.08086728499730	3.65595786458708	4.55729805239374
H	-1.14810428291925	3.13878532521216	4.86079918228817

C	-2.48986774592603	4.59649195576465	5.71111471230958
H	-1.66970988505076	5.30701848818968	5.94393197889884
H	-2.72239986477895	4.03860244795326	6.64138679035738
H	-3.38627547905748	5.19971184514282	5.45433215029281
C	-1.76243372779039	4.47164906938810	3.28941697290447
H	-1.42011605141559	3.81029578815336	2.46967178831557
H	-0.96798655817806	5.22075447476563	3.48956790475676
H	-2.65537192875370	5.02025815166052	2.92200513128547
C	2.89072816324665	1.10092913139114	4.15368335640071
C	3.90413450687519	0.11507158433732	3.96618763456794
C	5.21037789407703	0.53444000748011	3.63901470157299
H	5.99760628689935	-0.22309767013837	3.49775673707265
C	5.52951319721092	1.89022580960170	3.50337263283256
H	6.56014110676715	2.19886418371618	3.26818087455287
C	4.51985176415661	2.85148712863352	3.64690468182335
H	4.76984808878510	3.91535042512990	3.51506620599954
C	3.19392415578358	2.48555970379046	3.95310178133170
C	3.61403750062023	-1.37898996865756	4.10191332887003
H	2.58867280198616	-1.47412263019314	4.51507086204780
C	4.58492873116321	-2.09897163179820	5.05969842749205
H	4.27970325172756	-3.15703791089923	5.20268182835583
H	4.61984678295664	-1.61920149251598	6.05934542242316
H	5.62084604518671	-2.11133357271968	4.66156288055748
C	3.61774557003053	-2.05579682859260	2.71787042179471
H	2.86396761992019	-1.60301729085243	2.04069737253503
H	3.39573873162703	-3.14019445604807	2.80592316061097
H	4.60449491013086	-1.95801096080081	2.22171513627310
C	2.11439996611882	3.56419568731192	4.08366885901662
H	1.14764784967583	3.06407443688274	3.85762807944574
C	2.00589624818497	4.13070474930767	5.51617057402214

H	1.73333030463057	3.35209458786990	6.25331940606574
H	1.22563465379157	4.92010303513977	5.56406338976292
H	2.96704853412289	4.58656641541532	5.83580536882748
C	2.29903557857448	4.71773701078938	3.08014575266449
H	1.41301178987495	5.38610728470810	3.09248781279857
H	2.43768264689666	4.34755147749689	2.04504184249585
H	3.17693449832057	5.35087483269462	3.32831565301315
C	-0.35075875316945	-3.18598580263745	3.01866546687485
H	-0.13322909761414	-2.78769314893134	2.00536813118097
H	-1.43908882317307	-3.40669547930614	3.08902327121416
C	0.46014892766869	-4.43419534589653	3.34414576194416
H	0.15456963592044	-5.26547296734349	2.67727207643179
H	1.54620156605093	-4.23071767650760	3.17510032197886
C	-0.23079759567400	-2.55820927182825	5.30552066762716
H	-1.31519405957227	-2.76906784230015	5.45694407830693
H	0.06386209658005	-1.71359803229660	5.95865173894096
C	0.57693962570584	-3.81910234514523	5.58836436405193
H	1.67141565885880	-3.59253112803172	5.53405083665685
H	0.35174256446306	-4.18477425948335	6.61159433832928
Mg	0.01056415371598	-0.06780906989063	-0.03869453533846
O	0.03679448613035	2.11651190596290	-0.78928686456625
N	1.51550012713868	-0.75027552663932	-1.53760190301785
C	2.30910705242472	-0.93901742869269	-3.88606275709681
H	2.67618902238368	-1.98500518835384	-3.87743308710365
H	1.94139177799760	-0.71679422734566	-4.90523108976525
H	3.19052153459833	-0.30359473016161	-3.67367468542979
O	-0.23966807570010	4.86325837210177	-1.53414349256988
N	-1.56737092680431	-0.73607308428856	-1.42579425830662
C	1.21243477900557	-0.74301662799165	-2.84852563665718
C	-0.09584837236312	-0.58988521459342	-3.38142321919420

H	-0.13482865400215	-0.53562602933673	-4.47821361140713
C	-1.36715105355606	-0.69109993977049	-2.75610981919247
C	-2.55325395324377	-0.75591055755113	-3.71194377993099
H	-3.20963902160399	0.12927769599827	-3.58388926542367
H	-2.22026827226278	-0.79419820138869	-4.76562356984726
H	-3.19221271824359	-1.63795420684130	-3.51142253500759
C	2.84328980647667	-1.18222794852514	-1.19271092490214
C	3.88149607429255	-0.23543827660294	-0.93617422221826
C	5.16967748001846	-0.70233443749683	-0.60247915096790
H	5.97034805690075	0.02915224801632	-0.41005129844357
C	5.45676600430401	-2.06982767627606	-0.52614488030728
H	6.47031186574576	-2.41438153570392	-0.26869414641903
C	4.43787667888993	-2.99468566326265	-0.78440071284492
H	4.66227094937378	-4.07249452340294	-0.72900766838758
C	3.13365232497213	-2.58223588235708	-1.12180863652965
C	3.66239379033271	1.27180993398616	-1.06722636920367
H	2.58950471645093	1.42038873342904	-1.30475184498952
C	3.95749046083499	2.01297875937238	0.24845173155554
H	5.01496875299044	1.89267824329162	0.55886586424604
H	3.77700907309518	3.10335356608678	0.13688978091661
H	3.33012119227517	1.63998490403027	1.08217670641723
C	4.49139855304263	1.87637040965702	-2.22189529587349
H	5.58073986728379	1.79282541817124	-2.02270722257548
H	4.29393801523963	1.37025324134149	-3.18918396440801
H	4.25881345434096	2.95473370007999	-2.35145071084634
C	2.08059158109505	-3.65647629049416	-1.40784678500323
H	1.14778181097798	-3.13926368935350	-1.71115678266683
C	2.48931655669886	-4.59700744032648	-2.56176364118033
H	1.66888449164181	-5.30717090624528	-2.79470942064893
H	2.72216602691116	-4.03910782522312	-3.49195476279507

H	3.38544946743336	-5.20064621783997	-2.30500210836158
C	1.76240860188265	-4.47225274236983	-0.13996091544112
H	1.42020578764386	-3.81098242619500	0.67989420860903
H	0.96795892587848	-5.22137097370861	-0.34003845637677
H	2.65545623247684	-5.02083217568978	0.22722658107648
C	-2.89076837288626	-1.10045621426660	-1.00347008262228
C	-3.90372431300358	-0.11432326233301	-0.81500279291485
C	-5.21008966076135	-0.53338056271342	-0.48793531390536
H	-5.99698302465511	0.22438266922553	-0.34602430551609
C	-5.52976157381229	-1.88913645493030	-0.35325634492978
H	-6.56048921165030	-2.19752679127468	-0.11817646958127
C	-4.52052497910285	-2.85070725255350	-0.49768207601848
H	-4.77095709866658	-3.91456955038456	-0.36664288953603
C	-3.19450042814718	-2.48511034570039	-0.80387903721567
C	-3.61298896525860	1.37969493961161	-0.94966968490045
H	-2.58774741101017	1.47466667453300	-1.36316960284037
C	-4.58391593580250	2.10088235057299	-1.90650671099648
H	-4.27832134773538	3.15895327041713	-2.04867541555453
H	-4.61934329937513	1.62198135638322	-2.90655387674465
H	-5.61969987697566	2.11330945839700	-1.50802307972479
C	-3.61579020107294	2.05535425462738	0.43493868249116
H	-2.86197830960838	1.60158996516953	1.11140752675432
H	-3.39322752241673	3.13969993937607	0.34768869851737
H	-4.60239210542830	1.95768875377493	0.93139457277303
C	-2.11552001601300	-3.56414471744100	-0.93573024069164
H	-1.14828981249804	-3.06430649727462	-0.71114903501979
C	-2.00917325690144	-4.13096411963999	-2.36827647320199
H	-1.73743419427287	-3.35258415931166	-3.10597653971327
H	-1.22920407495682	-4.92059340086352	-2.41712104898197
H	-2.97090244655642	-4.58659056721461	-2.68651115244598

C	-2.29922403490218	-4.71740514135221	0.06828550658249
H	-1.41353836336679	-5.38620264946580	0.05481575784419
H	-2.43621613276483	-4.34693189539786	1.10350626836177
H	-3.17777793267553	-5.35016236169306	-0.17851256694064
C	0.35110820917423	3.18587215906330	0.13139363679306
H	0.13323151848017	2.78756284586538	1.14460975983289
H	1.43946171954018	3.40656427355607	0.06140112488877
C	-0.45966947078498	4.43410600435958	-0.19430252407860
H	-0.15427830114238	5.26533885717497	0.47271399138693
H	-1.54578127990832	4.23065363686832	-0.02558323390443
C	0.23190993876245	2.55819604915530	-2.15551776267550
H	1.31636131507531	2.76905244153144	-2.30655065668640
H	-0.06253943054728	1.71362066833627	-2.80878927477098
C	-0.57572584394599	3.81910204589906	-2.43857979599999
H	-1.67022006329582	3.59252489625332	-2.38465954768539
H	-0.35017536083556	4.18481224138373	-3.46171753786894

Dioxane

BP86/def2-SVP

O	-0.00160142771981	0.02369242408395	-0.00680152255631
C	-1.17278602065193	0.36515138106068	0.72950252504478
H	-1.23224575431262	1.47382605756388	0.87349835239243
H	-2.04392921750043	0.04333968702586	0.12149569657660
C	1.16997517321087	0.36451410898585	0.72915976197030
H	1.23015036646408	1.47316567348647	0.87302979605792
H	2.04079266122230	0.04214344741925	0.12098042270929
C	-1.17283104485380	-0.31954689805547	2.09699287296696
H	-1.23289372166903	-1.42820508999665	1.95338381119767
H	-2.04371374750542	0.00310197925070	2.70492309848530

C	1.16997947366200	-0.32020958273288	2.09665000726840
H	1.22929558106660	-1.42888488089874	1.95284134883863
H	2.04122875983845	0.00181826619537	2.70438394407095
O	-0.00121108125126	0.02167342661175	2.83285988497707

[Ge(^{Dipp}NacNac)(CP)]

BP86/def2-SVP

Ge	0.02066524082133	-0.64931385915879	0.07930947954683
C	-0.10808849559721	-2.61544151619078	-0.37233814513258
P	-0.18346645484261	-4.19495384656398	-0.37790455951042
N	-1.48022590625439	-0.14743056005263	-1.24308593205059
C	-1.31802320824072	-0.24716274631184	-2.57259168616953
C	-2.49456294189033	0.03524237436679	-3.48422916935052
H	-3.34224667523857	-0.64573661768329	-3.27331164944080
H	-2.87641858564348	1.06294804497705	-3.31631813535334
H	-2.20857327391418	-0.06651271577080	-4.54662715937001
N	1.46642508163158	-0.28265866931471	-1.33609782420793
C	1.21931393928134	-0.48605669617934	-2.64606767830249
C	2.38749183497740	-0.60034686444632	-3.60366510519771
H	3.06256429914199	-1.42168027883153	-3.28800832769999
H	3.00330594649273	0.32083083029986	-3.60588373665691
H	2.04148074297140	-0.79680041796047	-4.63472210195422
C	-0.08135442263253	-0.56190488357811	-3.19232526666533
H	-0.12753397710884	-0.72311182629773	-4.27712757580553
H	-0.70251512875627	4.03281021850183	0.89395251208714
C	-1.51927600938294	3.28789051384014	0.97541266445888
H	-1.20212708512478	2.50679554128485	1.69545775622565
H	-2.40009936822796	3.80805148085118	1.40634043984995

H	-4.35104673493868	3.05216569392245	0.52298631370801
C	-4.19206367341171	2.00307924619598	0.22778715304541
H	-6.20796308333044	1.40459415094742	0.77911430388889
H	-1.37093194398060	4.49980537213180	-1.53506925526984
C	-2.19932341887936	3.76814891224297	-1.43221781353415
H	-0.92314282929409	2.16539599816001	-0.76497485443204
H	-2.39761129045146	3.33907744627671	-2.43564851228626
H	-3.10538669735090	4.33219260949310	-1.12608319225844
C	-1.84020994611953	2.67502723055478	-0.40363649531846
C	-2.93866196357290	1.61881295541629	-0.29070686416776
C	-2.74786037762302	0.26077441513042	-0.68260133205162
C	-3.79177346450098	-0.69780565704640	-0.49965056938217
C	-5.23515789223818	1.08248455713540	0.37620202140149
C	-5.02423205687439	-0.25495752290144	0.02287559649644
H	-5.83701447191057	-0.98491605395343	0.16215958226326
H	-4.46991928806917	-3.78731960079535	-2.03418118168591
H	-4.68516617212477	-2.15789432696994	-2.74388873387927
C	-4.67679224558294	-2.72483219991311	-1.79014114530386
H	-2.62154688965074	-2.33456798757808	-1.25284675383084
C	-3.62254908935134	-2.18762053112910	-0.79970570962618
H	-3.44324826397605	-4.07716886414741	0.30205744032384
H	-5.70298199470321	-2.67720242606000	-1.36892232695092
C	-3.64863661287242	-3.00617249948287	0.50919599220969
H	-2.88004276178740	-2.65225618060896	1.22420659949076
H	-4.63848133179853	-2.93924277858634	1.00866607937256
H	4.05798535271062	-4.28643815832765	-1.11262040773462
H	4.50570807466944	-2.90753938487892	-2.16624303894220
C	4.39106588174819	-3.22793928066328	-1.11001167595847
H	5.39848562988652	-3.20084939795077	-0.64375184577936
H	2.38741996592799	-2.49647042344468	-0.82839981921297

C	3.37607763091505	-2.35023731275539	-0.34940870679963
H	2.92282580691562	-3.87403720369507	1.16395583230087
C	3.24056123071974	-2.81190622325711	1.11701722508638
C	3.71258971796112	-0.86286935220193	-0.42461764431004
C	2.78445773249079	0.11216836416728	-0.89687829721543
H	4.20389092083971	-2.71700225286075	1.66189858224344
H	2.48379655170517	-2.21217443320227	1.66254470384682
C	4.98740769092904	-0.43191668689176	-0.00287725416751
H	5.70926233471675	-1.17870811329406	0.36341996156717
C	5.35554403005272	0.91728939951250	-0.04410196959353
H	2.72695935405446	2.47809755725234	-3.53604951628298
C	2.63094031117387	3.22806494860555	-2.72553602565429
H	3.61481501906043	3.73182491537116	-2.61881462262760
C	3.14279962366597	1.49460411164179	-0.92641673317769
C	4.43451792169622	1.86668430644588	-0.50254061487364
H	4.72256847883316	2.92950713273510	-0.52788024336387
H	6.35849893941031	1.23054031910936	0.28527671751780
H	1.89890719102418	3.99209767641426	-3.06098705979667
C	2.18126162594998	2.58825672014567	-1.39439759689182
H	1.19517667305839	2.11067975059930	-1.56776914818676
H	1.23219310723993	4.41324865167455	-0.63190682666344
C	1.99201127756345	3.67039196365949	-0.31203938458651
H	2.93302890894665	4.22724029561501	-0.12075542860282
H	1.66153879556491	3.22862312285812	0.64881440333624

[Sn(^{Dipp}NacNac)(CP)]

BP86/ZORA-def2-SVP, SARC-ZORA-SVP(Sn)

Sn	-0.03936760080490	0.02610157590724	0.11488187702016

C	-0.02417776091292	-0.17747811710072	2.34063772830994
P	-0.03066133329723	-0.04895893738599	3.91697661097108
N	1.57681531939298	-1.54671169606572	-0.07400153635872
N	-1.52592041648660	-1.66776216678702	-0.07991327499421
C	1.36214057191539	-2.83017821418636	0.25611890563461
C	0.09363146315883	-3.39671707694878	0.54811586827005
C	-1.21800542858492	-2.91960555575604	0.30151169204673
C	2.54725471849204	-3.78041631450586	0.28989271449172
C	-2.34273505235356	-3.93139389718985	0.44139411403468
C	2.87858177936704	-1.09788810987229	-0.49435045783798
C	3.18534049230283	-1.04608403253245	-1.88774301673175
C	4.46696416052800	-0.61257590732872	-2.28339893880804
C	5.42830274538946	-0.22041929393293	-1.34408204252763
C	5.10452395484898	-0.24107581634371	0.01783796122010
C	3.83944141111060	-0.66778819534588	0.47137908701475
C	2.17422519140090	-1.43383791201350	-2.96739163463566
C	1.90850115986928	-0.25583319353441	-3.92975676113809
C	2.61377879221382	-2.69415535332478	-3.74462523952866
C	3.54067527488066	-0.61660780397759	1.96988101518873
C	4.53693822708151	-1.44787290294285	2.80708565334590
C	3.50703889676065	0.84506006003559	2.46916164875365
C	-2.84509466157311	-1.34296497026030	-0.55531906728627
C	-3.81497605301265	-0.78642060306120	0.33202330286223
C	-5.08551571143461	-0.45152467126029	-0.17997416298671
C	-5.40920841543366	-0.65206230002108	-1.52712884470075
C	-4.44727232903386	-1.18987481485342	-2.39138395519661
C	-3.16050739587539	-1.53926916099916	-1.93472431625858
C	-3.52470607169570	-0.53484939797145	1.81071934962930
C	-4.50156491564516	-1.28933780171813	2.73835155373295
C	-3.52682510495474	0.97590045353118	2.13063301667812

C	-2.14995900990076	-2.09687786353598	-2.93820870771017
C	-1.82993890251843	-1.06081793373452	-4.03723176990829
C	-2.62215667176663	-3.42698711817929	-3.56516271894536
H	0.13531546224498	-4.43844053355654	0.88008749134934
H	3.04094336322863	-3.82333006295882	-0.69750088235236
H	2.23390372878435	-4.79783950980981	0.56964591182851
H	3.31341510052852	-3.43418510311195	1.00506661089545
H	-2.81035479377905	-4.14135959045079	-0.53720888778697
H	-3.14308950550115	-3.53982639260991	1.09325323700502
H	-1.97296591599129	-4.88040421703255	0.85949292287920
H	4.71396042326775	-0.58084341448890	-3.35265505491320
H	6.42139606533381	0.11098676416646	-1.67277642767406
H	5.85063072827759	0.08593752298275	0.75367542767229
H	1.22181901902141	-1.67335232206618	-2.45897404248897
H	1.14639521862557	-0.52795683867930	-4.68183478425884
H	2.82430659624888	0.03579723633371	-4.47636081831894
H	1.54727312819507	0.63432641505275	-3.38435257955899
H	2.76829488063810	-3.55634149068377	-3.07157953469814
H	3.56179425650838	-2.52202811527940	-4.28777894261762
H	1.84905770196680	-2.98066018716293	-4.49005601982233
H	2.53115345794356	-1.03623533609811	2.13210459226743
H	4.58479155875297	-2.49973445750671	2.47181357165516
H	4.23668436804239	-1.44787516198989	3.87088286636484
H	5.56235699136021	-1.03680017492795	2.75263289894348
H	2.77648743794073	1.45008554193234	1.90375985195606
H	4.49737055032333	1.32764851045102	2.36752696585329
H	3.21392681100033	0.88363597510585	3.53419076137064
H	-5.83695655313969	-0.02540292428761	0.49755455588285
H	-6.40508796401601	-0.38627960197495	-1.90359335092502
H	-4.69856574969247	-1.34006408071091	-3.44967904377715

H	-2.50799417366156	-0.91073190035519	2.02540159240387
H	-4.21093960997853	-1.15069987226530	3.79580843523450
H	-4.50973816172840	-2.37476607241886	2.53079588076417
H	-5.53927384041404	-0.92262553944053	2.62742897031307
H	-3.23173310456755	1.14976340746431	3.18134912993361
H	-4.52817868406903	1.42057323583252	1.97652165159929
H	-2.81456733368369	1.52738204967817	1.49051842658600
H	-1.21253682186471	-2.30110321195219	-2.38939971943480
H	-1.06983261531458	-1.45580485449444	-4.73605761991924
H	-1.44402771273185	-0.12062369979722	-3.60518298495846
H	-2.72912982855291	-0.81203041087072	-4.63090303247657
H	-1.84680681439087	-3.83724891261810	-4.23823230699769
H	-3.54228377822824	-3.28844242739456	-4.16296299808783
H	-2.83864521035594	-4.19005519880940	-2.79647837534210

[Au(IDipp)(CP)]

BP86/ZORA-def2-SVP, SARC-ZORA-SVP(Au)

Au	0.06536622884739	-0.02556382889657	0.04542566026438
C	0.02946620828291	0.05631206901257	-1.98549526626288
P	0.12710501915219	-0.15041340706852	3.58751487847547
N	1.09898653400152	0.11557648739624	-2.84691378760153
C	0.67587931996601	0.17017207265569	-4.17300990065749
C	-0.69494310113371	0.14339530190458	-4.14955682261254
N	-1.07019645721331	0.07403148449903	-2.80982112473593
C	0.09984661721167	-0.09625881717785	2.00678456663518
H	1.38036275524124	0.21954600850285	-5.00318908952408
H	-1.42844292538718	0.16508107181492	-4.95559130836982
H	-1.86432884057172	4.23245343530778	-0.92913401483660

H	-2.71317199571415	4.43365429831638	-3.35627192355398
C	-2.38557816262406	3.26218828124725	-0.83624180381636
H	-1.82086515309498	2.63374708909609	-0.12442179101296
H	-1.47022824665916	2.43389411136790	-2.59242467522806
C	-3.23927256045665	3.46931976724317	-3.23532442554058
C	-2.49960275142986	2.57651601857418	-2.21560426933455
H	-3.38267096295659	3.45846419659739	-0.40124592011928
H	-4.26978198655947	3.69575049845739	-2.90601740942285
H	-3.30346954941273	2.98826513106253	-4.22808715071937
C	-3.14622084997110	1.19769297359054	-2.09114230868529
C	-2.44305744343734	-0.00583528454403	-2.35558150143736
H	-5.05963817522741	1.99471785954931	-1.45319846201578
C	-4.48803274086141	1.08481094955128	-1.67429050435902
C	-3.03357784807269	-1.28812181743260	-2.20882786067649
C	-5.10053898645201	-0.16672667809812	-1.52543508123675
C	-4.37831353198480	-1.33828822197125	-1.78890021296335
H	-6.14406887223112	-0.22946422280247	-1.19364895923490
H	-4.86400956321996	-2.31338152979708	-1.65696399719569
H	-1.61884542489108	-2.80111929204142	-0.37288329077673
C	-2.10715331108321	-3.40046775189909	-1.16198918701350
H	-3.08690693917830	-3.73761407859546	-0.77630312596113
H	-1.49125639520634	-4.30021690255940	-1.34231519393639
C	-2.26570884423515	-2.58440864433942	-2.46320307007910
H	-1.24890831786639	-2.31298312583233	-2.80112276681537
C	-2.91331488434910	-3.42379544615611	-3.58508078489244
H	-3.92646077809831	-3.76407049752643	-3.30328035558651
H	-2.30831964260923	-4.32500332033626	-3.79314950005674
H	-3.00404352889812	-2.84932764714594	-4.52484617461306
H	3.12891451518652	-2.76300121088051	-4.61567111059709
C	3.06542348853207	-3.32834015606649	-3.66813634724122

H	2.48303848051314	-4.24828506173694	-3.85776361225920
H	4.09062933526730	-3.63829460284423	-3.39516898084686
H	1.37772940772231	-2.26144055275320	-2.87358124994554
C	3.13480269258673	-1.16935519969734	-2.32086401395251
C	2.40813410251857	-2.49447701208911	-2.54814719422716
C	4.49446883317559	-1.16720543227031	-1.94904568160756
C	5.17864488844420	0.03137777315779	-1.70529688906704
H	5.02268233955441	-2.12266978716308	-1.83930335813469
H	6.23490693800840	0.00899331540134	-1.41048122598586
C	2.29728982187452	-3.29659995318211	-1.23297327143753
H	1.70902998631000	-4.21877136173970	-1.39082046487963
H	3.29418240048892	-3.59445144318369	-0.85906735410636
H	1.80165963879293	-2.70203160328721	-0.44479323753985
H	3.12609080261660	3.09715605229488	-4.34538994231322
H	2.53266753735303	4.52677586364430	-3.44941014517611
C	3.09835580154140	3.58250308276234	-3.35276777832047
H	4.13711237135432	3.84569831446830	-3.08136114514198
C	2.44810435692359	2.67113093860322	-2.28957673001593
H	1.40595990684353	2.48622080113028	-2.60826002235870
C	2.48888062545411	0.08901360853900	-2.44053670535741
C	3.15261061830964	1.31851118821523	-2.19399264608602
H	1.82332000083374	4.31283519936534	-0.98041796232329
C	2.38378208636708	3.36289221418391	-0.91014034182850
H	3.39492363407655	3.59849313916189	-0.53040634889455
C	4.51192399765269	1.25796087718118	-1.82567849285332
H	5.05406409745746	2.18921627142488	-1.61946026761330
H	1.88080338262437	2.72052614583332	-0.16534956437955

[Co(^{Dipp}PDI)(CP)]

BP86/def2-SVP

Co	0.02242812082611	-0.01414448841884	-0.01475750117200
C	0.04491331750559	-0.02246200120192	1.79779096022292
P	0.06221858683681	-0.03557243952396	3.39075577881395
N	-0.00147563850378	-0.00478700093810	-1.85279069783994
C	1.18653098151177	0.01486174861897	-2.53319835784683
C	1.19415100202576	0.02382010860357	-3.94150031012802
H	2.14584946779806	0.04296588525977	-4.49057806789048
C	-0.03746787232263	0.00879360551676	-4.62483753196510
H	-0.05173163705749	0.01401870165450	-5.72451758273042
C	-1.25089446616417	-0.01304516063394	-3.90995028933266
H	-2.21651836603737	-0.02721125583029	-4.43432539267469
C	-1.20678491398042	-0.01776843353764	-2.50226433416743
C	2.29405707803238	0.01310510063597	-1.59222725782882
N	1.89008931882707	0.00077285206136	-0.31921917931060
C	3.72405557876632	0.00844581790008	-2.03608691634065
H	3.89741557707190	-0.78989514826737	-2.78749536650866
H	4.40567901651364	-0.14847503471811	-1.18031543076296
H	3.99263411102490	0.97199472463986	-2.52101391951306
C	-2.28942033980418	-0.02591943671016	-1.53279532609858
N	-1.85217038455701	-0.02665147968422	-0.27073280633795
C	-3.73066161759845	-0.01855896805324	-1.93849371633936
H	-4.01333549810071	-0.98094766998826	-2.41777274395344
H	-3.92315721627927	0.78146702124876	-2.68334483182958
H	-4.38897879867289	0.13697579580969	-1.06437281364600
C	2.83598727372883	-0.00202024082144	0.75196994787911
C	3.02523414608419	-1.21396775785207	1.46180470067772
C	3.90615879227092	-1.20346553741990	2.55707739160757

H	4.06880980260577	-2.12872497731019	3.12835796844275
C	4.55849559195431	-0.02536466452173	2.94351289589033
H	5.24054346162685	-0.03220922498156	3.80743887847435
C	4.32980792594851	1.16883610103146	2.24479018336703
H	4.82853475467626	2.09080885331913	2.57580649107248
C	3.46291794975507	1.21064123462818	1.13686780663775
C	2.27119187128372	-2.46375945739806	1.03070902370147
H	1.31934297273278	-2.09325079678685	0.58562623135499
C	1.89232376148023	-3.37850888945406	2.19962655140833
H	1.24152523685468	-4.20285252893638	1.84357511507194
H	1.33729060797366	-2.81034307522944	2.97317013328400
H	2.78031356549215	-3.84447322052626	2.67628243266703
C	3.02999718997938	-3.22710555280159	-0.07105354820955
H	3.23891021241393	-2.58085293025342	-0.94663507657229
H	2.43881946658209	-4.09654668148237	-0.42666716794351
H	4.00371588739097	-3.60498101556864	0.30595706656502
C	3.11914211067766	2.52039723922934	0.43225858339473
H	3.01887794497846	2.30176497843513	-0.65253959029341
C	4.19925649041676	3.60139754952787	0.57476774357897
H	3.94710389440176	4.47906185739492	-0.05435927500304
H	5.19829797089124	3.23138288090330	0.26562511116109
H	4.27993144592521	3.96609151870949	1.61979741966869
C	1.74592957557438	3.03330118290788	0.91246211260486
H	1.46329784016775	3.96590780517702	0.38123114135589
H	1.76202245794938	3.23589640886310	2.00265259363963
H	0.95820707107300	2.27280281239326	0.73564631304608
C	-2.76992938954111	-0.03611977117605	0.82465395778646
C	-2.94123914278396	1.16816465627015	1.55202485493586
C	-3.79343872774276	1.14527827444895	2.66960207665060
H	-3.94164983163508	2.06425691547147	3.25477342876143

C	-4.43467154921302	-0.03747377428201	3.06041822543450
H	-5.09382252186134	-0.04045609080827	3.94196539089632
C	-4.22347013617342	-1.22405113711444	2.34341341192370
H	-4.71266523630298	-2.14997495776831	2.67764703008698
C	-3.38588362310668	-1.25326283539595	1.21278771165285
C	-2.19805989730298	2.42248724472346	1.11538657656899
H	-1.25587432046164	2.05643147612196	0.64644428443822
C	-1.79415820187801	3.32753218266691	2.28346974851063
H	-1.15179348188253	4.15526800626993	1.92008372267066
H	-1.22189198778466	2.75351151731480	3.03996796264034
H	-2.67175986807663	3.78891473321425	2.78332531709033
C	-2.98106855920295	3.19463577495814	0.03697981667997
H	-2.39871431102216	4.06832696028068	-0.32268555698310
H	-3.94738303594040	3.56718474483044	0.43762650282468
H	-3.20691098940262	2.55615086349901	-0.84009223887957
C	-3.05957406251937	-2.55522763128848	0.48572685442274
H	-2.98522728012619	-2.32450604763578	-0.59861544501161
C	-1.67546248954364	-3.07349492845049	0.92719220380845
H	-0.89186681816434	-2.31125635565123	0.74000411556383
H	-1.40608296619849	-4.00028423821187	0.37914198359943
H	-1.66545728098942	-3.28809431265793	2.01516979056095
C	-4.13646350763743	-3.63752004982896	0.64214442910001
H	-3.90004287555861	-4.50822468513121	-0.00257818887617
H	-5.14248670253505	-3.26380925658142	0.36136068637570
H	-4.19217388596440	-4.01379399370710	1.68467580341712

5. References

[1] M. Ma, A. Stasch and C. Jones, *Chem. Eur. J.* **2012**, *18*, 10669–10676.

[2] *CrysAlisPro*, Agilent Technologies, Version 1.171.35.8.

[3] (a) G. M. Sheldrick in SHELXL97, *Programs for Crystal Structure Analysis (Release 97-2)*, Institut für Anorganische Chemie der Universität, Tammanstrasse 4, D-3400 Göttingen, Germany, 1998; (b) G. M. Sheldrick, *Acta Crystallogr. Sect. A* 1990, **46**, 467−473; (c) G. M. Sheldrick, *Acta Crystallogr. Sect. A* 2008, **64**, 112−122.

[4] F. Neese, *Wiley Interdiscip. Rev. Comput. Mol. Sci.* **2012**, *2*, 73–78.

[5] F. Neese, *Wiley Interdiscip. Rev. Comput. Mol. Sci.* **2018**, *8*, DOI 10.1002/wcms.1327.

[6] A. D. Becke, *Phys. Rev. A* **1988**, *38*, 3098–3100.

[7] J. P. Perdew, *Phys. Rev. B* **1986**, *33*, 8822–8824.

[8] F. Neese, *J. Comput. Chem.* **2003**, *24*, 1740–1747.

[9] F. Weigend, R. Ahlrichs, *Phys. Chem. Chem. Phys.* **2005**, *7*, 3297–3305.

[10] F. Weigend, *Phys. Chem. Chem. Phys.* **2006**, *8*, 1057–1065.

[11] S. Grimme, S. Ehrlich, L. Goerigk, *J. Comput. Chem.* **2011**, *32*, 1456–1465.

[12] H. Kruse, S. Grimme, *J. Chem. Phys.* **2012**, *136*, 154101.

[13] D. A. Pantazis, X. Y. Chen, C. R. Landis, F. Neese, *J. Chem. Theory Comput.* **2008**, *4*, 908–919.

[14] M. Bühl, C. Reimann, D. A. Pantazis, T. Bredow, F. Neese, *J. Chem. Theory Comput.* **2008**, *4*, 1449–1459.

[15] S. Grimme, J. G. Brandenburg, C. Bannwarth, A. Hansen, *J. Chem. Phys.* **2015**, *143*, 054107.

[16] F. London, *J. Phys. Radium* **1937**, *8*, 397–409.

[17] W. J. Hehre, K. Ditchfield, J. A. Pople, *J. Chem. Phys.* **1972**, *56*, 2257–2261.

[18] T. Helgaker, M. Jaszuński, K. Ruud, *Chem. Rev.* **1999**, *99*, 293–352.

[19] C. Adamo, V. Barone, *J. Chem. Phys.* **1999**, *110*, 6158–6170.

[20] F. Jensen, *J. Chem. Theory Comput.* **2015**, *11*, 132–138.

[21] I. A. Konstantinov, L. J. Broadbelt, *J. Phys. Chem. A* **2011**, *115*, 12364–12372.

[22] A. E. Aliev, D. Courtier-Murias, S. Zhou, *J. Mol. Struct. THEOCHEM* **2009**, *893*, 1–5.

[23] R. Jain, T. Bally, P. R. Rablen, *J. Org. Chem.* **2009**, *74*, 4017–4023.

[24] P. R. Rablen, S. A. Pearlman, J. Finkbiner, *J. Phys. Chem. A* **1999**, *103*, 7357–7363.

[25] S. K. Latypov, F. M. Polyancev, D. G. Yakhvarov, O. G. Sinyashin, *Phys. Chem. Chem. Phys.* **2015**, *17*, 6976–6987.

[26] V. Barone, M. Cossi, *J. Phys. Chem. A* **1998**, *102*, 1995–2001.

[27] D. M. York, M. Karplus, *J. Phys. Chem. A* **1999**, *103*, 11060–11079.
